# Comprehensive Analysis of Necroptosis Landscape in Skin Cutaneous Melanoma for Appealing its Implications in Prognosis Estimation and Microenvironment Status

**DOI:** 10.3390/jpm13020245

**Published:** 2023-01-29

**Authors:** Xiaoying Cao, Jiaming He, An Chen, Jianhua Ran, Jing Li, Dilong Chen, Hengshu Zhang

**Affiliations:** 1 Department of Plastic and Burn Surgery, The First Affiliated Hospital of Chongqing Medical University, Chongqing 400016, China; 2 Laboratory of Stem Cells and Tissue Engineering, College of Basic Medical, Chongqing Medical University, Chongqing 400016, China; 3 Neuroscience Research Center, College of Basic Medical, Chongqing Medical University, Chongqing 400016, China; 4 Chongqing Key Laboratory of Development and Utilization of Genuine Medicinal Materials in Three Gorges Reservoir Area, Chongqing Three Gorges Medical College, Chongqing 404120, China

**Keywords:** skin cutaneous melanoma (skcm), necroptosis, prognosis, bioinformatics, tumor microenvironment

## Abstract

Purpose: Due to poor prognosis and immunotherapy failure of skin cutaneous melanoma (SKCM), this study sought to find necroptosis-related biomarkers to predict prognosis and improve the situation with predicted immunotherapy drugs. Experimental Design: The Cancer Genome Atlas (TCGA) and The Genotype-Tissue Expression Program (GTEx) database were utilized to recognize the differential necroptosis-related genes (NRGs). Univariate Cox (uni-Cox) and least absolute shrinkage and selection operator (LASSO) Cox analysis were utilized for prognostic signature establishment. The signature was verified in the internal cohort. To assess the signature’s prediction performance, the area under the curve (AUC) of receiver operating characteristic (ROC) curves, Kaplan-Meier (K-M) analyses, multivariate Cox (multi-Cox) regression, nomogram, and calibration curves were performed. The molecular and immunological aspects were also reviewed using single-sample gene set enrichment analysis (ssGSEA). Cluster analysis was performed to identify the different types of SKCM. Finally, the expression of the signature gene was verified by immunohistochemical staining. Results: On basis of the 67 NRGs, 4 necroptosis-related genes (FASLG, PLK1, EGFR, and TNFRSF21) were constructed to predict SKCM prognosis. The area’s 1-, 3-, and 5-year OS under the AUC curve was 0.673, 0.649, and 0.677, respectively. High-risk individuals had significantly lower overall survival (OS) compared to low-risk patients. Immunological status and tumor cell infiltration in high-risk groups were significantly lower, indicating an immune system that was suppressed. In addition, hot and cold tumors could be obtained by cluster analysis, which is helpful for accurate treatment. Cluster 1 was considered a hot tumor and more susceptible to immunotherapy. Immunohistochemical results were consistent with positive and negative regulation of coefficients in signature. Conclusion: The results of this finding supported that NRGs could predict prognosis and help make a distinction between the cold and hot tumors for improving personalized therapy for SKCM.

## 1. Introduction

Skin cutaneous melanoma (SKCM) is a destructive malignant tumor and one of the important obstacles to extending life expectancy [1]. In 2020, 324,635 new cases were diagnosed with melanoma worldwide, and 57,043 patients died from the disease [2]. And its characteristic is featured by complicated mutation load, and a highly immunogenic microenvironment, showing different sensitivity to immunotherapy. Besides, the antibody-mediated blockade of the programmed cell-death protein 1 (PD-1) immune checkpoints treatment only showed response rates ranging from 20% to 40% in melanoma patients [3]. In general, malignant melanoma has been divided into four histopathological subtypes: superficial spreading melanoma (SSM), nodular melanoma (NM), acral lentiginous melanoma (ALM), and lentigo maligna melanoma (LMM) [4]. Although primary histology types have obvious clinical and biological differences, the effect of these subtypes on immunotherapy remains unclear [5]. Multiple studies have classified melanoma according to the expression of characteristic genes, showing good guidance for the efficacy of targeted and immune therapies [6,7,8].

Necroptosis is a recently discovered form of cell death that is similar to necrosis in that it is characterized by the loss of cell membrane integrity and the release of cellular contents into the surrounding tissue. However, necroptosis is a form of programmed cell death that is triggered by specific cellular signaling pathways [9]. RIPK1 and RIPK3, as well as their target, MLKL, are required to initiate necroptosis [10,11,12]. Research has shown that necroptosis enhances CD8+ leukocyte-mediated anti-tumor immunity by activating RIPK1 and RIPK3 in the tumor microenvironment (TME) [13]. Seifert L et al. have shown that necroptosis promotes macrophage-induced immunosuppression of T cells, thereby inhibiting tumor cell metastasis in pancreatic cancer [14]. Since necroptosis represents a novel cell death form controlled by the specific signal transduction pathways, it provides a molecular target for therapeutic interventions [15]. Accumulating evidence revealed that necroptosis is associated with a variety of human diseases, including inflammatory, neurodegenerative, autoimmune diseases, and cancer, strengthening the concept that targeting necroptosis in cancer could be a viable therapeutic method [16,17]. Besides, a novel naphthalene derivative has shown promise in the treatment of melanoma by inducing necroptosis [18]. 

However, the precise role of necroptosis regulators in the prognosis of SKCM and underlying molecular mechanisms remains unknown. Therefore, it is imperative and attractive to develop clinical signatures of necroptosis to assist cancer treatment. Meanwhile, we identified the immune microenvironment and potential drug treatment modalities of SKCM patients under novel subtypings.

## 2. Materials and Methods

### 2.1. Online Data

TCGA database was utilized to obtain RNA transcriptome (RNA-seq, FPKM) data for 1 normal skin tissue and 374 melanoma cases, as well as related clinical information and Copy number variation (CNV) data (https://portal.gdc.cancer.gov/repository, accessed on 15 January 2022). After entering the website, click the “Repository” button, and select the SKCM sample after the web page jumps. Then, users could selected the “transcriptome profiling”, “clinial”, and “copy number variation” to downloaded RNA-seq, clinical information, and CNV data, respectively. The GTEx database was used to obtain the other 233 RNA-seq normal skin tissues (ID: Skin, Not Sun Exposed, https://www.xenabrowser.net/, accessed on 15 January 2022). In UCSC XENA website, users needed to select the datasets module to jump to GTEX project. Next, click the “GTEX phenotype” button to extracted normal skin transcriptome data. The TCGA and GTEx datasets were merged and batch corrected for subsequent analysis. 

### 2.2. Clinical Tissue Specimens

The three cases of human SKCM tumors and normal tissues were collected from the First Affiliated Hospital of Chongqing Medical University which never received any preoperative radiotherapy or chemotherapy (2022.02–2022.06). All human tissues were collected by national and institutional ethical guidelines and approved by the Ethics Committee of First Hospital of Chongqing Medical University (2022-K31).

### 2.3. Analysis of Differentially NRGs

The Gene Set Enrichment Analysis (GSEA) database was used to obtain eight necroptosis genes (gene set: M24779.gmt, http://www.gsea-msigdb.org/gsea/index.jsp, accessed on 15 January 2022). Furthermore, from previous research, we finally extracted 67 NRGs which are listed in Table S1 [19,20,21]. Then, genes were divided into mRNAs and lncRNAs by Perl. Necroptosis-related lncRNAs were obtained by Spearman correlation. And, differentially expressed necroptosis-related mRNAs (DNRGs) were extracted by using the “limma” R package (|log2FC| ≥ 1 and *p* < 0.05). The correlation between the differential expression of necroptosis-related mRNAs and lncRNAs in the merged matrix was analyzed (coefficients > 0.4, and *p* < 0.001). 

### 2.4. Genetic Mutation and Expression Variation Analysis

The “maftools” package was performed to show the mutation frequency and oncoplot waterfall plot of DNRGs. The location of CNV alteration was performed using the “RCircos” R package.

### 2.5. Construction and Validation of the necroptosis-Related Signature

Utilizing the clinical data of GTEx and TCGA samples, uni-Cox analyses were performed to identify NRGs with survival differences (*p* < 0.05). Next, a 10-fold cross-validated Lasso regression was performed. To prevent overfitting, a random stimulation was set up 1000 times each cycle. Then, a necroptosis-related signature was constructed. The following formula was used to calculate the risk score:

$$risk\;score=\overset{n}{\underset{k=0}{\sum }}coef\left(gene^{k}\right)*expr\left(gene^{k}\right)$$

The coef (gene) was an abbreviated form of the coefficient gene. The expr (gene) was the expression of the gene. Sample in the model were divided into low- and high-risk groups based on the median risk score. The ROC curves were performed by “timeROC”, “survminer”, and “survival” R packages. The chi-square test was used to analyze the relationship between the model and clinical features, as well as to assess the prognostic value of the constructed signature.

### 2.6. Independence Factors and ROC

Uni-Cox and multi-Cox analyses were performed to determine whether the risk score and clinical characteristics were independent variables in TCGA-SKCM cohort. ROC analysis was utilized to compare the effects of clinical factors on prognosis.

### 2.7. Nomogram and Calibration

The clinicopathological information (age, gender, T, M, N, tumor stage) and risk score were utilized to set up a nomogram for the 1-, 3-, and 5-year OS of SKCM patients by using the “rms” R package. Then, correction curves based on the Hosmer-Lemeshow test to illustrate whether the prediction outcome showed good consistency with the practical. 

### 2.8. Gene Set Enrichment Analyses

The GSEA software (Version: 4.1.0) was used to identify the significantly enriched pathways and functions between different subgroups. After submitting the transcriptome matrix and selecting genesets, and performed 1000 permutation (genesets: Kegg.v7.4.symbols.gmt, *p* < 0.05 and FDR < 0.25).

### 2.9. TME and Immune Checkpoints Analyses

The available computational algorithms for immune infiltration estimation fall into two main categories: gene signature- (xCell, MCP-counter) and deconvolution- (CIBERSORT, TIMER, EPIC, quanTIseq) based approaches. Then, we calculate the immune infiltration statuses among the SKCM patients using these algorithms. And, the ssGSEA analysis was applied. Wilcoxon signed-rank test, “limma”, “scales”, “ggplot2”, and “ggtext” R packages were used to analyze the differences in the content of immune infiltrating cells. The results were displayed in a bubble chart. Furthermore, TME scores and immune checkpoint activation between the two subgroups were also determined using the “ggpubr” R package.

### 2.10. Potential Therapeutic Medicine

To determine the immunotherapy response of each SKCM patient, the half-max inhibitory concentration (IC_50_) was determined using the the “Limma”, “ggpubr”, “ggplot2”, and “pRRophetic” R package (https://www.cancerrxgene.org/, accessed on 15 January 2022).

### 2.11. Clusters Based on Four Prognostic Signature Genes

The TCGA-SKCM patients were classified by using the “ConsensusClusterPlus” R package based on prognostic gene expression. Principal component analysis (PCA), T-distributed stochastic neighbor embedding (t-SNE), and K-M analysis were constructed to verify the discrimination and accuracy of the subtypes by using “survival” and “survminer” R package. 

### 2.12. Immunohistochemical Staining (IHC)

Paraffin sections were dewaxed and rehydrated. Next, endogenous peroxidase and nonspecific binding sites were blocked with 10% bovine serum albumin (AR0009, Boster, Wuhan, China) for 60 min. Then, all sections were incubated with rabbit anti-FASL (1:200, Abways), EGFR (1:500, Abcepta, Suzhou, China), DR6 (1:500, Abcepta, Suzhou, China), and PLK1 (1:500, Proteintech, Wuhan, China) antibody overnight at 4 °C. Subsequently, binding was conducted with the corresponding peroxidase-conjugated secondary antibody (A21020, Abbkine, Wuhan, China) and incubated at 37 °C for 30 min. 

## 3. Results

### 3.1. The Genetic Mutation Landscape and Expression of Necroptosis Genes in SKCM

**Figure 1:**
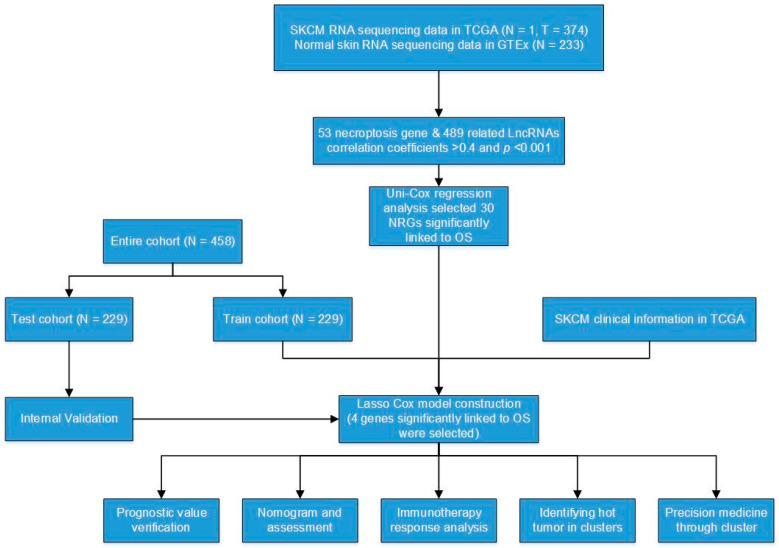
Flow diagram of the study.

**Figure 2:**
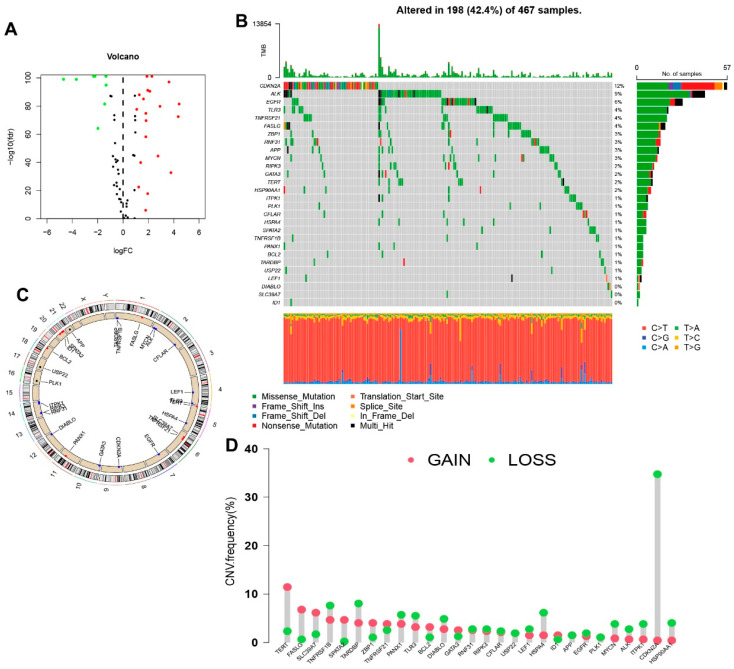
Expression and genetic mutation of necroptosis regulators in SKCM. (**A**) The volcano map indicated the expression of necroptosis genes in SKCM (red: High expression, green: Low expression). (**B**) The waterfall plot indicated mutation frequency and classification of necroptosis genes in TCGA-SKCM cohort. (**C**) The circle plot shows the location of necroptosis regulators CNV on chromosomes. (**D**) The lollipop chart display the CNV frequency of necroptosis genes in SKCM. The height of the column represented the alteration frequency.

The research process of the study is shown in Figure 1. After extracting GTEx and TCGA matrix, we obtained 175 normal skin tissues and 471 SKCM samples. According to the expression of differentially expressed mRNAs and lncRNAs between normal and SKCM samples, we finally obtained 53 DNRGs and 489 related lncRNAs (correlation coefficients >0.4 and *p* < 0.001). Of the DNRGs, 20 mRNAs (HSPA4, APP, USP22, TLR3, PANX1, MYCN, TNFRSF1B, BCL2, HSP90AA1, SPATA2, ITPK1, ZBP1, PLK1, SLC39A7, TERT, LEF1, TNFRSF21, CDKN2A, FASLG, ALK) were upregulated, and 8 mRNAs (GATA3, EGFR, DIABLO, CFLAR, ID1, RIPK3, TARDBP, RNF31) were downregulated (|Log2FC| > 1, FDR = 0.05 and *p* < 0.05) (Figure 2A). The network figure and data between necroptosis-related mRNAs and lncRNAs were drawn and listed in Figure S1 and Table S2. The genetic landscape of DNRGs in the waterfall plot was performed by the “maftools” R package in Figure 2B. The waterfall plot is a visualization tool for presenting gene mutation data. We show the mutations of DNRGs in TCGA-SKCM samples, with each point representing a sample and the color representing the mutation type. Waterfall plots can help researchers rapidly identify high-frequency mutation loci and significant mutation patterns. Figure 2C,D illustrate the significance of CNV and the relationship between it and the location of necroptosis regulators on the chromosome.

### 3.2. Construction and Validation of a Necroptosis-Related Prognostic Signature

**Figure 3:**
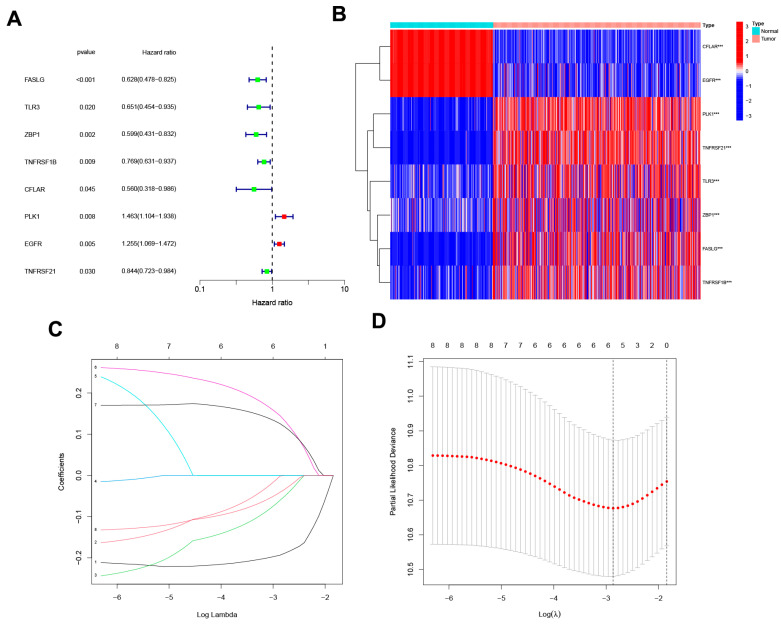
The development of a necroptosis-related gene signature. (**A**) The forest plot shows the extraction of prognostic genes using Uni-Cox regression analysis. (**B**) The heat map shows the expression profiles of 8 prognostic genes. Wilcoxon signed-rank test, *** *p* < 0.001. (**C**) The LASSO model employs 10-fold cross-validation to exclude collinearity from severe variable optimization and simplification models. (**D**) Partial-likelihood deviance changes the curve with Log(λ), and the smaller this value indicates that the model fits better.

**Figure 4:**
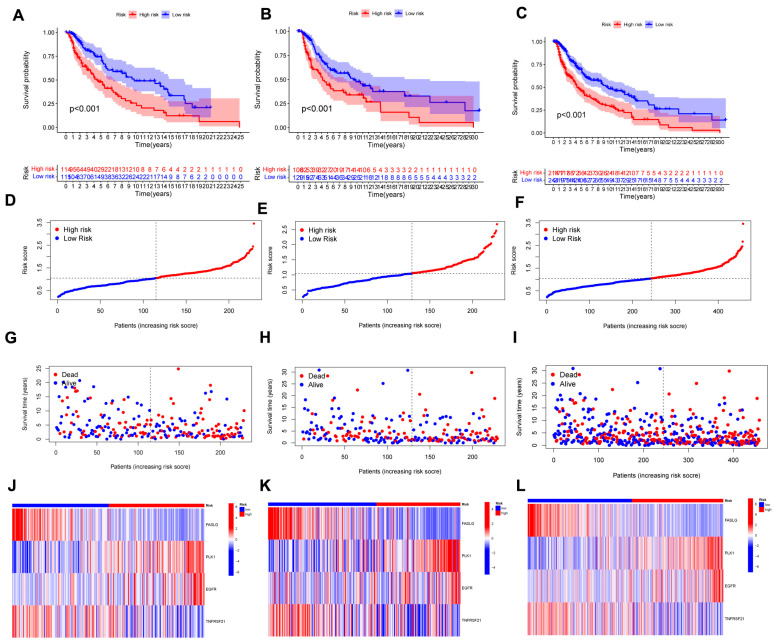
The prognostic performance of the 4 necroptosis-related genes model. (**A**–**C**) K-M analysis depicting the OS between low- and high-risk groups in the train, test, and entire sets, respectively. (**D**–**F**) The distribution of the risk scores in the three cohorts. (**G**–**I**) Each patient’s survival status between low- and high-risk groups in the three cohorts. (**J**–**L**) The expression of 4 NRGs in the three cohorts.

The uni-Cox analysis (Figure 3A) determined that 8 genes related to necroptosis were significantly correlated with OS (*p* < 0.05). Then, we show the differential expression of these genes using a heat map in Figure 3B. To avoid overfitting of the prognostic signature, we performed Lasso regression on these genes which were associated with necroptosis in SKCM. Lasso regression is a type of linear regression that uses a regularization term known as the λ penalty. The λ penalty encourages the coefficients of the model to be sparse, meaning that many of them will be zero. This can be useful for feature selection in high-dimensional datasets, as it can help to identify the most important features. The Lasso regression algorithm is typically implemented by minimizing the mean squared error of the model subject to a constraint on the sum of the absolute values of the coefficients. By calculating the penalty parameter (λ) according to the minimum requirement, we found that the model could be constructed after retaining the 4 genes and their correlation coefficients (Figure 3C,D). The details of genes and coefficients were shown in Table 1. The risk score formula was used to compare low-risk and high-risk groups of patients in the training, testing, and complete sets. The distribution of risk score, survival status, and survival time, as well as relevant expression standards were analyzed. All findings showed that the prognosis was poorer for the high-risk group (Figure 4A–L). In addition, the same results were obtained in clinicopathological features which were extracted from the TCGA database (Figure S2).

### 3.3. Nomogram Analysis

**Figure 5:**
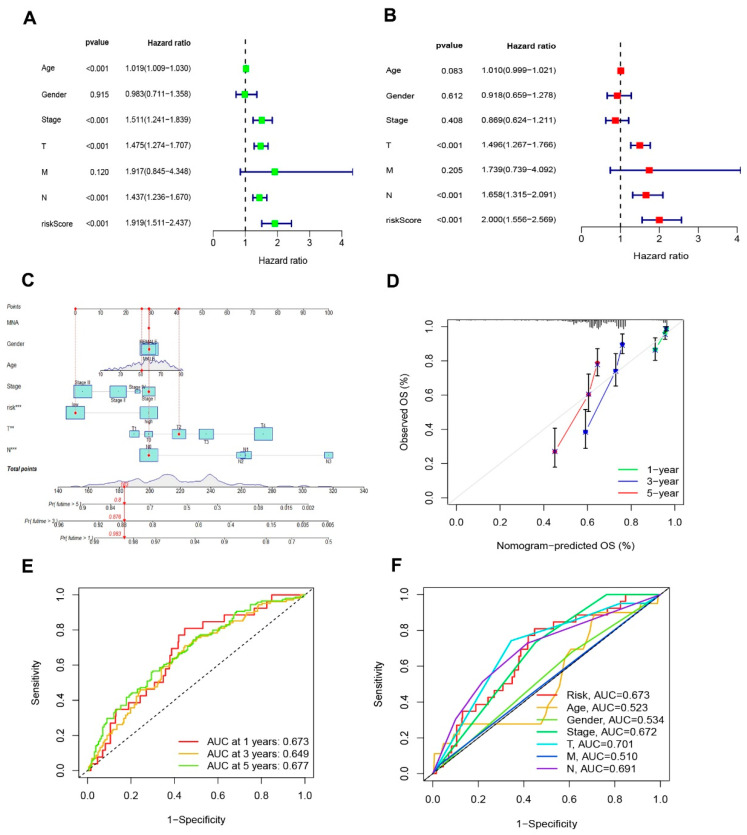
Nomogram and assessment of the risk signature. (**A**,**B**) Uni- and multi-Cox analyses of clinical characteristics and risk score with OS in TCGA-SKCM cohort, respectively. (**C**) The nomogram that integrated the T, N, and risk score predicted the probability of the 1-, 3-, and 5-year OS. (**D**) The calibration curves for nomogram. The ideal nomogram is shown by a dashed diagonal line. (**E**) The 1-, 3-, and 5-year ROC curves of the entire sets. (**F**) The 3-year ROC curves of risk score and clinical characteristics.

The hazard ratio (HR) of the risk score and 95% confidence interval (CI) in uni-Cox regression were 1.919 and 1.511–2.437 (*p* < 0.001, Figure 5A). The multi-Cox regression were 2000 and 1.556–2.569 (*p* < 0.001, Figure 4B), respectively. Furthermore, two other independent parameters were also found to be independent prognostic indicators, T stage (1.496 and 1.267–1.766; *p* < 0.001) and N stage (1.658 and 1.315–2.091; *p* < 0.001) (Figure 5B). Based on three independent prognostic factors (risk score, T stage, and N stage), a nomogram for predicting the 1-, 3-, and 5-year OS incidence of SKCM was developed (*p* <0.05 in multi-Cox) (Figure 5C). The quality of a prediction model can be evaluated by considering two aspects: discrimination and calibration. Discrimination is mainly used to reflect the differentiation ability of the prediction model, which is to evaluate how sure the model is to determine the ability of the predicted patient to occur the event. Calibration refers to the consistency or approximation degree between the actual probability of the outcome and the predicted probability. The former can be evaluated by AUC, while the latter can be evaluated by calibration chart. Then, a calibration plot was constructed for 1-, 3-, and 5-years to ascertain whether the nomogram demonstrated a good correlation with predictedOS (Figure 5D). According to the coincidence degree of the dashed line of the ideal model and the result of the model prediction line in Figure 4D, it suggested that the nomogram could be predicted relatively well in the entire cohort for the OS rates. However, we need to evaluate our signature further.

### 3.4. Evaluation of the Risk Signature

The sensitivity and specificity of the signature were evaluated by illustrating the AUC curves. The 1-, 3-, and 5-year AUCs of the entire set were 0.673, 0.649, and 0.677 (Figure 5E). On the basis of the 3-year ROC data, the clinical information and risk score had superior predictive abilities (Figure 5F). 

### 3.5. GSEA Analyses

**Figure 6:**
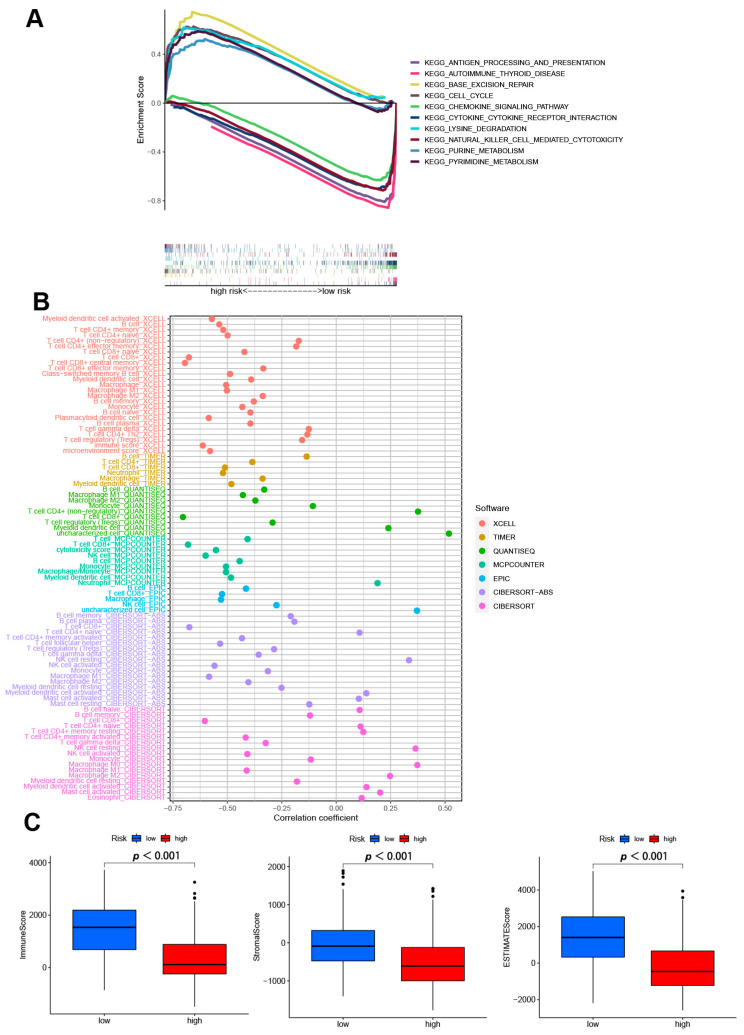
The exploring of tumor immune characteristics. (**A**) Top 5 pathways significantly enriched in the two subgroups by the GSEA algorithm. (**B**) The immune cell bubble of risk groups in different platforms. (**C**) The comparison of immune-related scores between two subgroups.

GSEA software was used to explore the differences in biological functions between two risk subgroups. The top five pathways in the high- and low-risk group in the entire set were performed in Figure 6A (*p* < 0.05; FDR < 0.25; |NES| > 1.5). In high-risk group, base excision repair, lysine degradation, purine metabolism, cell cycle, and pyrimidine metabolism were enriched. While in low-risk group, we found antigen processing and presentation, autoimmune thyroid disease, chemokine signaling pathway, cytokine-cytokine receptor interaction, and natural killer cell mediated cytotoxicity were highly expressed. These results suggest that disturbances in amino acid metabolism and cell cycle regulation are closely associated with high-risk patients. Conversely, low-risk patients were more likely to suffer from dysregulation of the immune and inflammatory environment. Therefore, according to the different potential biological backgrounds of patients with different subtypes, we need to further explore the immune microenvironment and possible therapeutic drugs between the subgroups.

### 3.6. Analysis of Immune Factors and Clinical Treatment in Risk Groups

Different platforms of the immune cell bubble chart indicated that the high-risk group had fewer immune cells, such as T cell CD4+ (non-regulatory) at QUANTISEQ, B cell memory, B cell plasma, T cell CD8+ naïve, NK cell resting, myeloid dendritic cell activated, mast cell activated at ABS, and B cell naïve, B cell memory, T cell CD4+ naïve, T cell CD4+ memory resting, M0 macrophage, M2 macrophage at CIBERSORT (*p* < 0.05) (Figure 6B, Table S3). Since significant differences between two subgroups were observed in the multi-platform immune infiltration, we further proceeded to analyze immune scores. The high-risk group had a lower immune score and a lower ESTIMATE (microenvironment) score compared to the low-risk group, suggesting a different TME (Figure 6C). Besides, ssGSEA also indicated the high-risk group had a lower immune function and immune infiltration status (Figure S3). In immune checkpoint analysis, the majority had better activation in low-risk groups (Figure S4). Also, we found there had several immunotherapeutic drugs with significant differences between the two subgroups (Figure S5). These results demonstrated the effectiveness of our signature in immunotherapy and clinical application.

### 3.7. Distinguishing between the Different Subtypes and Precision Medicine in Clusters

**Figure 7:**
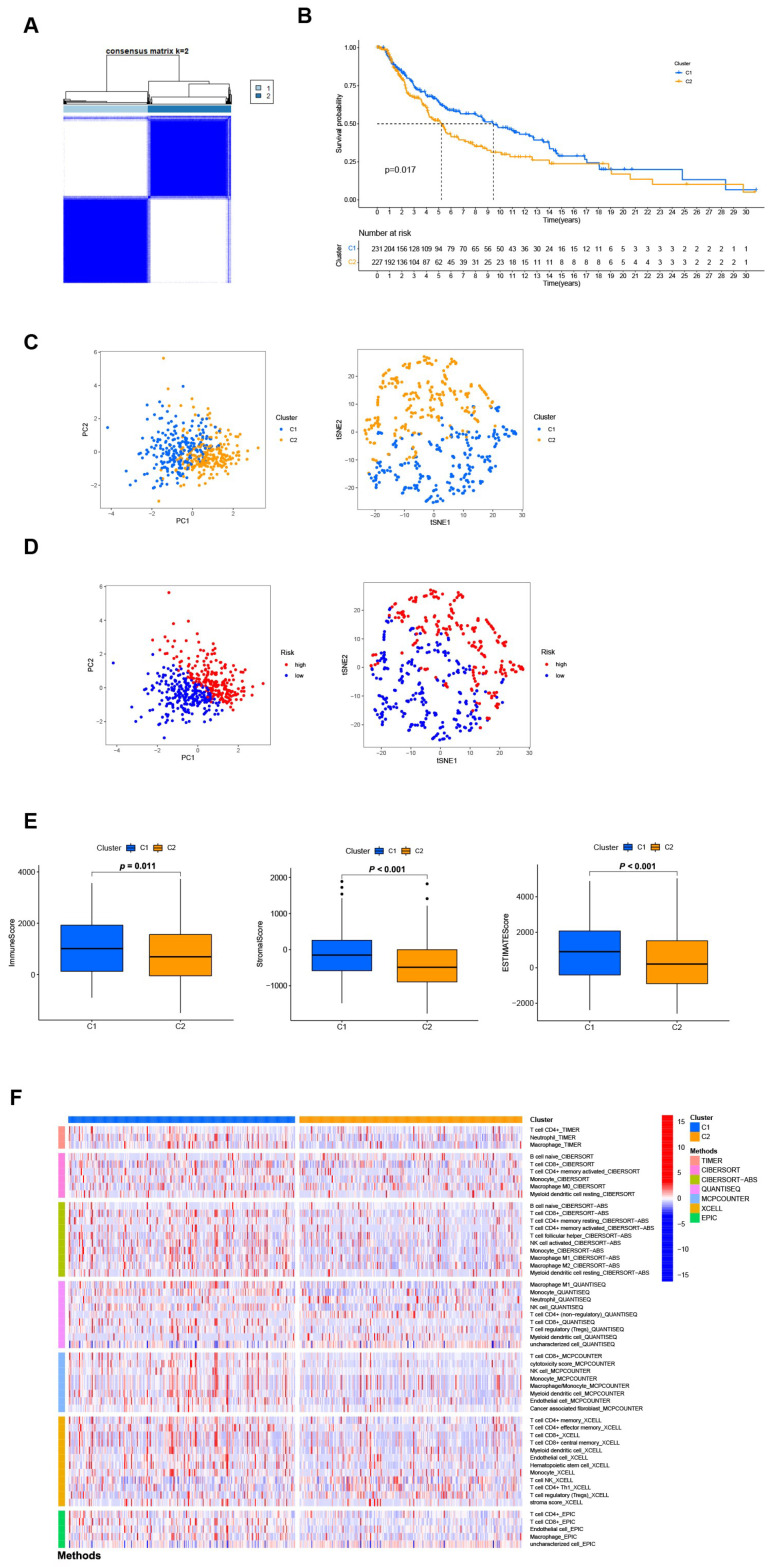
Differentiation and exploration of cold and hot tumors. (**A**) Patients are divided into two clusters by the “ConsensusClusterPlus” R package. (**B**) K-M analyses of OS in different clusters. (**C**,**D**) The t-SNE and PCA analyses in cluster 1 and cluster 2, respectively. (**E**) The comparison of the stromal score, immune score, and ESTIMATE score between clusters 1 and 2, respectively. (**F**) The heat map of immune cells in clusters 1 and 2.

Different clusters (also known as subtypes) may have distinct immune microenvironments, resulting in varying immunotherapy responses [22]. Using the “ConsensusClusterPlus” R package, we regrouped the patients into two clusters based on the expression of four NRGsin the signature (Figure 7A and Table S4). The K-M analysis demonstrated that patients in cluster 2 had a superior OS rate compared to those in cluster 1 (*p* = 0.017, Figure 7B). Besides, t-SNE and PCA analysis showed that the two subgroups could be distinguished with high grouping reliability (Figure 7C,D). Also, cluster 1 had a higher immune score as well as ESTIMATE (microenvironment) score, indicating a different TME from cluster 2 (Figure 7E). A comparison of immune cell infiltration on different platforms revealed a significant difference in immune infiltration between the two clusters (Figure 7F and Table S5). A surprising finding was that nearly all immune checkpoints showed greater activity in cluster 1 (Figure S6). Based on the above results, we could consider cluster 1 as the hot tumor while cluster 2 as the cold tumor. Cluster 1 was more likely to respond to immunotherapy when it was separated into hot tumors. In drug sensitivity comparison, we found 25 immunotherapeutic drugs with different IC50 between different clusters (Figure S7). In the future, we will look at precise drug therapy and immunotherapy responses for SKCM patients.

### 3.8. Verify Signature Gene Expression by IHC

**Figure 8:**
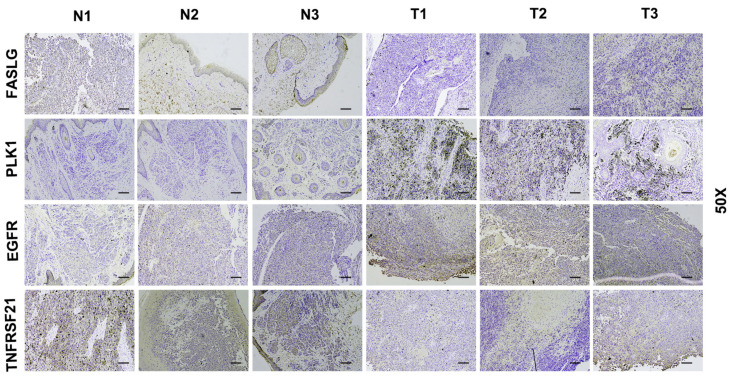
Immunohistochemical staining of 3 normal skin tissues and 3 SKCM samples. IHC results of 3 normal skin tissues and 3 SKCM samples (N1–N3: normal tissues; T1–T3: SKCM tissues). Scale bars: 10 μm.

Immunohistochemical staining was performed to analyze the expression of signature genes in 3 normal skin and 3 SKCM tissues. Due to the differences in model gene coefficients, we consider DR6 and FASLG as negative regulators and PLK1 and EGFR as positive regulators. In Figure 8, we found that immunohistochemical expression was positively or negatively consistent with model gene coefficients.

## 4. Discussion

Previous studies have demonstrated that necroptosis plays a significant role in the progression and metastasis of melanoma [23,24]. Tumor therapy based on targeted necroptosis-related factors is proven to be meaningful [25,26]. Our study analyzed the differential necroptosis genes between melanoma and normal tissue and then constructed a prognostic risk signature. Also, we identified potential LncRNAs associated with necroptosis. We believe that these LncRNAs are involved in regulating the occurrence and development of melanoma by participating in regulating the expression of necroptosis genes, which needs to be confirmed by further experimental studies. After development of the signature, patients in SKCM cohort were regrouped into low- and high-risk groups. Then, K-M analysis, GSEA, and IC_50_ prediction were used to prove the robust reliability of the signature. Moreover, the nomogram results suggest that incorporating the values of the multi-Cox regression model into the calculated risk score can accurately predict the prognostic risk of patients. Although we found that risk groups could guide prognosis and systematic treatments, we were unable to identify the different subtypes. Molecular types and cluster classification of tumors are new and effective means to explore the treatment of tumors [27,28]. In-depth study of the cancer genetic map and its correlation with clinical symptoms and immune characteristics of patients is conducive to accurate diagnosis, prognosis stratification, recurrence monitoring, and drug guidance. Therefore, it is necessary to construct molecular subtypes with different immune and TME scores. Based on the expression of NRGs, we successfully divided the patients into two subtypes. Surprisingly, the two clusters have different immune microenvironments.

Immunotherapy can improve treatment outcomes in cases of failure, but it is not a solution for all diseases [16]. Due to the different tumor immune microenvironments, immunosuppressive and immune activated, the outcomes of patients had a distinct response to immunotherapy. Therefore, to improve the effectiveness of immunotherapy, two different SKCM subtypes were introduced, referring to the immune-based classification of tumors. Tumors with a high immune score were generally considered to be hot tumors, often accompanied by high invasive characteristics, while tumors with a low immune score were generally considered to be cold. In general, hot tumors have a higher response rate to immunotherapy, such as PD-1/PD-L1 treatment, T cell-targeting immunotherapies, and microbiome modulation [29,30]. Also, immune checkpoints are usually expressed at higher levels. And, we found that almost all 25 immune checkpoints were highly expressed in hot tumors (such as LAG3, CD28, and CD80), except for VTCN1, which was only highly expressed in cold tumors. Howerver, Cold tumors have the characteristic of lower tumor mutational burden, poorer antigen presentation, and intrinsic insensitivity to T cell killing [30]. And we noticed that the cold tumors were less sensitive to chemotherapy drugs. This also prompted researchers to pay attention to whether the survival prognosis of patients could be improved by switching patient classification, in addition to the combination of chemotherapy drugs. So, it is extremely meaningful to identify hot and cold tumors in SKCM patients.

In this study, four genes were constructed for our signature (FASLG, PLK1, EGFR, and TNFRSF21). FASLG and TNFRSF21 have a negative coefficient. The coefficients for PLK1 and RGFR are positive. FASLG, also named, FASL, CD95L, is the ligand of FAS. The FAS protein is a cell surface receptor that, when activated by binding to its ligand, FASL, initiates a cascade of signaling events leading to cell death [31]. This FAS/FASL system plays a critical role in the immune system, as well as in the development and progression of certain diseases such as cancer. The study has shown that some organs overexpressed FASL to protect themselves from harmful immune responses [32]. Besides, an in vivo study reported that tumorigenesis was delayed in Fas-deficient LPR mutant mice [33]. TNFRSF21 (tumor-necrosis factor receptor superfamily 21, as known as death receptor 6, DR6) in a lacking animal model showed reduced tumor metastasis capability [34]. Strilic, Strilic B et al. also revealed that the binding of amyloid precursor protein (APP) to DR6 positively regulates the necroptosis pathway [34]. The interaction between APP and DR6 provides a new target for anti-hematogenous tumor metastases [35]. PLK1 (Polo-like kinase 1) is a protein kinase that plays a crucial role in cell division [36]. It is known to be overexpressed in many types of cancer and is a target of several cancer therapeutics in clinical trials. Dysregulation of PLK1 activity can lead to errors in mitosis and contribute to the development of cancer [36]. Analysis of melanoma patients with PLK1 in the TCGA database showed that high mRNA levels were associated with worse survival [37]. Cholewa et al. found that using volasertib (inhibitor of PLK1) induced a significantly delayed melanoma cell growth [38]. EGFR (epidermal growth factor receptor) could be activated by its ligands, leading to undergoing dimerization and phosphorylation, activating multiple downstream carcinogenic signaling pathways [39]. And, a new study showed that activation of the EGFR-STAT3 signaling pathway could be a novel therapeutic approach for melanoma [40]. Above all, these four genes have the significance of predicting the prognostic risk of melanoma patients.

There are also some shortcomings and deficiencies in this study. First, the number of clinical specimens available is limited, and tissue acquisition is difficult. Second, additional prospective evaluation is required for the validation of this approach [41,42,43]. Above all, collecting additional clinical datasets would be our next step to further validate the signature.

Furthermore, both necroptosis-related mRNAs and lncRNAs contribute to the initiation of cell death. Necroptosis can induce cancer cell death in a manner independent of caspase, bypassing apoptosis. LncRNAs usually play a role in regulating apoptosis-related signaling pathways. Studying the potential relationships and mechanisms between these small molecules is beneficial to immunotherapy and tumor research, which provides hypotheses for future basic studies.

## 5. Conclusions

In conclusion, we performed comprehensive and systematic bioinformatics analysis and identified the necroptosis-related prognostic gene signature for SKCM patients, which will make great strides in personalized therapy and improve patient outcomes. The containd regulators were also validated by using immunohistochemistry of clinical samples. Our findings also establish a new classification of cold and hot subtypes for SKCM that plays a crucial role in determining the prognosis of the disease. Further study should be conducted to verify the mechanisms and relationships, among necroptosis, lncRNAs, immunity, and SKCM.

## Figures and Tables

**Table 1:** The coefficients of signature genes.

Gene Name	Coefficient
FASLG	−0.367
PLK1	0.287
EGFR	0.202
TNFRSF21	−0.118

FASLG, Fas ligand; PLK1, Polo like kinase 1; EGFR, Epidermal growth factor receptor; TNFRSF21; TNF receptor superfamily member 21.

## Data Availability

The original contributions presented in the study are included in the article/Supplementary Material, further inquiries can be directed to the corresponding authors.

## Supplementary Materials

The following supporting information can be downloaded at: https://www.mdpi.com/article/10.3390/jpm13020245/s1, Figure S1: The network between necroptosis genes and lncRNAs in SKCM; Figure S2: Kaplan-Meier analysis in clinicopathological features; Figure S3: The ssGSEA analysis between high- and low-risk group; Figure S4: The difference of 42 checkpoints expression; Figure S5: Potential therapeutic medicine of subgroups; Figure S6: The difference of 25 checkpoints expression in different clusters; Figure S7: Potential therapeutic medicine in different clusters; Table S1: 67 necroptosis-related genes; Table S2: The network between necroptosis-related mRNAs and lncRNAs; Table S3: Immune cell bubble chart between high- and low-risk group in multi-platform; Table S4: Different Subtypes in SKCM based on expressions of 4 necroptosis-related genes; Table S5: The heat map of immune cells in clusters.
