# Supplementary material for: Comprehensive Analysis of Necroptosis Landscape in Skin Cutaneous Melanoma for Appealing its Implications in Prognosis Estimation and Microenvironment Status
Source: J Pers Med. 2023 Jan 29;13(2):245. doi: 10.3390/jpm13020245 (PMC9962795; doi:10.3390/jpm13020245)
Supplement: Supplementary file 1 [file jpm-13-00245-s001.zip › Supplement_Figure.pdf]

Identification and Validation of Necroptosis Landscape on Therapy and Prognosis in Skin Cutaneous Melanoma

Supplement_Figure

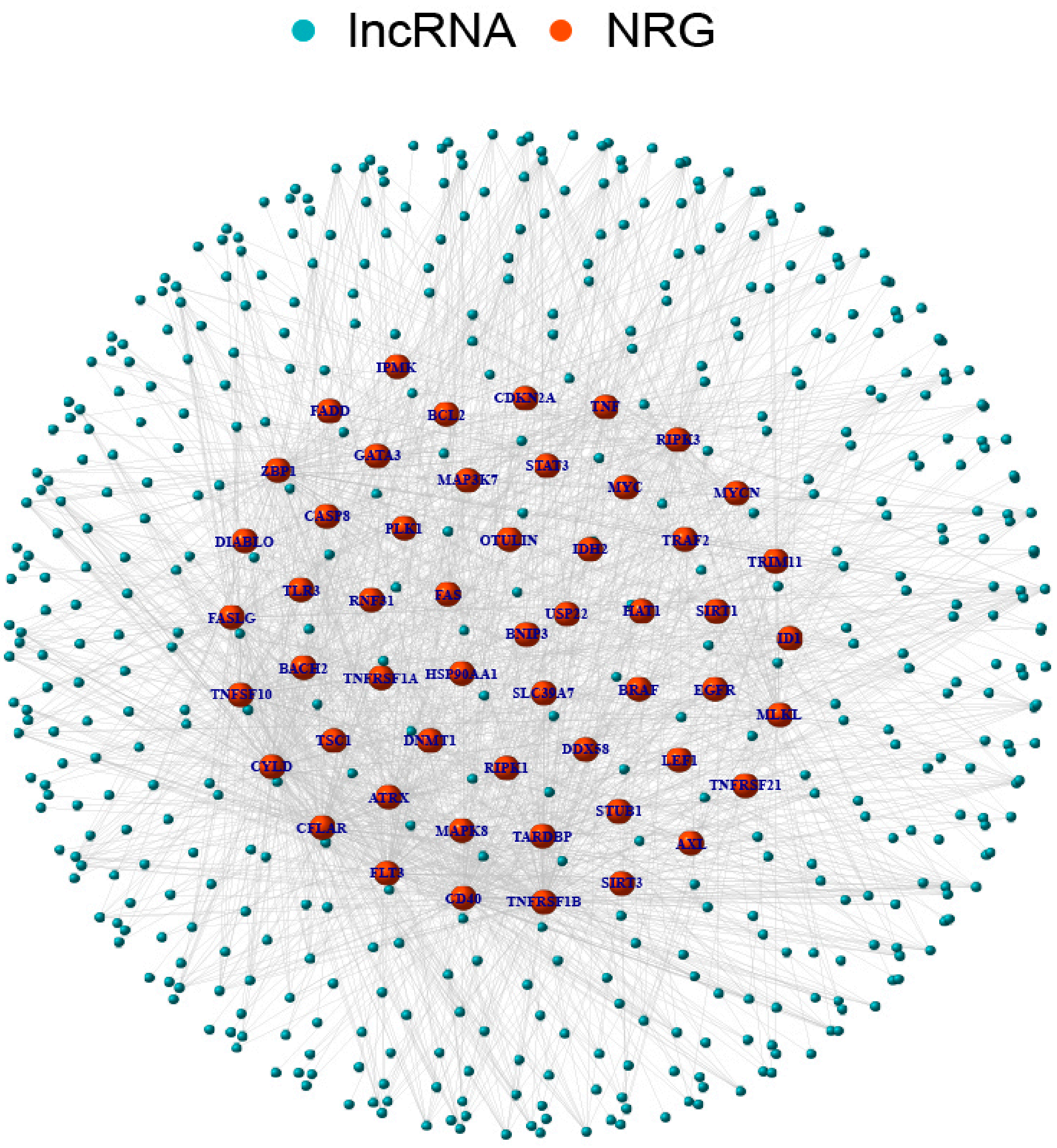

Figure S1 The network between necroptosis genes and lncRNAs in SKCM.
The network between necroptosis genes and lncRNAs in SKCM (green: mRNAs, blue: LncRNAs; correlation coefficients > 0.4 and $p < 0.001$).

Figure S2

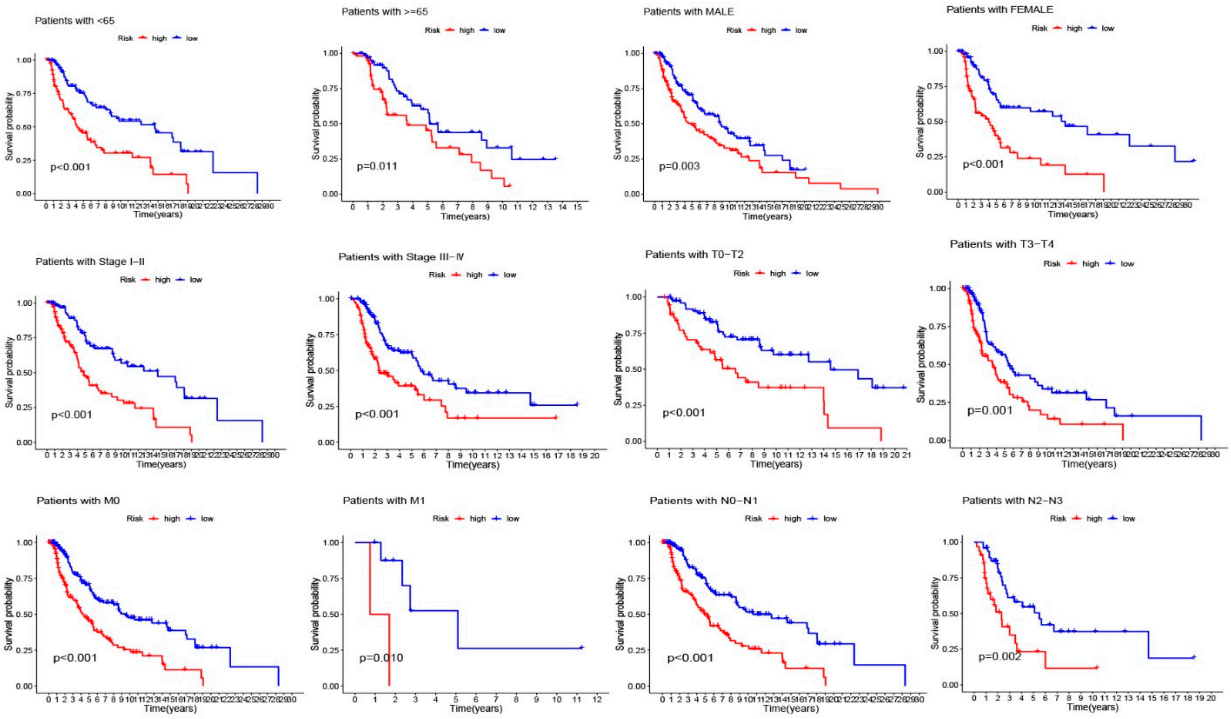

Figure S2 Kaplan-Meier analysis in clinicopathological features.

Kaplan-Meier survival curves of OS prognostic value stratified by age, gender, stage, T, M, or N between low- and high-risk groups in the entire set.

Figure S3

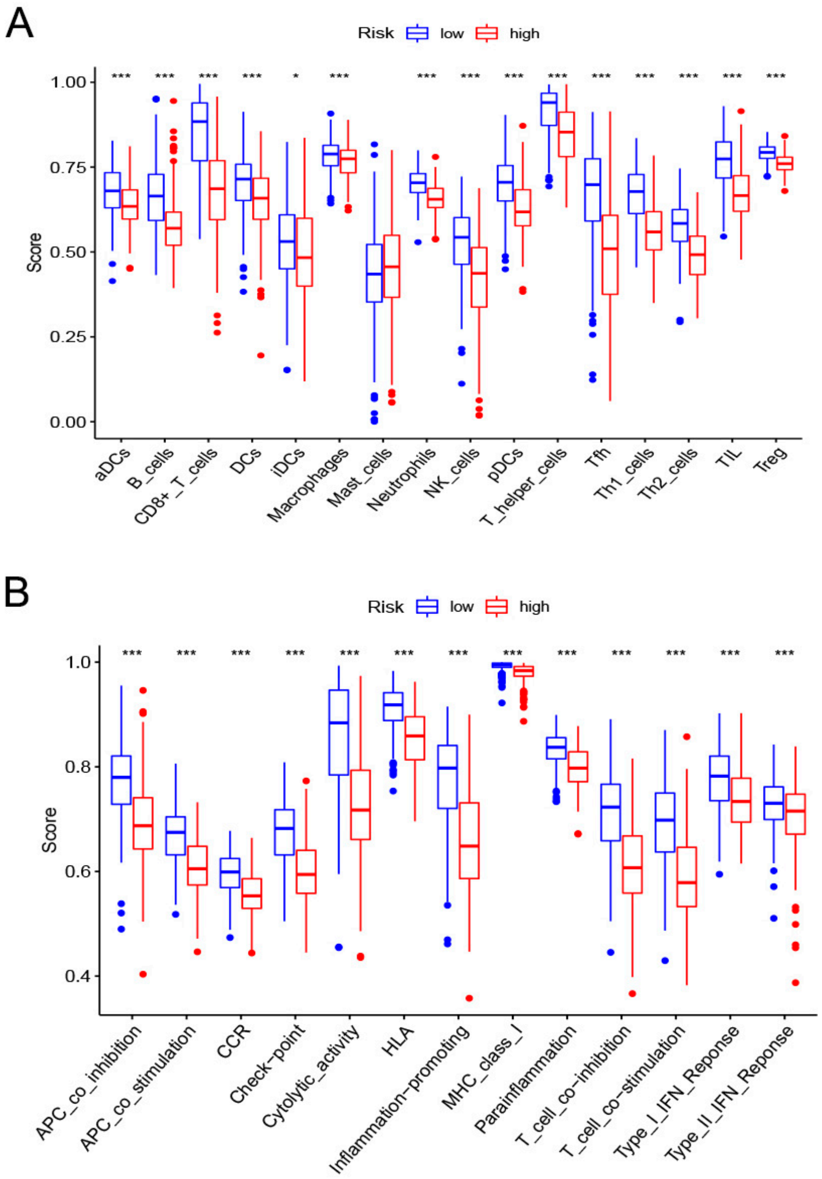

FigureS3 The ssGSEA analysis between high- and low-risk group.
The ssGSEA scores of immune cells and immune functions between high- and low-risk group (*p <0.05, ***p <0.001).

Figure S4

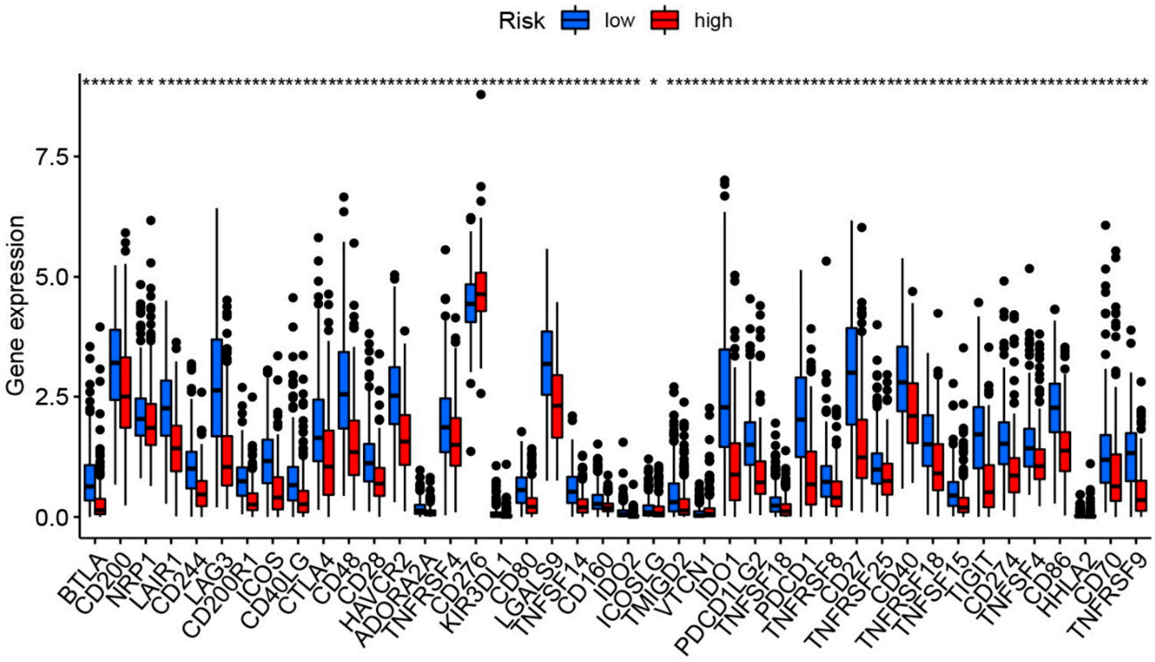

Figure S4 The difference of 42 checkpoints expression.

The difference of 42 checkpoints expression between high- and low-risk group (**p* <0.05, ***p* <0.01, and ****p* <0.001).

Figure S5

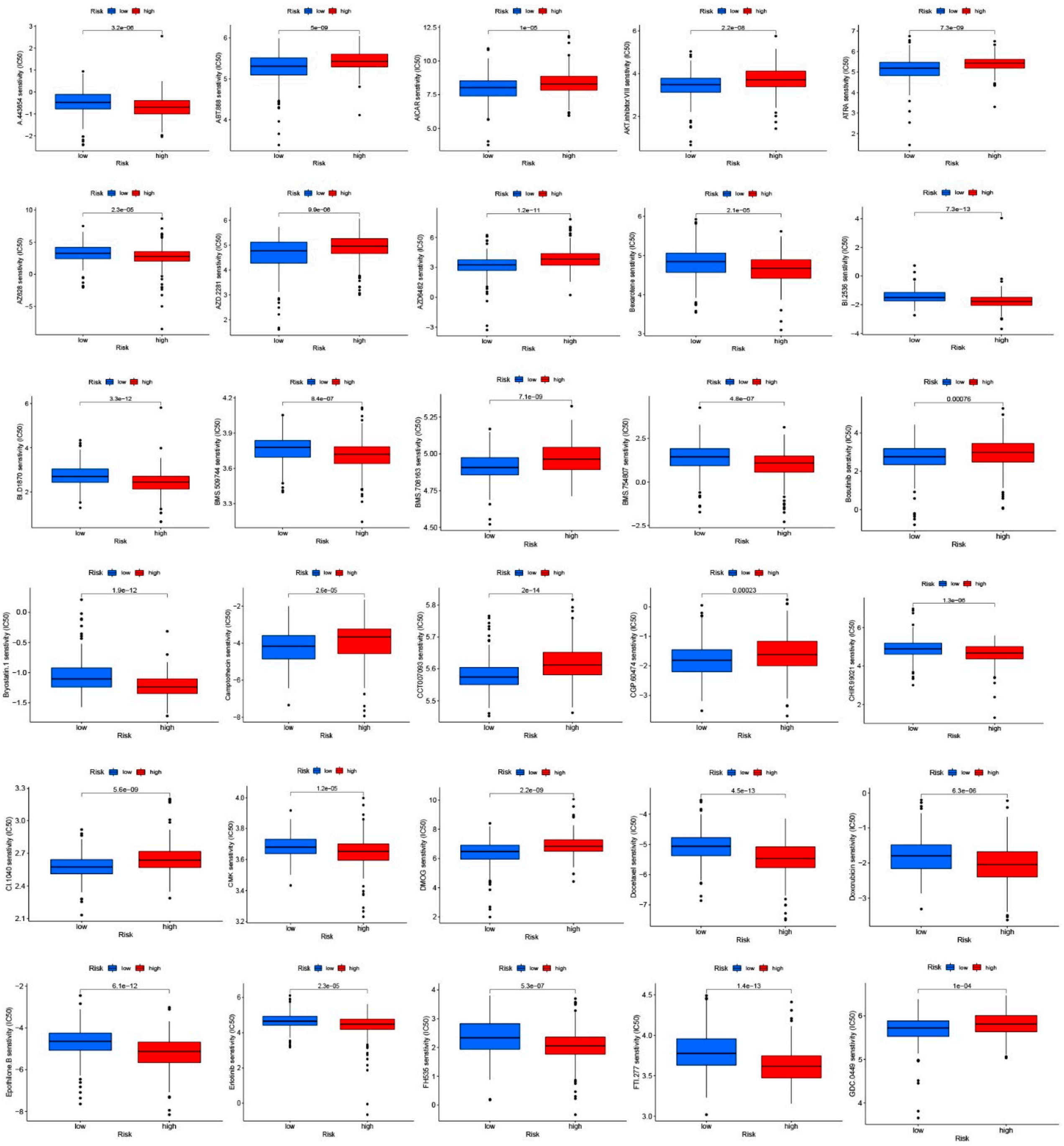
Risk low high
A.443654 sensitivity (IC50)
3.2e-06
ABT.888 sensitivity (IC50)
5e-09
AICAR sensitivity (IC50)
1e-05
AKT.inhibitor.VIII sensitivity (IC50)
2.2e-08
ATRA sensitivity (IC50)
7.3e-09
AZ628 sensitivity (IC50)
2.3e-05
AZD.2281 sensitivity (IC50)
9.9e-08
AZD6482 sensitivity (IC50)
1.2e-11
Bexarotene sensitivity (IC50)
2.1e-05
BI.2536 sensitivity (IC50)
7.3e-13
BIBW2992 sensitivity (IC50)
3.3e-12
BMS.509744 sensitivity (IC50)
8.4e-07
BMS.708163 sensitivity (IC50)
7.1e-09
BMS.754807 sensitivity (IC50)
4.8e-07
Bosutinib sensitivity (IC50)
0.00076
Bryostatin.1 sensitivity (IC50)
1.9e-12
Camptothecin sensitivity (IC50)
2.6e-05
CCT007093 sensitivity (IC50)
2e-14
CGP.60474 sensitivity (IC50)
0.00023
CHIR.99021 sensitivity (IC50)
1.3e-06
CI.1040 sensitivity (IC50)
5.6e-09
CMK sensitivity (IC50)
1.2e-05
DMOG sensitivity (IC50)
2.2e-09
Docetaxel sensitivity (IC50)
4.5e-13
Doxorubicin sensitivity (IC50)
6.3e-06
Epothilone.B sensitivity (IC50)
6.1e-12
Erlotinib sensitivity (IC50)
2.3e-05
FH535 sensitivity (IC50)
5.3e-07
FTI.277 sensitivity (IC50)
1.4e-13
GDC.0449 sensitivity (IC50)
1e-04
low high
Risk

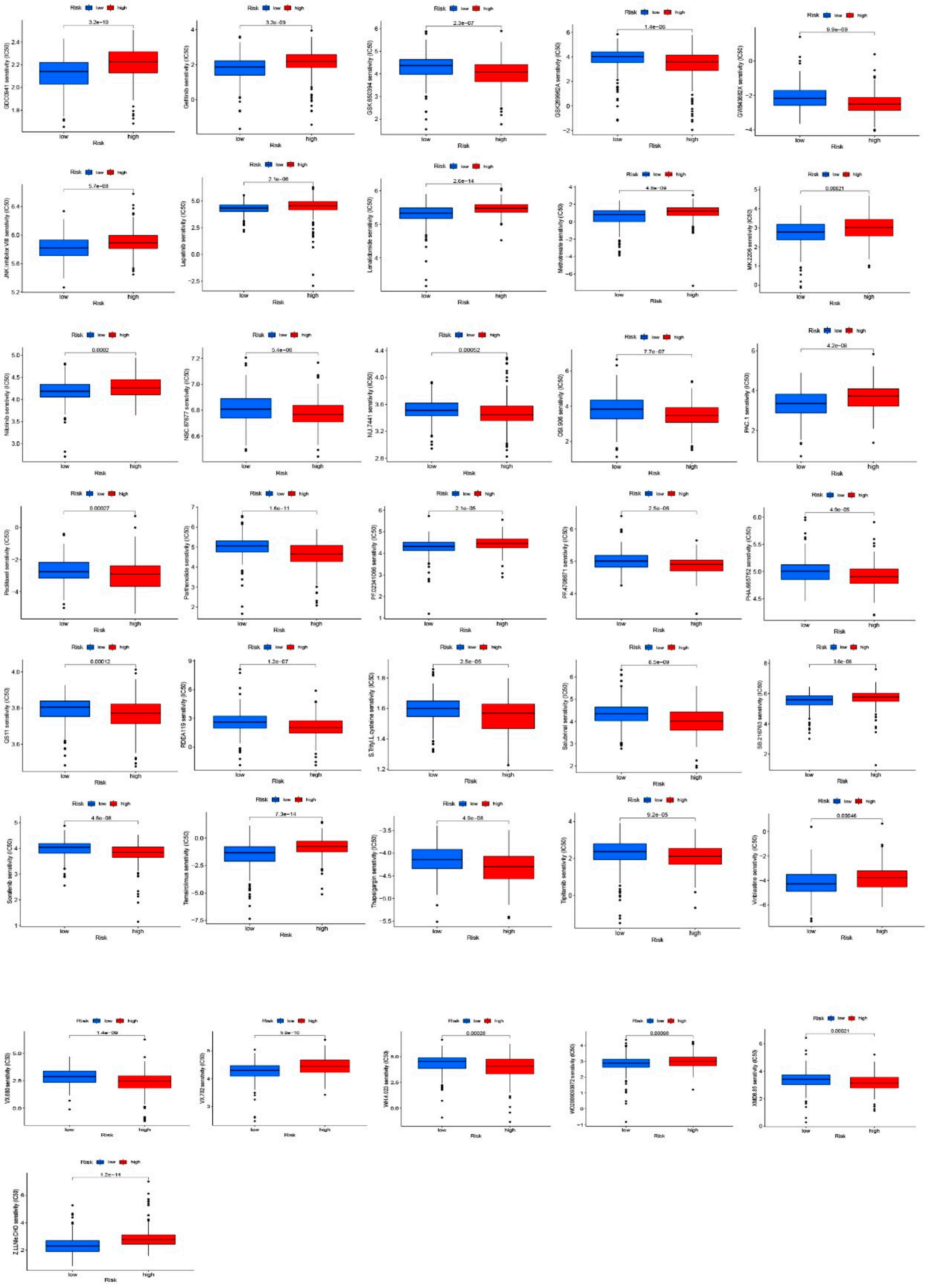

Figure S5 Potential therapeutic medicine of subgroups.
Potential therapeutic medicine of subgroups (all p <0.001).

Figure S6

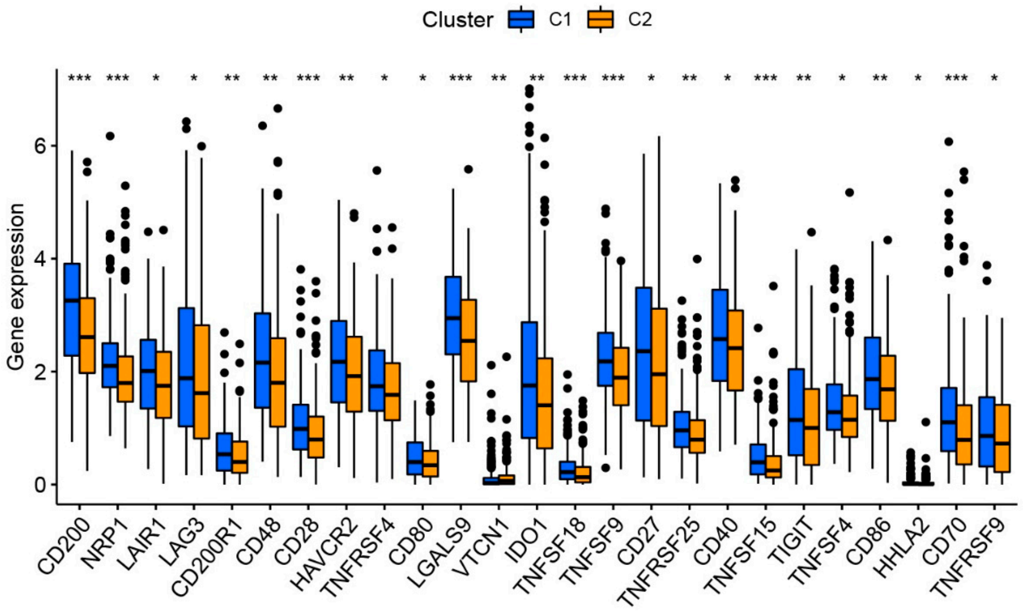

Figure S6 The difference of 25 checkpoints expression in different clusters.
The difference of 25 checkpoints expression in different clusters (**p* <0.05, ***p* <0.01, and ****p* <0.001).

Figure S7

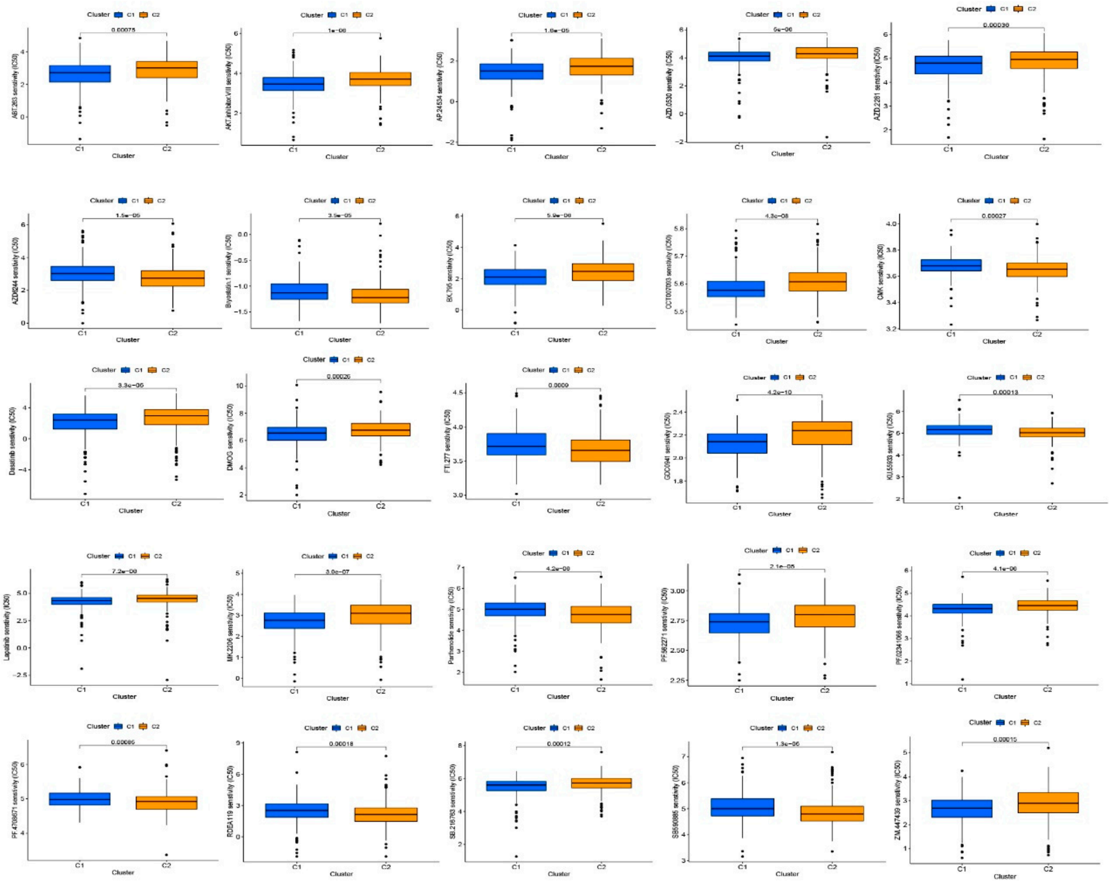

Figure S7 Potential therapeutic medicine in different clusters.
Potential therapeutic medicine in different clusters (all p <0.001).
