# Supplementary material for: Comprehensive Analysis of Necroptosis Landscape in Skin Cutaneous Melanoma for Appealing its Implications in Prognosis Estimation and Microenvironment Status
Source: J Pers Med. 2023 Jan 29;13(2):245. doi: 10.3390/jpm13020245 (PMC9962795; doi:10.3390/jpm13020245)
Supplement: Supplementary file 1 [file jpm-13-00245-s001.zip › Supplement_TableS1.pdf]

Table S1 67 necroptosis-related genes.

Gene
FADD
FAS
FASLG
MLKL
RIPK1
RIPK3
TLR3
TNF
TSC1
TRIM11
CASP8
ZBP1
MAPK8
IPMK
ITPK1
SIRT3
MYC
TNFRSF1A
TNFSF10
TNFRSF1B
TRAF2
PANX1
OTULIN
CYLD
USP22
MAP3K7
SQSTM1
STAT3
DIABLO
DNMT1
CFLAR
BRAF
AXL
ID1
CDKN2A
HSPA4
BCL2
STUB1
FLT3
HAT1
SIRT2
SIRT1

PLK1
MPG
BACH2
GATA3
MYCN
ALK
ATRX
TERT
SLC39A7
SPATA2
RNF31
IDH1
IDH2
KLF9
HDAC9
HSP90AA1
LEF1
BNIP3
CD40
BCL2L11
EGFR
DDX58
TARDBP
APP
TNFRSF21
