# Supplementary material for: Comprehensive Analysis of Necroptosis Landscape in Skin Cutaneous Melanoma for Appealing its Implications in Prognosis Estimation and Microenvironment Status
Source: J Pers Med. 2023 Jan 29;13(2):245. doi: 10.3390/jpm13020245 (PMC9962795; doi:10.3390/jpm13020245)
Supplement: Supplementary file 1 [file jpm-13-00245-s001.zip › Supplement_TableS2.pdf]

Table S2 The network between necroptosis-related mRNAs and lncRNAs.

NRG	lncRNA	cor	pvalue	Regulation
IPMK	ENTPD1-AS1	0.451729	4.62E-25	postive
FADD	ALG1L9P	0.446827	1.70E-24	postive
ZBP1	AC087045.2	0.420914	1.20E-21	postive
DIABLO	ITFG2-AS1	0.41865	2.07E-21	postive
FASLG	HCG27	0.417266	2.88E-21	postive
ZBP1	HCG27	0.490117	7.85E-30	postive
TNFSF10	HCG27	0.440788	8.25E-24	postive
CYLD	HCG27	0.560713	2.37E-40	postive
CFLAR	HCG27	0.518839	8.13E-34	postive
FLT3	HCG27	0.422668	7.82E-22	postive
CD40	HCG27	0.422802	7.57E-22	postive
ZBP1	LINC02384	0.621629	1.06E-51	postive
TNFRSF1B	LINC02384	0.465089	1.18E-26	postive
CYLD	LINC02384	0.430638	1.09E-22	postive
CFLAR	LINC02384	0.412035	9.96E-21	postive
FLT3	LINC02384	0.443069	4.56E-24	postive
CD40	LINC02384	0.498357	6.17E-31	postive
SIRT3	TOLLIP-AS1	0.507059	3.89E-32	postive
AXL	AL158166.1	0.424854	4.58E-22	postive
TNFRSF21	MIR100HG	0.406835	3.35E-20	postive
FASLG	AL022316.1	0.45739	9.96E-26	postive
MLKL	AL022316.1	0.430768	1.06E-22	postive
ZBP1	AL022316.1	0.576395	4.77E-43	postive
TNFSF10	AL022316.1	0.425066	4.35E-22	postive
TNFRSF1B	AL022316.1	0.5569	1.02E-39	postive
CYLD	AL022316.1	0.442149	5.80E-24	postive
CFLAR	AL022316.1	0.407237	3.05E-20	postive
FLT3	AL022316.1	0.470278	2.72E-27	postive
CD40	AL022316.1	0.5091	2.01E-32	postive
SIRT3	AP006621.3	0.437961	1.71E-23	postive
ID1	LINC02432	0.50707	3.88E-32	postive
TRIM11	AL121906.2	0.416378	3.56E-21	postive
FASLG	LINC02195	0.434303	4.34E-23	postive
MYCN	MYCNOS	0.53938	6.58E-37	postive
TRIM11	AC008608.2	0.473204	1.18E-27	postive
FASLG	AC004847.1	0.568905	9.64E-42	postive
MLKL	AC004847.1	0.483873	5.16E-29	postive
RIPK3	AC004847.1	0.577812	2.68E-43	postive
TNF	AC004847.1	0.582059	4.66E-44	postive
ZBP1	AC004847.1	0.646785	3.72E-57	postive
TNFSF10	AC004847.1	0.496416	1.13E-30	postive
TNFRSF1B	AC004847.1	0.737998	3.81E-82	postive

CYLD	AC004847.1	0.54892	2.04E-38	postive
CFLAR	AC004847.1	0.55833	5.91E-40	postive
FLT3	AC004847.1	0.704103	9.68E-72	postive
CD40	AC004847.1	0.637189	5.15E-55	postive
CDKN2A	CDKN2A-DT	0.727526	9.19E-79	postive
ZBP1	AL133467.1	0.707378	1.11E-72	postive
TNFRSF1B	AL133467.1	0.446299	1.96E-24	postive
FLT3	AL133467.1	0.477799	3.10E-28	postive
BCL2	AC027130.1	0.412812	8.30E-21	postive
GATA3	AP001207.3	0.539244	6.91E-37	postive
FASLG	AC133644.1	0.628092	4.71E-53	postive
MLKL	AC133644.1	0.536097	2.12E-36	postive
RIPK3	AC133644.1	0.403563	7.10E-20	postive
TNF	AC133644.1	0.418989	1.91E-21	postive
CASP8	AC133644.1	0.426208	3.28E-22	postive
ZBP1	AC133644.1	0.732138	3.12E-80	postive
TNFSF10	AC133644.1	0.580646	8.36E-44	postive
TNFRSF1B	AC133644.1	0.697027	9.40E-70	postive
CYLD	AC133644.1	0.661086	1.71E-60	postive
CFLAR	AC133644.1	0.629223	2.71E-53	postive
FLT3	AC133644.1	0.622556	6.83E-52	postive
CD40	AC133644.1	0.638626	2.49E-55	postive
TNFRSF1B	AC015819.2	0.426504	3.05E-22	postive
TLR3	AC022034.1	0.400977	1.28E-19	postive
FASLG	USP30-AS1	0.833494	######	postive
MLKL	USP30-AS1	0.599192	3.09E-47	postive
RIPK3	USP30-AS1	0.495268	1.61E-30	postive
TNF	USP30-AS1	0.457634	9.32E-26	postive
ZBP1	USP30-AS1	0.791589	######	postive
TNFSF10	USP30-AS1	0.607484	7.62E-49	postive
TNFRSF1B	USP30-AS1	0.779146	3.48E-97	postive
CYLD	USP30-AS1	0.564267	5.97E-41	postive
CFLAR	USP30-AS1	0.618498	4.69E-51	postive
FLT3	USP30-AS1	0.508955	2.11E-32	postive
CD40	USP30-AS1	0.580596	8.54E-44	postive
FLT3	AC002546.1	0.586061	8.77E-45	postive
CD40	AC002546.1	0.405923	4.13E-20	postive
BACH2	AL358332.1	0.452091	4.19E-25	postive
GATA3	AL358332.1	0.793837	######	postive
MYCN	AL358332.1	0.641757	5.04E-56	postive
TSC1	AL355574.1	0.434842	3.79E-23	postive
FASLG	U62317.3	0.584189	1.92E-44	postive
MLKL	U62317.3	0.520727	4.31E-34	postive
ZBP1	U62317.3	0.677908	1.16E-64	postive

TNFSF10	U62317.3	0.502898	1.47E-31	postive
TNFRSF1B	U62317.3	0.530658	1.44E-35	postive
CYLD	U62317.3	0.439468	1.16E-23	postive
CFLAR	U62317.3	0.456412	1.30E-25	postive
CD40	U62317.3	0.44056	8.75E-24	postive
ATRX	CIRBP-AS1	-0.41093	1.29E-20	negative
GATA3	AC016877.3	0.428956	1.66E-22	postive
ID1	HOXC13-AS	0.404893	5.24E-20	postive
GATA3	HOXC13-AS	0.537004	1.54E-36	postive
MAPK8	AL450384.2	0.424764	4.68E-22	postive
TARDBP	AL450384.2	0.445341	2.52E-24	postive
AXL	BX640514.2	0.41882	1.98E-21	postive
GATA3	AL354950.1	0.551331	8.31E-39	postive
MYCN	AL354950.1	0.59724	7.28E-47	postive
FASLG	AL357054.4	0.409606	1.76E-20	postive
FASLG	AC004921.1	0.598681	3.87E-47	postive
MLKL	AC004921.1	0.444163	3.43E-24	postive
RIPK3	AC004921.1	0.473903	9.62E-28	postive
ZBP1	AC004921.1	0.561598	1.68E-40	postive
TNFRSF1B	AC004921.1	0.638383	2.81E-55	postive
CD40	AC004921.1	0.434319	4.32E-23	postive
TRIM11	AC114271.1	0.460111	4.72E-26	postive
STUB1	AC114271.1	0.423941	5.73E-22	postive
ATRX	AC114271.1	-0.41568	4.21E-21	negative
TARDBP	AC114271.1	-0.41978	1.58E-21	negative
DIABLO	AL354928.1	0.433939	4.76E-23	postive
TARDBP	AL050341.2	0.468953	3.97E-27	postive
TRIM11	TFAP2A-AS1	0.420492	1.33E-21	postive
TNF	LINC01215	0.451391	5.05E-25	postive
ZBP1	LINC01215	0.426603	2.98E-22	postive
TNFRSF1B	LINC01215	0.429089	1.61E-22	postive
CYLD	LINC01215	0.577534	3.00E-43	postive
CFLAR	LINC01215	0.480754	1.30E-28	postive
FLT3	LINC01215	0.688731	1.70E-67	postive
BACH2	LINC01215	0.507412	3.48E-32	postive
CD40	LINC01215	0.60131	1.21E-47	postive
BACH2	SLC25A25-AS1	0.424916	4.51E-22	postive
CYLD	PIK3CD-AS2	-0.42781	2.21E-22	negative
DIABLO	ST20-AS1	0.453962	2.53E-25	postive
LEF1	LEF1-AS1	0.689868	8.43E-68	postive
DIABLO	WWTR1-AS1	0.42351	6.37E-22	postive
TRIM11	AC006942.1	0.438641	1.44E-23	postive
GATA3	LINC01836	0.443341	4.25E-24	postive

EGFR	LINC01836	0.48115	1.16E-28	postive
TNFSF10	AC104083.1	0.449607	8.14E-25	postive
TNFRSF1B	AC104083.1	0.429537	1.44E-22	postive
CYLD	AC104083.1	0.461084	3.61E-26	postive
FLT3	AC104083.1	0.452322	3.94E-25	postive
TRIM11	AL163051.1	0.443917	3.66E-24	postive
MAPK8	AC007390.1	0.498548	5.81E-31	postive
IPMK	AC007390.1	0.401551	1.12E-19	postive
SIRT1	AC007390.1	0.430398	1.16E-22	postive
ATRX	AC007390.1	0.425602	3.81E-22	postive
TARDBP	AC007390.1	0.469918	3.02E-27	postive
TNFRSF1B	AC245128.3	0.429069	1.62E-22	postive
TRAF2	HHLA3	0.418624	2.08E-21	postive
FASLG	AL158071.2	0.804881	######	postive
MLKL	AL158071.2	0.460517	4.22E-26	postive
ZBP1	AL158071.2	0.535149	2.97E-36	postive
TNFSF10	AL158071.2	0.529727	1.99E-35	postive
TNFRSF1B	AL158071.2	0.596652	9.41E-47	postive
CYLD	AL158071.2	0.445699	2.29E-24	postive
CFLAR	AL158071.2	0.489049	1.09E-29	postive
MYC	AC107027.3	0.426483	3.07E-22	postive
GATA3	AC092171.3	0.637792	3.80E-55	postive
MYCN	AC092171.3	0.467452	6.07E-27	postive
TARDBP	AC080112.1	-0.48955	9.34E-30	negative
TRAF2	LAMC1-AS1	-0.40132	1.18E-19	negative
STAT3	SNHG17	-0.40114	1.23E-19	negative
BCL2	AL121820.1	0.437215	2.07E-23	postive
FASLG	AC022182.2	0.411396	1.16E-20	postive
ZBP1	AC022182.2	0.570707	4.71E-42	postive
TNFRSF1B	AC022182.2	0.53001	1.80E-35	postive
CYLD	AC022182.2	0.46599	9.17E-27	postive
CFLAR	AC022182.2	0.423312	6.68E-22	postive
FLT3	AC022182.2	0.518349	9.58E-34	postive
CD40	AC022182.2	0.546615	4.76E-38	postive
TNF	AC109446.2	0.465082	1.18E-26	postive
ZBP1	AC109446.2	0.578049	2.43E-43	postive
TNFSF10	AC109446.2	0.404526	5.70E-20	postive
TNFRSF1B	AC109446.2	0.537574	1.26E-36	postive
CYLD	AC109446.2	0.521013	3.92E-34	postive
CFLAR	AC109446.2	0.511661	8.75E-33	postive
FLT3	AC109446.2	0.609351	3.26E-49	postive
CD40	AC109446.2	0.627997	4.93E-53	postive
MAPK8	AC018690.1	0.438923	1.34E-23	postive
MAP3K7	AC018690.1	0.421784	9.69E-22	postive

TARDBP	AC018690.1	0.428712	1.77E-22	postive
MLKL	LINC02273	0.429403	1.49E-22	postive
RIPK3	LINC02273	0.458314	7.74E-26	postive
TNF	LINC02273	0.552896	4.62E-39	postive
ZBP1	LINC02273	0.562413	1.23E-40	postive
TNFSF10	LINC02273	0.458707	6.94E-26	postive
TNFRSF1B	LINC02273	0.60076	1.55E-47	postive
CYLD	LINC02273	0.653218	1.23E-58	postive
CFLAR	LINC02273	0.568784	1.01E-41	postive
FLT3	LINC02273	0.810538	######	postive
BACH2	LINC02273	0.419773	1.58E-21	postive
CD40	LINC02273	0.651021	3.99E-58	postive
CASP8	AC018653.3	0.402542	8.96E-20	postive
SIRT1	AC016866.1	0.431757	8.25E-23	postive
TRIM11	A1BG-AS1	0.459139	6.17E-26	postive
FASLG	AC138207.5	0.677043	1.92E-64	postive
MLKL	AC138207.5	0.455351	1.74E-25	postive
RIPK3	AC138207.5	0.491821	4.67E-30	postive
ZBP1	AC138207.5	0.492671	3.60E-30	postive
TNFSF10	AC138207.5	0.600381	1.83E-47	postive
TNFRSF1B	AC138207.5	0.68748	3.66E-67	postive
CYLD	AC138207.5	0.445838	2.21E-24	postive
CFLAR	AC138207.5	0.489803	8.64E-30	postive
CD40	AC138207.5	0.40276	8.53E-20	postive
MAPK8	AL157392.3	0.52487	1.06E-34	postive
EGFR	AC114956.1	0.665488	1.47E-61	postive
MLKL	LINC01550	0.448243	1.17E-24	postive
RIPK3	LINC01550	0.430406	1.16E-22	postive
TNF	LINC01550	0.444154	3.44E-24	postive
ZBP1	LINC01550	0.406912	3.29E-20	postive
TNFSF10	LINC01550	0.408299	2.39E-20	postive
TNFRSF1B	LINC01550	0.520077	5.37E-34	postive
CYLD	LINC01550	0.478084	2.85E-28	postive
CFLAR	LINC01550	0.45627	1.35E-25	postive
FLT3	LINC01550	0.613988	3.86E-50	postive
CD40	LINC01550	0.44851	1.09E-24	postive
FASLG	AC006033.2	0.729337	2.45E-79	postive
MLKL	AC006033.2	0.508948	2.12E-32	postive
RIPK3	AC006033.2	0.454787	2.02E-25	postive
TNF	AC006033.2	0.405766	4.28E-20	postive
ZBP1	AC006033.2	0.595762	1.39E-46	postive
TNFSF10	AC006033.2	0.608153	5.63E-49	postive
TNFRSF1B	AC006033.2	0.765921	5.15E-92	postive
CYLD	AC006033.2	0.606911	9.88E-49	postive

CFLAR	AC006033.2	0.574337	1.10E-42	postive
FLT3	AC006033.2	0.558067	6.53E-40	postive
CD40	AC006033.2	0.503661	1.16E-31	postive
TRAF2	AL441992.1	0.482912	6.87E-29	postive
DIABLO	TMPO-AS1	0.439485	1.16E-23	postive
PLK1	TMPO-AS1	0.566354	2.64E-41	postive
ZBP1	AL512306.2	0.458394	7.57E-26	postive
TNFRSF1B	AL512306.2	0.415618	4.27E-21	postive
CYLD	AL512306.2	0.487494	1.74E-29	postive
CFLAR	AL512306.2	0.465746	9.82E-27	postive
FLT3	AL512306.2	0.489249	1.02E-29	postive
CD40	AL512306.2	0.562074	1.40E-40	postive
MAPK8	LINC00641	0.409884	1.65E-20	postive
RNF31	AC092295.2	0.459854	5.07E-26	postive
TNFRSF1B	LINC01150	0.475977	5.28E-28	postive
ID1	GATA3-AS1	0.54826	2.60E-38	postive
BACH2	GATA3-AS1	0.470691	2.42E-27	postive
GATA3	GATA3-AS1	0.852319	######	postive
MYCN	GATA3-AS1	0.675602	4.48E-64	postive
ATRX	AC114956.2	0.412094	9.83E-21	postive
SIRT1	AC009171.2	-0.4171	3.00E-21	negative
RIPK3	AP003071.4	0.464127	1.55E-26	postive
MLKL	LINC01506	0.430094	1.25E-22	postive
TNFRSF1B	LINC01506	0.41745	2.76E-21	postive
MAPK8	AC005034.5	0.433855	4.86E-23	postive
ATRX	AC005034.5	0.452645	3.61E-25	postive
TNF	LINC01781	0.517942	1.10E-33	postive
ZBP1	LINC01781	0.440555	8.77E-24	postive
TNFRSF1B	LINC01781	0.401935	1.03E-19	postive
CYLD	LINC01781	0.490584	6.81E-30	postive
CFLAR	LINC01781	0.408415	2.32E-20	postive
FLT3	LINC01781	0.638224	3.05E-55	postive
BACH2	LINC01781	0.42299	7.23E-22	postive
CD40	LINC01781	0.555265	1.89E-39	postive
ZBP1	HCFC1-AS1	0.519246	7.09E-34	postive
TNFRSF1B	HCFC1-AS1	0.441238	7.34E-24	postive
TRIM11	AL117332.1	0.411832	1.05E-20	postive
FASLG	PCED1B-AS1	0.607351	8.09E-49	postive
MLKL	PCED1B-AS1	0.563566	7.84E-41	postive
RIPK3	PCED1B-AS1	0.55105	9.23E-39	postive
TNF	PCED1B-AS1	0.608208	5.49E-49	postive
CASP8	PCED1B-AS1	0.418058	2.38E-21	postive
ZBP1	PCED1B-AS1	0.776846	2.92E-96	postive
TNFSF10	PCED1B-AS1	0.583945	2.13E-44	postive

TNFRSF1B	PCED1B-AS1	0.775414	1.09E-95	postive
CYLD	PCED1B-AS1	0.712884	2.73E-74	postive
CFLAR	PCED1B-AS1	0.672062	3.50E-63	postive
FLT3	PCED1B-AS1	0.792863	######	postive
CD40	PCED1B-AS1	0.753904	1.32E-87	postive
SIRT3	AP001318.2	0.418032	2.40E-21	postive
CYLD	AC093726.1	0.407014	3.21E-20	postive
TARDBP	AL031714.1	0.403563	7.10E-20	postive
SIRT1	AC005288.1	0.440445	9.02E-24	postive
ATRX	AC005288.1	0.447684	1.36E-24	postive
BCL2	AC027097.1	0.437155	2.10E-23	postive
SIRT3	AC100810.1	0.406787	3.39E-20	postive
EGFR	AC080038.1	0.518017	1.07E-33	postive
GATA3	LINC00885	0.688305	2.21E-67	postive
GATA3	AC245041.1	0.700796	8.35E-71	postive
FASLG	XXYLT1-AS2	0.543405	1.54E-37	postive
ZBP1	XXYLT1-AS2	0.554525	2.51E-39	postive
TNFRSF1B	XXYLT1-AS2	0.516088	2.03E-33	postive
FLT3	XXYLT1-AS2	0.45995	4.93E-26	postive
CD40	XXYLT1-AS2	0.408359	2.35E-20	postive
AXL	LINC00973	0.480301	1.49E-28	postive
AXL	AC093673.1	0.466918	7.06E-27	postive
TRAF2	NORAD	-0.44231	5.56E-24	negative
ATRX	NORAD	0.550354	1.20E-38	postive
TRIM11	AC138696.2	0.452848	3.42E-25	postive
TRAF2	AC138696.2	0.433888	4.82E-23	postive
ATRX	AC138696.2	-0.41669	3.31E-21	negative
TRAF2	ARRDC1-AS1	0.618656	4.35E-51	postive
ATRX	ARRDC1-AS1	-0.45012	7.09E-25	negative
TNFRSF1B	LINC00649	0.401983	1.02E-19	postive
CYLD	LINC00649	0.409501	1.80E-20	postive
CFLAR	LINC00649	0.413319	7.36E-21	postive
TRAF2	U62317.1	0.467103	6.70E-27	postive
TARDBP	AP000866.6	0.453885	2.58E-25	postive
FASLG	AC004687.1	0.45301	3.27E-25	postive
MLKL	AC004687.1	0.448511	1.09E-24	postive
RIPK3	AC004687.1	0.452786	3.47E-25	postive
TNF	AC004687.1	0.628193	4.48E-53	postive
ZBP1	AC004687.1	0.595517	1.54E-46	postive
TNFSF10	AC004687.1	0.455232	1.79E-25	postive
TNFRSF1B	AC004687.1	0.565198	4.15E-41	postive
CYLD	AC004687.1	0.591555	8.52E-46	postive
CFLAR	AC004687.1	0.564798	4.85E-41	postive
FLT3	AC004687.1	0.691133	3.84E-68	postive

CD40	AC004687.1	0.630925	1.17E-53	postive
MAP3K7	AL133338.1	0.491042	5.92E-30	postive
FASLG	TRBV11-2	0.65128	3.48E-58	postive
MLKL	TRBV11-2	0.468945	3.98E-27	postive
RIPK3	TRBV11-2	0.496802	1.00E-30	postive
TNF	TRBV11-2	0.470126	2.84E-27	postive
ZBP1	TRBV11-2	0.65366	9.74E-59	postive
TNFSF10	TRBV11-2	0.462907	2.17E-26	postive
TNFRSF1B	TRBV11-2	0.736762	9.75E-82	postive
CYLD	TRBV11-2	0.541348	3.24E-37	postive
CFLAR	TRBV11-2	0.529491	2.15E-35	postive
FLT3	TRBV11-2	0.637607	4.17E-55	postive
CD40	TRBV11-2	0.557685	7.55E-40	postive
MAPK8	FTX	0.405021	5.09E-20	postive
SIRT1	FTX	0.475035	6.93E-28	postive
ATRX	FTX	0.569326	8.15E-42	postive
CYLD	AC025279.1	0.42112	1.14E-21	postive
FLT3	AC025279.1	0.60656	1.16E-48	postive
BACH2	AC025279.1	0.486394	2.42E-29	postive
CD40	AC025279.1	0.45499	1.91E-25	postive
RIPK3	AC007728.3	0.421138	1.13E-21	postive
FLT3	AC007998.3	0.550534	1.12E-38	postive
SIRT3	NAPA-AS1	0.404442	5.81E-20	postive
MAP3K7	NAPA-AS1	-0.40716	3.11E-20	negative
TARDBP	N4BP2L2-IT2	0.476832	4.11E-28	postive
ATRX	AC024075.1	0.469033	3.88E-27	postive
TNFRSF1A	LINC00941	0.495945	1.31E-30	postive
AXL	LINC00941	0.491202	5.64E-30	postive
TARDBP	AL355488.1	0.52175	3.05E-34	postive
FASLG	AL590764.1	0.698536	3.58E-70	postive
MLKL	AL590764.1	0.591666	8.12E-46	postive
RIPK3	AL590764.1	0.56508	4.34E-41	postive
TNF	AL590764.1	0.552742	4.90E-39	postive
CASP8	AL590764.1	0.419538	1.67E-21	postive
ZBP1	AL590764.1	0.784455	2.31E-99	postive
TNFSF10	AL590764.1	0.615121	2.28E-50	postive
TNFRSF1B	AL590764.1	0.810458	######	postive
CYLD	AL590764.1	0.690043	7.56E-68	postive
CFLAR	AL590764.1	0.6653	1.64E-61	postive
FLT3	AL590764.1	0.757423	7.19E-89	postive
CD40	AL590764.1	0.706621	1.84E-72	postive
ZBP1	AC244205.1	0.631132	1.06E-53	postive
FASLG	AL162414.1	0.804855	######	postive
MLKL	AL162414.1	0.544886	8.97E-38	postive

ZBP1	AL162414.1	0.623046	5.40E-52	postive
TNFSF10	AL162414.1	0.643783	1.77E-56	postive
TNFRSF1B	AL162414.1	0.746766	4.19E-85	postive
CYLD	AL162414.1	0.577087	3.60E-43	postive
CFLAR	AL162414.1	0.585848	9.59E-45	postive
FLT3	AL162414.1	0.441259	7.30E-24	postive
CD40	AL162414.1	0.504931	7.71E-32	postive
RNF31	AL604028.1	-0.48082	1.28E-28	negative
TNF	AL844908.1	0.457532	9.58E-26	postive
ZBP1	AL844908.1	0.456065	1.43E-25	postive
CYLD	AL844908.1	0.426199	3.29E-22	postive
CFLAR	AL844908.1	0.409875	1.65E-20	postive
FLT3	AL844908.1	0.558908	4.73E-40	postive
CD40	AL844908.1	0.492962	3.29E-30	postive
MAPK8	AC022400.4	0.454714	2.06E-25	postive
IPMK	AC022400.4	0.496469	1.11E-30	postive
SIRT1	AC022400.4	0.4783	2.68E-28	postive
DNMT1	AL161891.1	0.485442	3.22E-29	postive
SIRT1	AL731563.3	0.404245	6.08E-20	postive
MLKL	PRKCQ-AS1	0.408609	2.22E-20	postive
TNF	PRKCQ-AS1	0.434515	4.11E-23	postive
ZBP1	PRKCQ-AS1	0.472203	1.57E-27	postive
TNFRSF1B	PRKCQ-AS1	0.494031	2.37E-30	postive
CYLD	PRKCQ-AS1	0.486693	2.21E-29	postive
CFLAR	PRKCQ-AS1	0.428354	1.93E-22	postive
FLT3	PRKCQ-AS1	0.590356	1.42E-45	postive
CD40	PRKCQ-AS1	0.519412	6.71E-34	postive
FASLG	AC005082.1	0.412561	8.80E-21	postive
ZBP1	AC005082.1	0.438023	1.68E-23	postive
TNFRSF1B	AC005082.1	0.55014	1.29E-38	postive
FADD	AC012640.1	0.407495	2.87E-20	postive
TARDBP	AC005104.1	0.535709	2.44E-36	postive
SIRT3	AL355001.2	0.434416	4.22E-23	postive
FASLG	AC010175.1	0.442784	4.91E-24	postive
TNFRSF1B	AC010175.1	0.402955	8.16E-20	postive
CYLD	AC010175.1	0.432814	6.32E-23	postive
FASLG	AP002954.1	0.550915	9.70E-39	postive
RIPK3	AP002954.1	0.494878	1.82E-30	postive
ZBP1	AP002954.1	0.483317	6.09E-29	postive
TNFRSF1B	AP002954.1	0.637584	4.22E-55	postive
RIPK1	GMDS-DT	0.410853	1.31E-20	postive
MLKL	AC010327.4	0.418343	2.23E-21	postive
AXL	AC010327.4	0.479397	1.94E-28	postive
RIPK3	LINC01503	0.418975	1.91E-21	postive

FASLG	AC099343.2	0.562616	1.13E-40	postive
MLKL	AC099343.2	0.433099	5.89E-23	postive
CASP8	AC099343.2	0.418859	1.97E-21	postive
ZBP1	AC099343.2	0.550623	1.08E-38	postive
TNFSF10	AC099343.2	0.528055	3.54E-35	postive
TNFRSF1B	AC099343.2	0.480632	1.35E-28	postive
CYLD	AC099343.2	0.504126	9.97E-32	postive
CFLAR	AC099343.2	0.523298	1.81E-34	postive
ZBP1	AC023510.2	0.522037	2.77E-34	postive
TNFSF10	AC023510.2	0.406293	3.79E-20	postive
TNFRSF1B	AC023510.2	0.47924	2.03E-28	postive
CYLD	AC023510.2	0.601478	1.13E-47	postive
CFLAR	AC023510.2	0.547635	3.27E-38	postive
FLT3	AC023510.2	0.633374	3.48E-54	postive
BACH2	AC023510.2	0.478908	2.24E-28	postive
CD40	AC023510.2	0.58934	2.19E-45	postive
GATA3	AC068831.5	0.528591	2.94E-35	postive
EGFR	MIR2117HG	0.485679	3.00E-29	postive
TARDBP	ANKRD10-IT1	0.430026	1.27E-22	postive
BCL2	AC012313.5	0.411737	1.07E-20	postive
STAT3	SNHG25	-0.41616	3.75E-21	negative
SIRT3	AL118558.3	0.403659	6.95E-20	postive
DIABLO	AC097359.2	0.501855	2.05E-31	postive
PLK1	AC093010.2	-0.41142	1.15E-20	negative
TNF	AC244034.2	0.444194	3.40E-24	postive
TNFRSF1B	AC244034.2	0.418772	2.01E-21	postive
FLT3	AC244034.2	0.460493	4.25E-26	postive
CD40	AC244034.2	0.448538	1.08E-24	postive
TRIM11	AC016888.1	0.468872	4.06E-27	postive
RNF31	AC016888.1	0.414434	5.66E-21	postive
MAPK8	AL359922.2	0.413287	7.42E-21	postive
RNF31	AC125807.2	0.423079	7.07E-22	postive
SIRT3	AP004609.3	0.443445	4.14E-24	postive
GATA3	AC005476.2	0.406443	3.67E-20	postive
TARDBP	AC245060.5	0.448893	9.84E-25	postive
DDX58	NRIR	0.499747	3.99E-31	postive
SIRT3	ZBED5-AS1	0.536584	1.79E-36	postive
TNFSF10	AL121839.2	0.41394	6.36E-21	postive
CYLD	AL121839.2	0.458276	7.82E-26	postive
CFLAR	AL121839.2	0.408822	2.11E-20	postive
GATA3	AL121839.2	0.401209	1.21E-19	postive
TNF	LINC00324	0.417116	2.99E-21	postive
TNFRSF1B	LINC00324	0.406489	3.63E-20	postive
CYLD	LINC00324	0.447633	1.38E-24	postive

FLT3	LINC00324	0.455966	1.47E-25	postive
SIRT1	AL021368.2	0.429704	1.38E-22	postive
ATRX	AL021368.2	0.402948	8.17E-20	postive
FASLG	AC018755.4	0.403446	7.30E-20	postive
MLKL	AC018755.4	0.42935	1.51E-22	postive
RIPK3	AC018755.4	0.435784	2.98E-23	postive
TNF	AC018755.4	0.435389	3.30E-23	postive
ZBP1	AC018755.4	0.538873	7.90E-37	postive
TNFSF10	AC018755.4	0.439648	1.11E-23	postive
TNFRSF1B	AC018755.4	0.631978	6.96E-54	postive
CYLD	AC018755.4	0.530213	1.68E-35	postive
CFLAR	AC018755.4	0.545697	6.67E-38	postive
FLT3	AC018755.4	0.643371	2.19E-56	postive
CD40	AC018755.4	0.595413	1.61E-46	postive
STUB1	Z92544.1	0.41618	3.73E-21	postive
MAPK8	ZNF32-AS2	0.505472	6.48E-32	postive
SIRT1	ZNF32-AS2	0.449999	7.33E-25	postive
BACH2	AL118505.1	0.528222	3.34E-35	postive
GATA3	AL118505.1	0.904265	######	postive
MYCN	AL118505.1	0.717515	1.13E-75	postive
GATA3	AL031058.1	0.530646	1.44E-35	postive
MYCN	AL031058.1	0.40976	1.70E-20	postive
BACH2	AC068473.3	0.492855	3.40E-30	postive
GATA3	AC068473.3	0.739167	1.56E-82	postive
MYCN	AC068473.3	0.570718	4.69E-42	postive
MAPK8	LINC01772	0.40607	3.99E-20	postive
GATA3	MIR200CHG	0.464879	1.25E-26	postive
TRIM11	AL162377.1	0.451975	4.32E-25	postive
TNF	IFNG-AS1	0.445621	2.34E-24	postive
ZBP1	IFNG-AS1	0.61167	1.13E-49	postive
TNFSF10	IFNG-AS1	0.482135	8.65E-29	postive
TNFRSF1B	IFNG-AS1	0.523465	1.71E-34	postive
CYLD	IFNG-AS1	0.571392	3.58E-42	postive
CFLAR	IFNG-AS1	0.506282	5.00E-32	postive
FLT3	IFNG-AS1	0.613815	4.19E-50	postive
CD40	IFNG-AS1	0.562141	1.36E-40	postive
MLKL	AC078883.1	0.46781	5.49E-27	postive
CASP8	AC078883.1	0.40495	5.17E-20	postive
ZBP1	AC078883.1	0.607119	8.99E-49	postive
CYLD	AC078883.1	0.455389	1.72E-25	postive
CFLAR	AC078883.1	0.453412	2.93E-25	postive
FASLG	AC008760.2	0.427358	2.47E-22	postive
TNFRSF1B	AC008760.2	0.529062	2.50E-35	postive
TRIM11	SNHG11	0.436578	2.43E-23	postive

SIRT3	SNHG11	0.422478	8.19E-22	postive
CYLD	SNHG11	-0.42161	1.01E-21	negative
RIPK3	AC129492.1	0.421571	1.02E-21	postive
BACH2	AC004706.1	0.554094	2.95E-39	postive
GATA3	AC004706.1	0.55973	3.45E-40	postive
GATA3	SOX21-AS1	0.746398	5.61E-85	postive
MYCN	SOX21-AS1	0.500575	3.07E-31	postive
CYLD	EBLN3P	0.438224	1.60E-23	postive
SIRT1	EBLN3P	0.432947	6.12E-23	postive
ATRX	EBLN3P	0.438297	1.57E-23	postive
BRAF	AC000123.1	0.429601	1.42E-22	postive
TARDBP	AC000123.1	0.424362	5.17E-22	postive
GATA3	AC110619.1	0.635397	1.27E-54	postive
MYCN	AC110619.1	0.517215	1.40E-33	postive
DNMT1	AC099850.3	0.488812	1.17E-29	postive
HAT1	AC099850.3	0.477672	3.22E-28	postive
PLK1	AC099850.3	0.514906	3.01E-33	postive
TRAF2	AC147067.1	0.455198	1.81E-25	postive
TRIM11	AC002398.1	0.445838	2.21E-24	postive
TRAF2	AC002398.1	0.441672	6.56E-24	postive
ATRX	AC002398.1	-0.47265	1.38E-27	negative
TARDBP	AP4B1-AS1	0.455917	1.49E-25	postive
BCL2	AL033381.2	0.467884	5.38E-27	postive
RIPK3	AC008555.4	0.410029	1.59E-20	postive
TNF	AC008555.4	0.442133	5.82E-24	postive
ZBP1	AC008555.4	0.450321	6.73E-25	postive
TNFRSF1B	AC008555.4	0.471314	2.02E-27	postive
CFLAR	AC008555.4	0.416479	3.48E-21	postive
FLT3	AC008555.4	0.606112	1.42E-48	postive
CD40	AC008555.4	0.525875	7.50E-35	postive
TRIM11	AC136604.2	0.401965	1.02E-19	postive
DIABLO	AC136604.2	0.402456	9.14E-20	postive
FASLG	LINC01871	0.81183	######	postive
MLKL	LINC01871	0.543221	1.65E-37	postive
RIPK3	LINC01871	0.448427	1.11E-24	postive
TNF	LINC01871	0.424651	4.81E-22	postive
ZBP1	LINC01871	0.661128	1.67E-60	postive
TNFSF10	LINC01871	0.60529	2.05E-48	postive
TNFRSF1B	LINC01871	0.688361	2.13E-67	postive
CYLD	LINC01871	0.478353	2.64E-28	postive
CFLAR	LINC01871	0.525323	9.06E-35	postive
FLT3	LINC01871	0.402485	9.08E-20	postive
CD40	LINC01871	0.520588	4.52E-34	postive
TRIM11	AC006504.5	0.404328	5.96E-20	postive

TNFSF10	AC006504.5	-0.41669	3.30E-21	negative
DIABLO	AC006504.5	0.403302	7.54E-20	postive
BCL2	AC027097.2	0.427347	2.48E-22	postive
AXL	HS1BP3-IT1	0.484229	4.64E-29	postive
FASLG	LINC02362	0.471503	1.92E-27	postive
MLKL	LINC02362	0.431888	7.98E-23	postive
ZBP1	LINC02362	0.754857	6.04E-88	postive
TNFSF10	LINC02362	0.407148	3.12E-20	postive
TNFRSF1B	LINC02362	0.440209	9.59E-24	postive
CYLD	LINC02362	0.439038	1.30E-23	postive
CFLAR	LINC02362	0.458409	7.54E-26	postive
CD40	LINC02362	0.433275	5.63E-23	postive
ATRX	AL049869.2	0.429827	1.34E-22	postive
TARDBP	AC095057.3	0.492764	3.49E-30	postive
CYLD	LINC02422	0.461021	3.67E-26	postive
CFLAR	LINC02422	0.431834	8.09E-23	postive
FLT3	LINC02422	0.486637	2.25E-29	postive
BACH2	LINC02422	0.401756	1.07E-19	postive
CD40	LINC02422	0.526821	5.42E-35	postive
ATRX	ATP2A1-AS1	-0.41594	3.95E-21	negative
GATA3	CALML3-AS1	0.504167	9.84E-32	postive
MLKL	AC010247.1	0.497729	7.51E-31	postive
ZBP1	AC010247.1	0.417272	2.88E-21	postive
TNFRSF1B	AC010247.1	0.470569	2.51E-27	postive
CYLD	AC010247.1	0.422948	7.30E-22	postive
FLT3	AC010247.1	0.506612	4.50E-32	postive
CD40	AC010247.1	0.481981	9.05E-29	postive
RIPK3	LINC02361	0.455462	1.68E-25	postive
TNF	LINC02361	0.439782	1.07E-23	postive
TNFRSF1B	LINC02361	0.416453	3.50E-21	postive
FLT3	LINC02361	0.459577	5.47E-26	postive
ATRX	AC010834.3	0.507639	3.23E-32	postive
FASLG	LINC00861	0.404096	6.29E-20	postive
MLKL	LINC00861	0.424578	4.90E-22	postive
RIPK3	LINC00861	0.409948	1.62E-20	postive
TNF	LINC00861	0.496697	1.04E-30	postive
CASP8	LINC00861	0.444007	3.57E-24	postive
ZBP1	LINC00861	0.554835	2.23E-39	postive
TNFSF10	LINC00861	0.493509	2.78E-30	postive
TNFRSF1B	LINC00861	0.587652	4.49E-45	postive
CYLD	LINC00861	0.718223	6.89E-76	postive
CFLAR	LINC00861	0.61618	1.39E-50	postive
FLT3	LINC00861	0.762992	6.47E-91	postive
BACH2	LINC00861	0.412094	9.83E-21	postive

CD40	LINC00861	0.651371	3.31E-58	postive
CYLD	AC005332.7	0.461963	2.83E-26	postive
CFLAR	AC005332.7	0.404671	5.51E-20	postive
FLT3	AC005332.7	0.470128	2.84E-27	postive
BACH2	AC005332.7	0.40537	4.69E-20	postive
CD40	AC005332.7	0.423718	6.05E-22	postive
GATA3	AC080038.2	0.55445	2.58E-39	postive
MYCN	AC080038.2	0.480388	1.45E-28	postive
BCL2	SPART-AS1	0.406952	3.26E-20	postive
FASLG	AC002091.2	0.421929	9.36E-22	postive
RIPK3	AC002091.2	0.458134	8.13E-26	postive
ZBP1	AC002091.2	0.569261	8.37E-42	postive
TNFRSF1B	AC002091.2	0.650788	4.51E-58	postive
FLT3	AC002091.2	0.51264	6.35E-33	postive
FASLG	AC007728.2	0.664205	3.02E-61	postive
MLKL	AC007728.2	0.569958	6.35E-42	postive
RIPK3	AC007728.2	0.524768	1.10E-34	postive
TNF	AC007728.2	0.528081	3.51E-35	postive
CASP8	AC007728.2	0.453951	2.54E-25	postive
ZBP1	AC007728.2	0.716676	2.02E-75	postive
TNFSF10	AC007728.2	0.633458	3.34E-54	postive
TNFRSF1B	AC007728.2	0.792318	######	postive
CYLD	AC007728.2	0.744861	1.88E-84	postive
CFLAR	AC007728.2	0.698125	4.66E-70	postive
FLT3	AC007728.2	0.741315	2.99E-83	postive
CD40	AC007728.2	0.689217	1.26E-67	postive
ZBP1	LINC01353	0.415782	4.11E-21	postive
TNFSF10	AC009950.1	0.437932	1.72E-23	postive
CYLD	AC009950.1	0.535995	2.20E-36	postive
CFLAR	AC009950.1	0.430363	1.17E-22	postive
FLT3	AC009950.1	0.425682	3.74E-22	postive
DDX58	AC009950.1	0.668953	2.07E-62	postive
TRIM11	AL139288.1	0.434875	3.76E-23	postive
TARDBP	LINC01355	0.51995	5.60E-34	postive
FASLG	AL133371.2	0.42038	1.36E-21	postive
MLKL	AL133371.2	0.439737	1.08E-23	postive
RIPK3	AL133371.2	0.430047	1.27E-22	postive
TNF	AL133371.2	0.457993	8.45E-26	postive
ZBP1	AL133371.2	0.534883	3.26E-36	postive
TNFSF10	AL133371.2	0.491584	5.02E-30	postive
TNFRSF1B	AL133371.2	0.65545	3.71E-59	postive
CYLD	AL133371.2	0.615048	2.36E-50	postive
CFLAR	AL133371.2	0.571501	3.43E-42	postive
FLT3	AL133371.2	0.682735	6.53E-66	postive

CD40	AL133371.2	0.528889	2.65E-35	postive
MAPK8	AL353796.1	0.520136	5.26E-34	postive
MYC	SNHG29	0.452985	3.29E-25	postive
SIRT1	SNHG29	0.403793	6.74E-20	postive
FASLG	AC008105.2	0.470928	2.26E-27	postive
MLKL	AC008105.2	0.503785	1.11E-31	postive
RIPK3	AC008105.2	0.436113	2.74E-23	postive
TNF	AC008105.2	0.529733	1.98E-35	postive
CASP8	AC008105.2	0.4063	3.79E-20	postive
ZBP1	AC008105.2	0.596504	1.00E-46	postive
TNFSF10	AC008105.2	0.553785	3.31E-39	postive
TNFRSF1B	AC008105.2	0.570528	5.06E-42	postive
CYLD	AC008105.2	0.681789	1.15E-65	postive
CFLAR	AC008105.2	0.584926	1.41E-44	postive
FLT3	AC008105.2	0.678152	1.00E-64	postive
CD40	AC008105.2	0.616477	1.21E-50	postive
DIABLO	HEXA-AS1	0.425396	4.01E-22	postive
RNF31	AC007038.1	-0.47158	1.88E-27	negative
TARDBP	AC007038.1	0.494927	1.79E-30	postive
IDH2	DLX6-AS1	0.499811	3.91E-31	postive
TARDBP	PSPC1-AS2	0.534926	3.21E-36	postive
SIRT1	AC018752.1	0.475139	6.73E-28	postive
ATRX	AC018752.1	0.543985	1.25E-37	postive
TRIM11	AC131009.3	0.425052	4.36E-22	postive
STAT3	AC131009.3	-0.4025	9.05E-20	negative
FASLG	DBH-AS1	0.635349	1.30E-54	postive
RIPK3	DBH-AS1	0.415587	4.30E-21	postive
ZBP1	DBH-AS1	0.575237	7.63E-43	postive
TNFSF10	DBH-AS1	0.473839	9.80E-28	postive
TNFRSF1B	DBH-AS1	0.597854	5.56E-47	postive
CYLD	DBH-AS1	0.511124	1.04E-32	postive
CFLAR	DBH-AS1	0.530006	1.80E-35	postive
FLT3	DBH-AS1	0.453266	3.05E-25	postive
CD40	DBH-AS1	0.541708	2.85E-37	postive
DIABLO	NRAV	0.45603	1.44E-25	postive
TRIM11	AC008915.2	0.433256	5.66E-23	postive
CYLD	AC008915.2	-0.42083	1.22E-21	negative
STUB1	AC008915.2	0.430189	1.22E-22	postive
ATRX	AC008915.2	-0.41093	1.29E-20	negative
SIRT1	OIP5-AS1	0.47017	2.81E-27	postive
ATRX	OIP5-AS1	0.495154	1.67E-30	postive
FASLG	MIR155HG	0.633261	3.68E-54	postive
MLKL	MIR155HG	0.442008	6.01E-24	postive
ZBP1	MIR155HG	0.491851	4.62E-30	postive

TNFSF10	MIR155HG	0.512686	6.25E-33	postive
TNFRSF1B	MIR155HG	0.507623	3.25E-32	postive
CYLD	MIR155HG	0.521399	3.44E-34	postive
CFLAR	MIR155HG	0.455127	1.85E-25	postive
CD40	MIR155HG	0.435797	2.97E-23	postive
SIRT1	AC011912.1	0.405841	4.21E-20	postive
OTULIN	EGFR-AS1	0.482305	8.22E-29	postive
SIRT1	AC008669.1	0.475703	5.71E-28	postive
RIPK3	TNK2-AS1	0.426096	3.38E-22	postive
ZBP1	TNK2-AS1	0.517742	1.17E-33	postive
TNFRSF1B	TNK2-AS1	0.495776	1.38E-30	postive
CFLAR	TNK2-AS1	0.409538	1.79E-20	postive
FLT3	TNK2-AS1	0.520523	4.62E-34	postive
TRIM11	AC109322.1	0.410111	1.56E-20	postive
TRAF2	AC109322.1	0.400811	1.33E-19	postive
ATRX	AC109322.1	-0.40711	3.14E-20	negative
DIABLO	UBAC2-AS1	0.418254	2.27E-21	postive
TRIM11	NDUFB2-AS1	0.441534	6.80E-24	postive
ATRX	NDUFB2-AS1	-0.44146	6.94E-24	negative
FLT3	LINC00243	0.456666	1.21E-25	postive
ATRX	AC025917.1	0.458026	8.37E-26	postive
EGFR	KRT7-AS	0.452861	3.41E-25	postive
TARDBP	AL928654.2	0.41577	4.12E-21	postive
FASLG	AC011899.2	0.515132	2.79E-33	postive
RIPK3	AC011899.2	0.466243	8.54E-27	postive
ZBP1	AC011899.2	0.510857	1.14E-32	postive
TNFRSF1B	AC011899.2	0.614029	3.79E-50	postive
MYC	AC012615.4	0.429348	1.51E-22	postive
FAS	AC015911.3	0.414148	6.05E-21	postive
FASLG	AC015911.3	0.76194	1.59E-90	postive
MLKL	AC015911.3	0.496219	1.20E-30	postive
TNF	AC015911.3	0.42797	2.12E-22	postive
ZBP1	AC015911.3	0.627524	6.21E-53	postive
TNFSF10	AC015911.3	0.683964	3.11E-66	postive
TNFRSF1B	AC015911.3	0.654294	6.93E-59	postive
CYLD	AC015911.3	0.687922	2.79E-67	postive
CFLAR	AC015911.3	0.592703	5.21E-46	postive
FLT3	AC015911.3	0.529948	1.84E-35	postive
CD40	AC015911.3	0.472705	1.36E-27	postive
DDX58	AC015911.3	0.412511	8.91E-21	postive
FAS	TRG-AS1	0.478104	2.84E-28	postive
FASLG	TRG-AS1	0.628561	3.74E-53	postive
MLKL	TRG-AS1	0.432973	6.08E-23	postive
CASP8	TRG-AS1	0.411219	1.21E-20	postive

ZBP1	TRG-AS1	0.545206	7.99E-38	postive
TNFSF10	TRG-AS1	0.509335	1.87E-32	postive
TNFRSF1B	TRG-AS1	0.598665	3.90E-47	postive
CYLD	TRG-AS1	0.544602	9.96E-38	postive
CFLAR	TRG-AS1	0.553478	3.72E-39	postive
FLT3	TRG-AS1	0.501002	2.69E-31	postive
CD40	TRG-AS1	0.505544	6.33E-32	postive
FASLG	AC012236.1	0.411947	1.02E-20	postive
MLKL	AC012236.1	0.448272	1.16E-24	postive
ZBP1	AC012236.1	0.836799	######	postive
TNFRSF1B	AC012236.1	0.511288	9.88E-33	postive
CYLD	AC012236.1	0.450556	6.32E-25	postive
CFLAR	AC012236.1	0.505228	7.01E-32	postive
FLT3	AC012236.1	0.463405	1.89E-26	postive
CD40	AC012236.1	0.574616	9.81E-43	postive
ATRX	Z68871.1	0.582668	3.62E-44	postive
HSP90AA1	Z68871.1	0.417732	2.58E-21	postive
TARDBP	OCIAD1-AS1	0.435607	3.12E-23	postive
FLT3	AC104971.3	0.482512	7.73E-29	postive
CD40	AC104971.3	0.464074	1.57E-26	postive
TNFSF10	AC138207.1	0.42018	1.43E-21	postive
DIABLO	UBL7-AS1	0.40461	5.59E-20	postive
RIPK1	HCG18	0.446392	1.91E-24	postive
SLC39A7	HCG18	0.499472	4.35E-31	postive
RNF31	LINC00638	0.429791	1.35E-22	postive
FASLG	C9orf139	0.569071	9.03E-42	postive
MLKL	C9orf139	0.507609	3.26E-32	postive
RIPK3	C9orf139	0.486927	2.06E-29	postive
TNF	C9orf139	0.554979	2.11E-39	postive
ZBP1	C9orf139	0.634601	1.89E-54	postive
TNFSF10	C9orf139	0.549841	1.45E-38	postive
TNFRSF1B	C9orf139	0.675779	4.04E-64	postive
CYLD	C9orf139	0.583391	2.68E-44	postive
CFLAR	C9orf139	0.596256	1.12E-46	postive
FLT3	C9orf139	0.708554	5.07E-73	postive
CD40	C9orf139	0.599554	2.64E-47	postive
ATRX	CR936218.1	0.404811	5.34E-20	postive
TARDBP	CR936218.1	0.401997	1.01E-19	postive
MAP3K7	HMGN3-AS1	0.435424	3.27E-23	postive
FASLG	AL357033.4	0.432065	7.64E-23	postive
RIPK3	AL357033.4	0.583483	2.58E-44	postive
TNF	AL357033.4	0.449985	7.36E-25	postive
ZBP1	AL357033.4	0.564083	6.41E-41	postive
TNFRSF1B	AL357033.4	0.571376	3.60E-42	postive

CFLAR	AL357033.4	0.450523	6.38E-25	postive
FLT3	AL357033.4	0.50944	1.80E-32	postive
CD40	AL357033.4	0.462361	2.53E-26	postive
TRIM11	AC139530.1	0.450886	5.79E-25	postive
ATRX	AL683813.1	0.4641	1.56E-26	postive
TNF	AL109914.1	0.476101	5.09E-28	postive
ZBP1	AL109914.1	0.416565	3.41E-21	postive
TNFRSF1B	AL109914.1	0.440562	8.75E-24	postive
CYLD	AL109914.1	0.535972	2.22E-36	postive
CFLAR	AL109914.1	0.417873	2.49E-21	postive
FLT3	AL109914.1	0.637981	3.45E-55	postive
CD40	AL109914.1	0.507589	3.28E-32	postive
OTULIN	AC010491.1	0.402592	8.86E-20	postive
ATRX	AP002761.3	-0.40484	5.30E-20	negative
BRAF	AC087071.2	0.477962	2.96E-28	postive
USP22	AC015922.2	0.456064	1.43E-25	postive
FASLG	AC008750.1	0.449954	7.42E-25	postive
MLKL	AC008750.1	0.440273	9.43E-24	postive
RIPK3	AC008750.1	0.416187	3.73E-21	postive
TNF	AC008750.1	0.403197	7.72E-20	postive
ZBP1	AC008750.1	0.49105	5.91E-30	postive
TNFSF10	AC008750.1	0.472358	1.50E-27	postive
TNFRSF1B	AC008750.1	0.608732	4.32E-49	postive
CYLD	AC008750.1	0.545185	8.05E-38	postive
CFLAR	AC008750.1	0.519763	5.96E-34	postive
FLT3	AC008750.1	0.558439	5.67E-40	postive
CD40	AC008750.1	0.563184	9.10E-41	postive
FASLG	LINC02285	0.486941	2.05E-29	postive
MLKL	LINC02285	0.471224	2.08E-27	postive
RIPK3	LINC02285	0.528036	3.57E-35	postive
TNF	LINC02285	0.435916	2.88E-23	postive
ZBP1	LINC02285	0.559531	3.73E-40	postive
TNFSF10	LINC02285	0.458222	7.93E-26	postive
TNFRSF1B	LINC02285	0.730897	7.80E-80	postive
CYLD	LINC02285	0.52285	2.10E-34	postive
CFLAR	LINC02285	0.519581	6.34E-34	postive
FLT3	LINC02285	0.648298	1.68E-57	postive
CD40	LINC02285	0.56155	1.71E-40	postive
FASLG	AL683807.1	0.475525	6.01E-28	postive
MLKL	AL683807.1	0.481785	9.59E-29	postive
RIPK3	AL683807.1	0.414765	5.23E-21	postive
TNF	AL683807.1	0.531187	1.19E-35	postive
CASP8	AL683807.1	0.426913	2.76E-22	postive
ZBP1	AL683807.1	0.555111	2.01E-39	postive

TNFSF10	AL683807.1	0.530517	1.51E-35	postive
TNFRSF1B	AL683807.1	0.545084	8.35E-38	postive
CYLD	AL683807.1	0.644611	1.15E-56	postive
CFLAR	AL683807.1	0.580879	7.60E-44	postive
FLT3	AL683807.1	0.596743	9.04E-47	postive
CD40	AL683807.1	0.581626	5.58E-44	postive
TARDBP	AC073655.2	0.400229	1.51E-19	postive
FASLG	LINC00954	0.407569	2.83E-20	postive
ZBP1	LINC00954	0.408851	2.10E-20	postive
TNFSF10	LINC00954	0.419488	1.69E-21	postive
TNFRSF1B	LINC00954	0.508463	2.47E-32	postive
CYLD	LINC00954	0.52972	1.99E-35	postive
CFLAR	LINC00954	0.489413	9.73E-30	postive
FLT3	LINC00954	0.506277	5.01E-32	postive
CD40	LINC00954	0.51296	5.71E-33	postive
MAPK8	AL132656.2	0.499996	3.69E-31	postive
SIRT1	AL132656.2	0.46541	1.08E-26	postive
CYLD	LINC00926	0.469286	3.61E-27	postive
CFLAR	LINC00926	0.405697	4.35E-20	postive
FLT3	LINC00926	0.549111	1.90E-38	postive
BACH2	LINC00926	0.506523	4.63E-32	postive
CD40	LINC00926	0.544038	1.22E-37	postive
RIPK3	AC010247.2	0.471472	1.94E-27	postive
TNF	AC010247.2	0.534322	3.98E-36	postive
ZBP1	AC010247.2	0.612119	9.16E-50	postive
TNFRSF1B	AC010247.2	0.641083	7.11E-56	postive
CYLD	AC010247.2	0.538602	8.70E-37	postive
CFLAR	AC010247.2	0.502655	1.59E-31	postive
FLT3	AC010247.2	0.780326	1.15E-97	postive
BACH2	AC010247.2	0.446764	1.73E-24	postive
CD40	AC010247.2	0.630231	1.65E-53	postive
FASLG	LINC02446	0.761523	2.27E-90	postive
MLKL	LINC02446	0.511213	1.01E-32	postive
ZBP1	LINC02446	0.621839	9.62E-52	postive
TNFSF10	LINC02446	0.587003	5.90E-45	postive
TNFRSF1B	LINC02446	0.63139	9.31E-54	postive
CYLD	LINC02446	0.49396	2.42E-30	postive
CFLAR	LINC02446	0.529054	2.51E-35	postive
FLT3	LINC02446	0.405196	4.88E-20	postive
CD40	LINC02446	0.466703	7.50E-27	postive
RIPK3	AL357033.3	0.502349	1.75E-31	postive
ZBP1	AL357033.3	0.453163	3.14E-25	postive
TNFRSF1B	AL357033.3	0.526433	6.19E-35	postive
FLT3	AL357033.3	0.476069	5.14E-28	postive

ATRX	AC008770.3	0.418275	2.26E-21	postive
GATA3	AC079414.3	0.460689	4.02E-26	postive
MYCN	AC079414.3	0.46952	3.38E-27	postive
FASLG	AC012645.3	0.486571	2.30E-29	postive
MLKL	AC012645.3	0.480661	1.34E-28	postive
RIPK3	AC012645.3	0.504621	8.51E-32	postive
TNF	AC012645.3	0.584302	1.83E-44	postive
CASP8	AC012645.3	0.425248	4.16E-22	postive
ZBP1	AC012645.3	0.700101	1.31E-70	postive
TNFSF10	AC012645.3	0.503767	1.12E-31	postive
TNFRSF1B	AC012645.3	0.683158	5.06E-66	postive
CYLD	AC012645.3	0.664015	3.36E-61	postive
CFLAR	AC012645.3	0.641175	6.79E-56	postive
FLT3	AC012645.3	0.794073	######	postive
CD40	AC012645.3	0.691139	3.83E-68	postive
FASLG	AC020659.1	0.466939	7.02E-27	postive
MLKL	AC020659.1	0.438337	1.55E-23	postive
RIPK3	AC020659.1	0.405751	4.30E-20	postive
TNF	AC020659.1	0.48308	6.53E-29	postive
CASP8	AC020659.1	0.433762	4.98E-23	postive
ZBP1	AC020659.1	0.552256	5.88E-39	postive
TNFSF10	AC020659.1	0.562548	1.16E-40	postive
TNFRSF1B	AC020659.1	0.551108	9.03E-39	postive
CYLD	AC020659.1	0.678031	1.08E-64	postive
CFLAR	AC020659.1	0.616101	1.44E-50	postive
FLT3	AC020659.1	0.648541	1.48E-57	postive
CD40	AC020659.1	0.58645	7.45E-45	postive
BCL2	GNG12-AS1	0.414099	6.12E-21	postive
CD40	GNG12-AS1	-0.40734	2.98E-20	negative
BACH2	AL645608.6	0.498608	5.70E-31	postive
GATA3	AL645608.6	0.675347	5.20E-64	postive
MYCN	AL645608.6	0.646791	3.71E-57	postive
IDH2	ZNF710-AS1	0.563884	6.93E-41	postive
FASLG	AC002091.1	0.490976	6.04E-30	postive
RIPK3	AC002091.1	0.51938	6.78E-34	postive
TNF	AC002091.1	0.413927	6.38E-21	postive
ZBP1	AC002091.1	0.593511	3.68E-46	postive
TNFRSF1B	AC002091.1	0.652673	1.65E-58	postive
FLT3	AC002091.1	0.491766	4.75E-30	postive
CD40	AC002091.1	0.414231	5.93E-21	postive
TNFRSF1B	AC092809.2	0.475421	6.20E-28	postive
RIPK3	AC145098.1	0.478278	2.70E-28	postive
TNF	AC145098.1	0.460894	3.80E-26	postive
ZBP1	AC145098.1	0.509523	1.76E-32	postive

TNFRSF1B	AC145098.1	0.591142	1.02E-45	postive
CFLAR	AC145098.1	0.445268	2.57E-24	postive
FLT3	AC145098.1	0.527734	3.96E-35	postive
CD40	AC145098.1	0.503953	1.05E-31	postive
ZBP1	AC246787.2	0.619842	2.48E-51	postive
TNFRSF1B	AC246787.2	0.465286	1.12E-26	postive
CFLAR	AC246787.2	0.404726	5.44E-20	postive
FLT3	AC246787.2	0.517131	1.44E-33	postive
CD40	AC246787.2	0.466993	6.92E-27	postive
BCL2	TEX41	0.485208	3.46E-29	postive
RIPK3	LINC01857	0.460579	4.15E-26	postive
TNF	LINC01857	0.502314	1.77E-31	postive
ZBP1	LINC01857	0.594138	2.80E-46	postive
TNFRSF1B	LINC01857	0.593053	4.48E-46	postive
CYLD	LINC01857	0.539751	5.76E-37	postive
CFLAR	LINC01857	0.51737	1.33E-33	postive
FLT3	LINC01857	0.706108	2.58E-72	postive
BACH2	LINC01857	0.458611	7.13E-26	postive
CD40	LINC01857	0.667374	5.08E-62	postive
RNF31	AC120053.1	-0.43595	2.86E-23	negative
FAS	AC079015.1	0.426756	2.87E-22	postive
FASLG	AC079015.1	0.751298	1.11E-86	postive
MLKL	AC079015.1	0.428387	1.91E-22	postive
ZBP1	AC079015.1	0.53337	5.56E-36	postive
TNFSF10	AC079015.1	0.591626	8.27E-46	postive
TNFRSF1B	AC079015.1	0.627656	5.82E-53	postive
CYLD	AC079015.1	0.611382	1.29E-49	postive
CFLAR	AC079015.1	0.577355	3.23E-43	postive
FLT3	AC079015.1	0.457593	9.42E-26	postive
CD40	AC079015.1	0.414953	5.00E-21	postive
TNFRSF1B	IL10RB-DT	0.41317	7.63E-21	postive
SIRT3	FOXN3-AS1	0.423219	6.83E-22	postive
MAP3K7	FOXN3-AS1	-0.40228	9.51E-20	negative
GATA3	AC010503.4	0.580475	8.97E-44	postive
BACH2	LINC02247	0.441948	6.11E-24	postive
GATA3	LINC02247	0.879743	######	postive
MYCN	LINC02247	0.612764	6.81E-50	postive
GATA3	PGM5-AS1	0.419714	1.60E-21	postive
FASLG	AC108134.3	0.647277	2.88E-57	postive
MLKL	AC108134.3	0.552417	5.53E-39	postive
RIPK3	AC108134.3	0.467519	5.96E-27	postive
TNF	AC108134.3	0.466402	8.17E-27	postive
ZBP1	AC108134.3	0.630289	1.60E-53	postive
TNFSF10	AC108134.3	0.643363	2.20E-56	postive

TNFRSF1B	AC108134.3	0.672505	2.71E-63	postive
CYLD	AC108134.3	0.571821	3.02E-42	postive
CFLAR	AC108134.3	0.584774	1.50E-44	postive
FLT3	AC108134.3	0.520239	5.08E-34	postive
CD40	AC108134.3	0.541156	3.48E-37	postive
PLK1	AC012073.1	0.55207	6.30E-39	postive
FASLG	AC006369.1	0.461462	3.25E-26	postive
MLKL	AC006369.1	0.420185	1.43E-21	postive
RIPK3	AC006369.1	0.419903	1.53E-21	postive
TNF	AC006369.1	0.464821	1.27E-26	postive
ZBP1	AC006369.1	0.573613	1.47E-42	postive
TNFSF10	AC006369.1	0.445615	2.34E-24	postive
TNFRSF1B	AC006369.1	0.600021	2.15E-47	postive
CYLD	AC006369.1	0.555317	1.86E-39	postive
CFLAR	AC006369.1	0.500612	3.04E-31	postive
FLT3	AC006369.1	0.694363	5.08E-69	postive
CD40	AC006369.1	0.537697	1.20E-36	postive
FASLG	PSMB8-AS1	0.499231	4.69E-31	postive
MLKL	PSMB8-AS1	0.45596	1.47E-25	postive
TNF	PSMB8-AS1	0.471297	2.04E-27	postive
CASP8	PSMB8-AS1	0.414222	5.95E-21	postive
ZBP1	PSMB8-AS1	0.619413	3.04E-51	postive
TNFSF10	PSMB8-AS1	0.504754	8.16E-32	postive
TNFRSF1B	PSMB8-AS1	0.554024	3.03E-39	postive
CYLD	PSMB8-AS1	0.64307	2.56E-56	postive
CFLAR	PSMB8-AS1	0.583816	2.25E-44	postive
FLT3	PSMB8-AS1	0.563197	9.05E-41	postive
CD40	PSMB8-AS1	0.552263	5.86E-39	postive
TARDBP	AC026471.1	-0.40837	2.34E-20	negative
TRIM11	AL121944.1	0.409598	1.76E-20	postive
TRIM11	AC012313.1	0.437049	2.16E-23	postive
RNF31	AC012313.1	0.458775	6.82E-26	postive
BACH2	CRIM1-DT	0.447931	1.27E-24	postive
GATA3	CRIM1-DT	0.732816	1.88E-80	postive
MYCN	CRIM1-DT	0.614339	3.28E-50	postive
TNFRSF1B	AL035530.1	0.410638	1.38E-20	postive
TARDBP	AC105339.2	0.414912	5.05E-21	postive
FASLG	AC008083.3	0.659736	3.59E-60	postive
MLKL	AC008083.3	0.517852	1.13E-33	postive
RIPK3	AC008083.3	0.419856	1.55E-21	postive
TNF	AC008083.3	0.440642	8.57E-24	postive
ZBP1	AC008083.3	0.698754	3.12E-70	postive
TNFSF10	AC008083.3	0.604897	2.45E-48	postive
TNFRSF1B	AC008083.3	0.7253	4.59E-78	postive

CYLD	AC008083.3	0.66579	1.24E-61	postive
CFLAR	AC008083.3	0.661172	1.63E-60	postive
FLT3	AC008083.3	0.651381	3.29E-58	postive
CD40	AC008083.3	0.606262	1.32E-48	postive
FASLG	LINC00528	0.498111	6.66E-31	postive
MLKL	LINC00528	0.519557	6.39E-34	postive
RIPK3	LINC00528	0.494709	1.92E-30	postive
TNF	LINC00528	0.540277	4.77E-37	postive
CASP8	LINC00528	0.435985	2.83E-23	postive
ZBP1	LINC00528	0.719634	2.57E-76	postive
TNFSF10	LINC00528	0.516927	1.54E-33	postive
TNFRSF1B	LINC00528	0.662783	6.67E-61	postive
CYLD	LINC00528	0.645376	7.76E-57	postive
CFLAR	LINC00528	0.633099	4.00E-54	postive
FLT3	LINC00528	0.728421	4.79E-79	postive
CD40	LINC00528	0.697859	5.53E-70	postive
SIRT3	DNAJC27-AS1	0.406598	3.54E-20	postive
TRIM11	AC110285.5	0.407406	2.93E-20	postive
TARDBP	AC127024.4	0.419448	1.71E-21	postive
BACH2	AC008687.3	0.460254	4.54E-26	postive
GATA3	AC008687.3	0.810476	######	postive
MYCN	AC008687.3	0.645251	8.28E-57	postive
CYLD	AC090948.3	0.444919	2.81E-24	postive
FLT3	AC090948.3	0.472923	1.28E-27	postive
CD40	AC090948.3	0.457921	8.61E-26	postive
FASLG	AL157871.2	0.505927	5.60E-32	postive
DIABLO	ATXN2-AS	0.419725	1.60E-21	postive
MYC	PROSER2-AS1	0.405399	4.66E-20	postive
MLKL	AL731567.1	0.424509	4.98E-22	postive
TNF	AL731567.1	0.516777	1.62E-33	postive
CASP8	AL731567.1	0.417322	2.84E-21	postive
ZBP1	AL731567.1	0.488625	1.24E-29	postive
TNFSF10	AL731567.1	0.441124	7.56E-24	postive
TNFRSF1B	AL731567.1	0.543874	1.30E-37	postive
CYLD	AL731567.1	0.612095	9.27E-50	postive
CFLAR	AL731567.1	0.51335	5.03E-33	postive
FLT3	AL731567.1	0.626279	1.14E-52	postive
CD40	AL731567.1	0.599352	2.88E-47	postive
FASLG	RERE-AS1	0.448746	1.02E-24	postive
MLKL	RERE-AS1	0.453581	2.80E-25	postive
TNF	RERE-AS1	0.40533	4.74E-20	postive
CASP8	RERE-AS1	0.416036	3.86E-21	postive
ZBP1	RERE-AS1	0.540281	4.76E-37	postive
TNFSF10	RERE-AS1	0.479682	1.79E-28	postive

TNFRSF1B	RERE-AS1	0.511647	8.79E-33	postive
CYLD	RERE-AS1	0.606819	1.03E-48	postive
CFLAR	RERE-AS1	0.578827	1.77E-43	postive
FLT3	RERE-AS1	0.583517	2.54E-44	postive
CD40	RERE-AS1	0.577792	2.70E-43	postive
BACH2	LINC00640	0.504522	8.78E-32	postive
GATA3	LINC00640	0.908111	######	postive
MYCN	LINC00640	0.696467	1.34E-69	postive
TRAF2	NPTN-IT1	-0.4016	1.11E-19	negative
ATRX	NPTN-IT1	0.504236	9.63E-32	postive
ATRX	CHKB-DT	-0.40186	1.05E-19	negative
FASLG	AC007991.2	0.579529	1.32E-43	postive
ZBP1	AC007991.2	0.427445	2.42E-22	postive
TNFSF10	AC007991.2	0.493199	3.06E-30	postive
CYLD	AC007991.2	0.413697	6.73E-21	postive
CFLAR	AC007991.2	0.424632	4.84E-22	postive
CYLD	AL021707.8	0.46129	3.41E-26	postive
GATA3	AC087491.1	0.533997	4.46E-36	postive
MYCN	AC087491.1	0.427458	2.41E-22	postive
MAPK8	AL139407.1	0.404718	5.45E-20	postive
IPMK	AL139407.1	0.432201	7.38E-23	postive
RIPK3	LINC02132	0.451566	4.82E-25	postive
TNF	LINC02132	0.509699	1.66E-32	postive
ZBP1	LINC02132	0.551842	6.87E-39	postive
TNFRSF1B	LINC02132	0.560296	2.78E-40	postive
CYLD	LINC02132	0.51973	6.03E-34	postive
CFLAR	LINC02132	0.470081	2.88E-27	postive
FLT3	LINC02132	0.772219	1.96E-94	postive
BACH2	LINC02132	0.484628	4.11E-29	postive
CD40	LINC02132	0.615692	1.75E-50	postive
TRIM11	PXN-AS1	0.4056	4.45E-20	postive
DIABLO	PXN-AS1	0.492468	3.83E-30	postive
DIABLO	ATP6V1B1-AS1	0.427695	2.27E-22	postive
USP22	AC087294.1	0.420897	1.20E-21	postive
TARDBP	AP002907.1	0.407768	2.70E-20	postive
FAS	BHLHE40-AS1	0.406896	3.30E-20	postive
FASLG	BHLHE40-AS1	0.782432	1.59E-98	postive
MLKL	BHLHE40-AS1	0.447744	1.34E-24	postive
ZBP1	BHLHE40-AS1	0.523598	1.63E-34	postive
TNFSF10	BHLHE40-AS1	0.627633	5.88E-53	postive
TNFRSF1B	BHLHE40-AS1	0.675794	4.00E-64	postive
CYLD	BHLHE40-AS1	0.57757	2.95E-43	postive
CFLAR	BHLHE40-AS1	0.540063	5.15E-37	postive

CD40	BHLHE40-AS1	0.431735	8.30E-23	postive
TARDBP	AC010976.1	0.503134	1.37E-31	postive
ATRX	AC004112.1	0.464365	1.45E-26	postive
FLT3	AC103858.1	0.521533	3.29E-34	postive
TARDBP	AC021078.1	0.431545	8.70E-23	postive
IPMK	GK-AS1	0.401524	1.13E-19	postive
FASLG	LINC00539	0.604826	2.53E-48	postive
ZBP1	LINC00539	0.447031	1.61E-24	postive
TNFSF10	LINC00539	0.418446	2.17E-21	postive
TNFRSF1B	LINC00539	0.444251	3.35E-24	postive
CYLD	LINC00539	0.453744	2.68E-25	postive
CFLAR	LINC00539	0.481869	9.36E-29	postive
ID1	AL121904.1	0.417931	2.46E-21	postive
FASLG	HCP5	0.515818	2.23E-33	postive
ZBP1	HCP5	0.490396	7.21E-30	postive
TNFSF10	HCP5	0.527779	3.90E-35	postive
TNFRSF1B	HCP5	0.482547	7.65E-29	postive
CYLD	HCP5	0.542364	2.25E-37	postive
CFLAR	HCP5	0.491678	4.87E-30	postive
FLT3	HCP5	0.413502	7.05E-21	postive
BACH2	AC034206.1	0.51351	4.77E-33	postive
GATA3	AC034206.1	0.879392	######	postive
MYCN	AC034206.1	0.689819	8.68E-68	postive
TARDBP	AC109460.2	0.464084	1.57E-26	postive
MLKL	FAM30A	0.434542	4.09E-23	postive
RIPK3	FAM30A	0.413009	7.92E-21	postive
TNF	FAM30A	0.506977	4.00E-32	postive
ZBP1	FAM30A	0.754793	6.37E-88	postive
TNFSF10	FAM30A	0.401154	1.23E-19	postive
TNFRSF1B	FAM30A	0.559301	4.07E-40	postive
CYLD	FAM30A	0.577525	3.01E-43	postive
CFLAR	FAM30A	0.561497	1.75E-40	postive
FLT3	FAM30A	0.716547	2.21E-75	postive
BACH2	FAM30A	0.43715	2.10E-23	postive
CD40	FAM30A	0.638973	2.09E-55	postive
GATA3	AL354836.1	0.507837	3.03E-32	postive
MYCN	AL354836.1	0.491979	4.45E-30	postive
MLKL	AC099524.1	0.439453	1.16E-23	postive
RIPK3	AC099524.1	0.432831	6.30E-23	postive
TNF	AC099524.1	0.455752	1.56E-25	postive
ZBP1	AC099524.1	0.719117	3.69E-76	postive
TNFRSF1B	AC099524.1	0.588259	3.47E-45	postive
CYLD	AC099524.1	0.570521	5.07E-42	postive
CFLAR	AC099524.1	0.550048	1.34E-38	postive

FLT3	AC099524.1	0.711734	5.96E-74	postive
BACH2	AC099524.1	0.432768	6.40E-23	postive
CD40	AC099524.1	0.680809	2.07E-65	postive
CYLD	AC005332.2	0.531216	1.18E-35	postive
CFLAR	AC005332.2	0.44771	1.35E-24	postive
FLT3	AC005332.2	0.555953	1.46E-39	postive
BACH2	AC005332.2	0.501557	2.25E-31	postive
CD40	AC005332.2	0.433336	5.54E-23	postive
RIPK3	MIR3945HG	0.422164	8.84E-22	postive
FASLG	AC090152.1	0.469699	3.21E-27	postive
TNFRSF1B	AC090152.1	0.426612	2.97E-22	postive
GATA3	EMX2OS	0.411436	1.15E-20	postive
EGFR	EMX2OS	0.413707	6.72E-21	postive
MAPK8	AP005899.1	0.447251	1.52E-24	postive
TRIM11	AC067852.2	0.572307	2.48E-42	postive
SIRT3	AC067852.2	0.41775	2.57E-21	postive
DIABLO	AC067852.2	0.442792	4.90E-24	postive
STUB1	AC067852.2	0.447207	1.54E-24	postive
RNF31	AC067852.2	0.538493	9.05E-37	postive
TARDBP	AC067852.2	-0.4699	3.03E-27	negative
ATRX	AC006213.5	0.410948	1.29E-20	postive
TRIM11	IPO9-AS1	0.579058	1.61E-43	postive
ATRX	JARID2-AS1	-0.41318	7.61E-21	negative
FASLG	AC067945.2	0.513309	5.10E-33	postive
MLKL	AC067945.2	0.415217	4.70E-21	postive
ZBP1	AC067945.2	0.508805	2.22E-32	postive
TNFSF10	AC067945.2	0.561506	1.74E-40	postive
TNFRSF1B	AC067945.2	0.550857	9.91E-39	postive
CYLD	AC067945.2	0.653837	8.86E-59	postive
CFLAR	AC067945.2	0.557844	7.11E-40	postive
FLT3	AC067945.2	0.596743	9.04E-47	postive
CD40	AC067945.2	0.500308	3.34E-31	postive
FASLG	AC005083.1	0.567829	1.47E-41	postive
ZBP1	AC005083.1	0.479737	1.76E-28	postive
TNFSF10	AC005083.1	0.510797	1.16E-32	postive
TNFRSF1B	AC005083.1	0.563545	7.91E-41	postive
CYLD	AC005083.1	0.524475	1.21E-34	postive
CFLAR	AC005083.1	0.472749	1.34E-27	postive
FLT3	AC005083.1	0.433063	5.94E-23	postive
CYLD	AC025171.4	0.40973	1.71E-20	postive
GATA3	UNC5B-AS1	0.660952	1.84E-60	postive
MYCN	UNC5B-AS1	0.421994	9.21E-22	postive
CD40	AC119396.1	0.429059	1.62E-22	postive
ZBP1	AC008957.1	0.497053	9.27E-31	postive

TNFSF10	AC008957.1	0.441551	6.77E-24	postive
TNFRSF1B	AC008957.1	0.526893	5.29E-35	postive
CYLD	AC008957.1	0.492053	4.35E-30	postive
CFLAR	AC008957.1	0.445656	2.32E-24	postive
FLT3	AC008957.1	0.478512	2.52E-28	postive
CD40	AC008957.1	0.421911	9.40E-22	postive
BCL2	AL035587.1	0.406433	3.67E-20	postive
TARDBP	PSMA3-AS1	0.419275	1.78E-21	postive
FASLG	LINC00582	0.400047	1.58E-19	postive
ZBP1	LINC00582	0.715435	4.75E-75	postive
CD40	LINC00582	0.422909	7.37E-22	postive
TNFSF10	AC007611.1	-0.41766	2.62E-21	negative
TRIM11	PCAT6	0.445266	2.57E-24	postive
ATRX	PCAT6	-0.40904	2.01E-20	negative
FASLG	AL359076.1	0.450392	6.60E-25	postive
MLKL	AL359076.1	0.441345	7.14E-24	postive
ZBP1	AL359076.1	0.487323	1.83E-29	postive
TNFSF10	AL359076.1	0.557037	9.67E-40	postive
TNFRSF1B	AL359076.1	0.492918	3.33E-30	postive
CYLD	AL359076.1	0.614209	3.49E-50	postive
CFLAR	AL359076.1	0.530489	1.52E-35	postive
FLT3	AL359076.1	0.504922	7.73E-32	postive
CD40	AL359076.1	0.454242	2.35E-25	postive
FASLG	LINC02084	0.654918	4.95E-59	postive
MLKL	LINC02084	0.413915	6.40E-21	postive
RIPK3	LINC02084	0.460774	3.93E-26	postive
ZBP1	LINC02084	0.546637	4.73E-38	postive
TNFSF10	LINC02084	0.407249	3.04E-20	postive
TNFRSF1B	LINC02084	0.511756	8.48E-33	postive
CD40	LINC02084	0.441612	6.67E-24	postive
FASLG	AP003774.2	0.624128	3.21E-52	postive
MLKL	AP003774.2	0.527892	3.75E-35	postive
RIPK3	AP003774.2	0.514929	2.99E-33	postive
TNF	AP003774.2	0.481429	1.07E-28	postive
ZBP1	AP003774.2	0.773724	5.05E-95	postive
TNFSF10	AP003774.2	0.497962	6.98E-31	postive
TNFRSF1B	AP003774.2	0.738818	2.04E-82	postive
CYLD	AP003774.2	0.574178	1.17E-42	postive
CFLAR	AP003774.2	0.615188	2.21E-50	postive
FLT3	AP003774.2	0.690484	5.75E-68	postive
CD40	AP003774.2	0.670406	9.05E-63	postive
FASLG	AC009133.3	0.591381	9.18E-46	postive
MLKL	AC009133.3	0.491827	4.66E-30	postive
RIPK3	AC009133.3	0.484847	3.85E-29	postive

TNF	AC009133.3	0.549943	1.39E-38	postive
CASP8	AC009133.3	0.443648	3.92E-24	postive
ZBP1	AC009133.3	0.768317	6.31E-93	postive
TNFSF10	AC009133.3	0.569079	9.00E-42	postive
TNFRSF1B	AC009133.3	0.741672	2.27E-83	postive
CYLD	AC009133.3	0.723986	1.18E-77	postive
CFLAR	AC009133.3	0.671241	5.61E-63	postive
FLT3	AC009133.3	0.804342	######	postive
CD40	AC009133.3	0.678367	8.82E-65	postive
FAS	MIAT	0.403111	7.87E-20	postive
FASLG	MIAT	0.714653	8.14E-75	postive
MLKL	MIAT	0.492032	4.37E-30	postive
CASP8	MIAT	0.405692	4.36E-20	postive
ZBP1	MIAT	0.635183	1.41E-54	postive
TNFSF10	MIAT	0.621949	9.12E-52	postive
TNFRSF1B	MIAT	0.631998	6.90E-54	postive
CYLD	MIAT	0.639668	1.47E-55	postive
CFLAR	MIAT	0.632508	5.36E-54	postive
FLT3	MIAT	0.475703	5.71E-28	postive
CD40	MIAT	0.531308	1.14E-35	postive
TLR3	PAXIP1-AS2	0.439957	1.02E-23	postive
ATRX	PAXIP1-AS2	0.428071	2.07E-22	postive
TSC1	PPP1R26-AS1	0.517165	1.42E-33	postive
SIRT1	AC090198.1	0.410703	1.36E-20	postive
ATRX	AC090198.1	0.420469	1.33E-21	postive
FASLG	AC023825.2	0.45305	3.24E-25	postive
MLKL	AC023825.2	0.514722	3.20E-33	postive
ZBP1	AC023825.2	0.422331	8.49E-22	postive
TNFSF10	AC023825.2	0.468698	4.27E-27	postive
TNFRSF1B	AC023825.2	0.407541	2.84E-20	postive
CYLD	AC023825.2	0.480291	1.49E-28	postive
GATA3	AC245041.2	0.633661	3.02E-54	postive
EGFR	AC245041.2	0.412685	8.55E-21	postive
ATRX	AP000692.2	-0.43787	1.75E-23	negative
FASLG	AL355916.2	0.54816	2.70E-38	postive
ZBP1	AL355916.2	0.409441	1.83E-20	postive
TNFSF10	AL355916.2	0.491854	4.62E-30	postive
TNFRSF1B	AL355916.2	0.506074	5.34E-32	postive
CYLD	AL355916.2	0.540188	4.93E-37	postive
CFLAR	AL355916.2	0.51406	3.98E-33	postive
FLT3	AL355916.2	0.419937	1.52E-21	postive
ID1	AL035425.1	0.430343	1.18E-22	postive
CYLD	VIM-AS1	0.434216	4.44E-23	postive
FASLG	AL158071.3	0.696531	1.29E-69	postive

MLKL	AL158071.3	0.405707	4.34E-20	postive
ZBP1	AL158071.3	0.504863	7.88E-32	postive
TNFRSF1B	AL158071.3	0.566221	2.78E-41	postive
ATRX	AF241728.1	0.431702	8.37E-23	postive
BACH2	FAM41C	0.553097	4.29E-39	postive
GATA3	FAM41C	0.873694	######	postive
MYCN	FAM41C	0.750003	3.15E-86	postive
MAPK8	AL731577.2	0.400334	1.48E-19	postive
SIRT1	AL731577.2	0.49387	2.49E-30	postive
FASLG	AC003070.1	0.498928	5.16E-31	postive
MLKL	AC003070.1	0.456657	1.22E-25	postive
RIPK3	AC003070.1	0.424663	4.80E-22	postive
TNF	AC003070.1	0.471674	1.83E-27	postive
CASP8	AC003070.1	0.411509	1.13E-20	postive
ZBP1	AC003070.1	0.580117	1.04E-43	postive
TNFSF10	AC003070.1	0.564295	5.90E-41	postive
TNFRSF1B	AC003070.1	0.604667	2.71E-48	postive
CYLD	AC003070.1	0.676943	2.04E-64	postive
CFLAR	AC003070.1	0.627266	7.04E-53	postive
FLT3	AC003070.1	0.673867	1.23E-63	postive
CD40	AC003070.1	0.576992	3.74E-43	postive
TRIM11	YTHDF3-AS1	0.412971	7.99E-21	postive
MAP3K7	YTHDF3-AS1	-0.41315	7.66E-21	negative
ATRX	YTHDF3-AS1	-0.40725	3.04E-20	negative
MAP3K7	AL359715.2	0.492264	4.07E-30	postive
TNFSF10	AL359220.1	0.448288	1.16E-24	postive
TNFRSF1B	AL359220.1	0.417279	2.87E-21	postive
CYLD	AL359220.1	0.402726	8.60E-20	postive
CFLAR	AL359220.1	0.407179	3.09E-20	postive
FLT3	AL359220.1	0.440464	8.97E-24	postive
BACH2	AL359220.1	0.423941	5.73E-22	postive
GATA3	AL359220.1	0.557116	9.39E-40	postive
FASLG	LINC01281	0.5369	1.60E-36	postive
MLKL	LINC01281	0.422	9.20E-22	postive
TNF	LINC01281	0.441228	7.36E-24	postive
ZBP1	LINC01281	0.535381	2.74E-36	postive
TNFSF10	LINC01281	0.457469	9.75E-26	postive
TNFRSF1B	LINC01281	0.648284	1.69E-57	postive
CYLD	LINC01281	0.561383	1.83E-40	postive
CFLAR	LINC01281	0.505979	5.51E-32	postive
FLT3	LINC01281	0.629005	3.01E-53	postive
CD40	LINC01281	0.523825	1.51E-34	postive
MAPK8	SNHG1	0.402076	9.97E-20	postive
TARDBP	AC007066.2	0.449763	7.81E-25	postive

TNFSF10	CADM3-AS1	0.403651	6.96E-20	postive
CYLD	CADM3-AS1	0.496684	1.04E-30	postive
FLT3	CADM3-AS1	0.56923	8.47E-42	postive
ZBP1	AL121845.4	0.410091	1.57E-20	postive
MAP3K7	AL359715.1	0.506738	4.32E-32	postive
GATA3	AL023284.4	0.426195	3.29E-22	postive
TARDBP	MIR17HG	0.485705	2.98E-29	postive
MLKL	AC100861.1	0.497026	9.35E-31	postive
CYLD	AC100861.1	0.404238	6.09E-20	postive
FLT3	AC100861.1	0.419205	1.81E-21	postive
CD40	AC100861.1	0.493055	3.20E-30	postive
TARDBP	AC011825.2	0.421346	1.08E-21	postive
FASLG	LINC02528	0.817196	######	postive
MLKL	LINC02528	0.490368	7.27E-30	postive
ZBP1	LINC02528	0.505656	6.11E-32	postive
TNFSF10	LINC02528	0.630706	1.31E-53	postive
TNFRSF1B	LINC02528	0.637376	4.68E-55	postive
CYLD	LINC02528	0.531696	1.00E-35	postive
CFLAR	LINC02528	0.51104	1.07E-32	postive
TRIM11	FGD5-AS1	-0.42424	5.33E-22	negative
TRAF2	FGD5-AS1	-0.44937	8.67E-25	negative
MAP3K7	FGD5-AS1	0.433974	4.72E-23	postive
ATRX	FGD5-AS1	0.50139	2.38E-31	postive
TRIM11	AC114341.1	0.412916	8.10E-21	postive
ZBP1	AC022167.3	0.532277	8.16E-36	postive
FLT3	AC022167.3	0.43793	1.72E-23	postive
CD40	AC022167.3	0.410736	1.35E-20	postive
OTULIN	AL078612.2	0.448065	1.23E-24	postive
TRIM11	ZNF687-AS1	0.521282	3.58E-34	postive
DIABLO	ZNF687-AS1	0.410161	1.55E-20	postive
ATRX	AP000866.1	0.408166	2.46E-20	postive
TARDBP	AC127024.5	0.404319	5.97E-20	postive
RIPK3	AC093278.2	0.444586	3.07E-24	postive
TNFRSF1B	AC093278.2	0.452842	3.42E-25	postive
FLT3	AC093278.2	0.565096	4.32E-41	postive
BNIP3	AL162274.1	0.524556	1.18E-34	postive
TARDBP	AC096586.2	0.408675	2.19E-20	postive
TRIM11	AC004221.1	0.422773	7.62E-22	postive
CYLD	AC004221.1	-0.42586	3.57E-22	negative
BCL2	SNHG16	0.412117	9.77E-21	postive
TRIM11	AC040169.1	0.446052	2.09E-24	postive
ATRX	AC040169.1	-0.46322	1.99E-26	negative
ATRX	AC004908.1	0.422656	7.84E-22	postive
TARDBP	AC004908.1	0.439268	1.22E-23	postive

CYLD	AC016957.2	0.432114	7.54E-23	postive
CFLAR	AC016957.2	0.439976	1.02E-23	postive
FLT3	AC016957.2	0.42243	8.28E-22	postive
CD40	AC016957.2	0.464549	1.37E-26	postive
ID1	LINC02593	0.415469	4.42E-21	postive
BACH2	LINC02593	0.477102	3.80E-28	postive
GATA3	LINC02593	0.651941	2.44E-58	postive
MYCN	LINC02593	0.591127	1.02E-45	postive
EGFR	LINC02593	0.522917	2.06E-34	postive
FASLG	AC131097.3	0.462091	2.73E-26	postive
TNFRSF1B	AC131097.3	0.555344	1.84E-39	postive
GATA3	LINC02568	0.519816	5.86E-34	postive
MYCN	LINC02568	0.404814	5.33E-20	postive
FASLG	AC243960.1	0.417232	2.90E-21	postive
MLKL	AC243960.1	0.446716	1.75E-24	postive
TNF	AC243960.1	0.491771	4.74E-30	postive
CASP8	AC243960.1	0.446987	1.63E-24	postive
ZBP1	AC243960.1	0.582487	3.90E-44	postive
TNFSF10	AC243960.1	0.4983	6.28E-31	postive
TNFRSF1B	AC243960.1	0.565108	4.30E-41	postive
CYLD	AC243960.1	0.672935	2.11E-63	postive
CFLAR	AC243960.1	0.591089	1.04E-45	postive
FLT3	AC243960.1	0.682835	6.15E-66	postive
CD40	AC243960.1	0.650736	4.64E-58	postive
TNFRSF1B	AL161785.1	0.443703	3.87E-24	postive
MLKL	SENCR	0.431782	8.20E-23	postive
RIPK3	SENCR	0.471163	2.11E-27	postive
ZBP1	SENCR	0.452329	3.93E-25	postive
TNFRSF1B	SENCR	0.533636	5.06E-36	postive
FADD	KCTD21-AS1	0.466328	8.34E-27	postive
FASLG	AC007569.1	0.447288	1.51E-24	postive
MLKL	AC007569.1	0.46398	1.61E-26	postive
ZBP1	AC007569.1	0.829321	######	postive
TNFRSF1B	AC007569.1	0.56056	2.51E-40	postive
CYLD	AC007569.1	0.504411	9.10E-32	postive
CFLAR	AC007569.1	0.553257	4.04E-39	postive
FLT3	AC007569.1	0.504709	8.28E-32	postive
CD40	AC007569.1	0.504254	9.57E-32	postive
MYC	RBFADN	0.416032	3.87E-21	postive
ATRX	Z83843.1	0.445416	2.47E-24	postive
TARDBP	Z83843.1	0.424542	4.95E-22	postive
TRIM11	SUCLA2-AS1	0.464553	1.37E-26	postive
FASLG	AC243829.4	0.760428	5.75E-90	postive
MLKL	AC243829.4	0.400164	1.54E-19	postive

RIPK3	AC243829.4	0.473863	9.73E-28	postive
ZBP1	AC243829.4	0.516247	1.93E-33	postive
TNFSF10	AC243829.4	0.452361	3.90E-25	postive
TNFRSF1B	AC243829.4	0.610212	2.20E-49	postive
CFLAR	AC243829.4	0.427846	2.19E-22	postive
FASLG	TSPOAP1-AS1	0.416629	3.36E-21	postive
TNF	TSPOAP1-AS1	0.43056	1.11E-22	postive
ZBP1	TSPOAP1-AS1	0.472682	1.37E-27	postive
TNFRSF1B	TSPOAP1-AS1	0.553706	3.41E-39	postive
CYLD	TSPOAP1-AS1	0.494835	1.85E-30	postive
CFLAR	TSPOAP1-AS1	0.460214	4.59E-26	postive
FLT3	TSPOAP1-AS1	0.604449	2.99E-48	postive
CD40	TSPOAP1-AS1	0.446218	2.00E-24	postive
MLKL	C1RL-AS1	0.442687	5.04E-24	postive
CASP8	C1RL-AS1	0.461087	3.60E-26	postive
ZBP1	C1RL-AS1	0.4549	1.96E-25	postive
TNFSF10	C1RL-AS1	0.482395	8.01E-29	postive
TNFRSF1B	C1RL-AS1	0.48533	3.34E-29	postive
CYLD	C1RL-AS1	0.561497	1.75E-40	postive
CFLAR	C1RL-AS1	0.490347	7.32E-30	postive
FLT3	C1RL-AS1	0.549071	1.93E-38	postive
CD40	C1RL-AS1	0.517206	1.40E-33	postive
GATA3	HOXA10-AS	0.663082	5.65E-61	postive
MYCN	HOXA10-AS	0.484934	3.76E-29	postive
FASLG	AL023653.1	0.501107	2.60E-31	postive
MLKL	AL023653.1	0.463718	1.73E-26	postive
RIPK3	AL023653.1	0.464969	1.22E-26	postive
TNF	AL023653.1	0.519633	6.23E-34	postive
CASP8	AL023653.1	0.404493	5.74E-20	postive
ZBP1	AL023653.1	0.63847	2.69E-55	postive
TNFSF10	AL023653.1	0.527764	3.92E-35	postive
TNFRSF1B	AL023653.1	0.677161	1.80E-64	postive
CYLD	AL023653.1	0.666204	9.84E-62	postive
CFLAR	AL023653.1	0.623762	3.83E-52	postive
FLT3	AL023653.1	0.734436	5.62E-81	postive
CD40	AL023653.1	0.689643	9.68E-68	postive
AXL	NKILA	0.506907	4.09E-32	postive
CYLD	AL662844.4	0.48396	5.02E-29	postive
CFLAR	AL662844.4	0.478223	2.74E-28	postive
FLT3	AL662844.4	0.433026	6.00E-23	postive
CD40	AL662844.4	0.428448	1.89E-22	postive
TARDBP	LINC01547	-0.40192	1.03E-19	negative
SIRT3	AC133552.5	0.458516	7.32E-26	postive
ATRX	AC133552.5	-0.40277	8.51E-20	negative

FADD	AP002336.2	0.445362	2.50E-24	postive
TNFSF10	LINC01094	0.465268	1.12E-26	postive
TNFRSF1B	LINC01094	0.486334	2.47E-29	postive
CYLD	LINC01094	0.437187	2.08E-23	postive
AXL	LINC01094	0.44392	3.65E-24	postive
DIABLO	LINC01679	0.434789	3.84E-23	postive
TNFRSF1B	HLA-DQB1-AS1	0.411576	1.11E-20	postive
CYLD	HLA-DQB1-AS1	0.429732	1.37E-22	postive
AXL	LINC00460	0.437704	1.83E-23	postive
ZBP1	AC242842.1	0.406354	3.74E-20	postive
CD40	AC242842.1	0.461244	3.45E-26	postive
ATRX	AC009120.3	0.42988	1.32E-22	postive
TNF	AC097375.1	0.41833	2.23E-21	postive
TNFRSF1B	AC097375.1	0.470876	2.30E-27	postive
CYLD	AC097375.1	0.500533	3.11E-31	postive
CFLAR	AC097375.1	0.40513	4.96E-20	postive
FLT3	AC097375.1	0.778377	7.11E-97	postive
CD40	AC097375.1	0.488366	1.34E-29	postive
ATRX	AC234775.2	0.520868	4.11E-34	postive
RIPK3	LINC02158	0.497036	9.32E-31	postive
TNFRSF1A	AC009509.4	0.424816	4.62E-22	postive
MLKL	ITGB2-AS1	0.425944	3.50E-22	postive
RIPK3	ITGB2-AS1	0.432715	6.48E-23	postive
TNF	ITGB2-AS1	0.535827	2.34E-36	postive
CASP8	ITGB2-AS1	0.42532	4.08E-22	postive
ZBP1	ITGB2-AS1	0.541043	3.62E-37	postive
TNFSF10	ITGB2-AS1	0.459314	5.88E-26	postive
TNFRSF1B	ITGB2-AS1	0.531445	1.09E-35	postive
CYLD	ITGB2-AS1	0.616617	1.13E-50	postive
CFLAR	ITGB2-AS1	0.582885	3.31E-44	postive
FLT3	ITGB2-AS1	0.668699	2.40E-62	postive
CD40	ITGB2-AS1	0.637187	5.15E-55	postive
GATA3	LINC01133	0.664613	2.40E-61	postive
FASLG	AC243829.1	0.488362	1.34E-29	postive
TNFRSF1B	AC243829.1	0.421351	1.08E-21	postive
MAPK8	AL731566.2	0.410389	1.47E-20	postive
IPMK	AL731566.2	0.511923	8.03E-33	postive
SIRT1	AL731566.2	0.408494	2.28E-20	postive
TNFRSF1B	LINC01197	0.428274	1.97E-22	postive
CYLD	LINC01197	0.425132	4.28E-22	postive
FLT3	LINC01197	0.609944	2.49E-49	postive
CYLD	PPP3CB-AS1	0.491278	5.51E-30	postive

CFLAR	PPP3CB-AS1	0.453992	2.51E-25	postive
SIRT1	PPP3CB-AS1	0.426812	2.83E-22	postive
FASLG	AC005840.4	0.467564	5.88E-27	postive
TNF	AC005840.4	0.427743	2.25E-22	postive
ZBP1	AC005840.4	0.462735	2.28E-26	postive
TNFSF10	AC005840.4	0.404256	6.06E-20	postive
CYLD	AC005840.4	0.40236	9.34E-20	postive
CFLAR	AC005840.4	0.405869	4.18E-20	postive
SIRT1	WDFY3-AS2	0.404362	5.91E-20	postive
BACH2	SNTG2-AS1	0.517069	1.47E-33	postive
GATA3	SNTG2-AS1	0.73277	1.95E-80	postive
MYCN	SNTG2-AS1	0.523017	1.99E-34	postive
FASLG	LINC00996	0.434389	4.25E-23	postive
MLKL	LINC00996	0.48461	4.14E-29	postive
RIPK3	LINC00996	0.426575	3.00E-22	postive
TNF	LINC00996	0.500312	3.34E-31	postive
CASP8	LINC00996	0.434941	3.69E-23	postive
ZBP1	LINC00996	0.62817	4.53E-53	postive
TNFSF10	LINC00996	0.531581	1.04E-35	postive
TNFRSF1B	LINC00996	0.665731	1.28E-61	postive
CYLD	LINC00996	0.699697	1.70E-70	postive
CFLAR	LINC00996	0.601672	1.03E-47	postive
FLT3	LINC00996	0.811451	######	postive
BACH2	LINC00996	0.401624	1.10E-19	postive
CD40	LINC00996	0.664571	2.46E-61	postive
EGFR	AL356417.2	0.75478	6.44E-88	postive
FASLG	AC090559.1	0.549967	1.38E-38	postive
MLKL	AC090559.1	0.401625	1.10E-19	postive
RIPK3	AC090559.1	0.41663	3.35E-21	postive
TNF	AC090559.1	0.405194	4.89E-20	postive
ZBP1	AC090559.1	0.453877	2.59E-25	postive
TNFSF10	AC090559.1	0.554401	2.62E-39	postive
TNFRSF1B	AC090559.1	0.749191	6.05E-86	postive
CYLD	AC090559.1	0.554515	2.52E-39	postive
CFLAR	AC090559.1	0.512829	5.96E-33	postive
FLT3	AC090559.1	0.577757	2.74E-43	postive
PLK1	AL133215.2	0.40167	1.09E-19	postive
FASLG	MMP25-AS1	0.421608	1.01E-21	postive
TNFRSF1B	LINC00892	0.452472	3.78E-25	postive
CYLD	LINC00892	0.416432	3.52E-21	postive
FLT3	LINC00892	0.492504	3.78E-30	postive
CD40	LINC00892	0.443478	4.10E-24	postive
ATRX	AL096828.3	-0.40339	7.39E-20	negative
FASLG	AL365361.1	0.48915	1.05E-29	postive

MLKL	AL365361.1	0.472673	1.37E-27	postive
TNF	AL365361.1	0.49783	7.27E-31	postive
CASP8	AL365361.1	0.478271	2.70E-28	postive
ZBP1	AL365361.1	0.701759	4.47E-71	postive
TNFSF10	AL365361.1	0.56393	6.81E-41	postive
TNFRSF1B	AL365361.1	0.609062	3.72E-49	postive
CYLD	AL365361.1	0.732129	3.14E-80	postive
CFLAR	AL365361.1	0.638793	2.29E-55	postive
FLT3	AL365361.1	0.697552	6.73E-70	postive
CD40	AL365361.1	0.650142	6.36E-58	postive
EGFR	LINC02587	0.488812	1.17E-29	postive
TRIM11	NOP14-AS1	0.499645	4.12E-31	postive
SIRT3	NOP14-AS1	0.431412	9.00E-23	postive
STUB1	NOP14-AS1	0.44736	1.48E-24	postive
ATRX	NOP14-AS1	-0.41211	9.79E-21	negative
FASLG	AC015819.1	0.527671	4.04E-35	postive
MLKL	AC015819.1	0.525343	9.00E-35	postive
RIPK3	AC015819.1	0.55828	6.02E-40	postive
TNF	AC015819.1	0.575776	6.13E-43	postive
ZBP1	AC015819.1	0.641784	4.97E-56	postive
TNFSF10	AC015819.1	0.473733	1.01E-27	postive
TNFRSF1B	AC015819.1	0.725888	3.01E-78	postive
CYLD	AC015819.1	0.509683	1.67E-32	postive
CFLAR	AC015819.1	0.539432	6.46E-37	postive
FLT3	AC015819.1	0.727949	6.76E-79	postive
CD40	AC015819.1	0.613222	5.51E-50	postive
TARDBP	NFYC-AS1	0.400356	1.47E-19	postive
FAS	CARD8-AS1	0.425542	3.87E-22	postive
TNFSF10	CARD8-AS1	0.423903	5.78E-22	postive
TNFRSF1B	CARD8-AS1	0.413331	7.34E-21	postive
CYLD	CARD8-AS1	0.548954	2.01E-38	postive
FLT3	CARD8-AS1	0.508683	2.31E-32	postive
CD40	CARD8-AS1	0.422433	8.28E-22	postive
FASLG	AC007384.1	0.485021	3.66E-29	postive
MLKL	AC007384.1	0.504456	8.97E-32	postive
RIPK3	AC007384.1	0.486455	2.38E-29	postive
TNF	AC007384.1	0.480248	1.51E-28	postive
ZBP1	AC007384.1	0.781963	2.47E-98	postive
TNFSF10	AC007384.1	0.452952	3.32E-25	postive
TNFRSF1B	AC007384.1	0.695658	2.24E-69	postive
CYLD	AC007384.1	0.562535	1.17E-40	postive
CFLAR	AC007384.1	0.592433	5.85E-46	postive
FLT3	AC007384.1	0.68685	5.39E-67	postive
CD40	AC007384.1	0.624498	2.69E-52	postive

ID1	LINC02557	0.623823	3.72E-52	postive
FADD	AP000487.1	0.656594	2.00E-59	postive
MLKL	TNFRSF10A-AS1	0.450557	6.32E-25	postive
CYLD	TNFRSF10A-AS1	0.445641	2.33E-24	postive
ATRX	AP003392.1	0.412786	8.35E-21	postive
FASLG	AL135818.1	0.851263	######	postive
MLKL	AL135818.1	0.580451	9.06E-44	postive
RIPK3	AL135818.1	0.571994	2.82E-42	postive
TNF	AL135818.1	0.526669	5.71E-35	postive
ZBP1	AL135818.1	0.74561	1.05E-84	postive
TNFSF10	AL135818.1	0.627785	5.47E-53	postive
TNFRSF1B	AL135818.1	0.819991	######	postive
CYLD	AL135818.1	0.568731	1.03E-41	postive
CFLAR	AL135818.1	0.605661	1.74E-48	postive
FLT3	AL135818.1	0.589884	1.74E-45	postive
CD40	AL135818.1	0.574576	9.97E-43	postive
RIPK3	AC124248.1	0.407	3.22E-20	postive
BACH2	AL390719.2	0.477327	3.56E-28	postive
GATA3	AL390719.2	0.891437	######	postive
MYCN	AL390719.2	0.680109	3.14E-65	postive
FLT3	AC055822.1	0.43382	4.91E-23	postive
CD40	AC055822.1	0.451143	5.40E-25	postive
FASLG	AC022706.1	0.628456	3.94E-53	postive
MLKL	AC022706.1	0.556695	1.10E-39	postive
RIPK3	AC022706.1	0.573523	1.52E-42	postive
TNF	AC022706.1	0.566594	2.40E-41	postive
ZBP1	AC022706.1	0.678703	7.23E-65	postive
TNFSF10	AC022706.1	0.541092	3.56E-37	postive
TNFRSF1B	AC022706.1	0.720662	1.25E-76	postive
CYLD	AC022706.1	0.536399	1.91E-36	postive
CFLAR	AC022706.1	0.550384	1.18E-38	postive
FLT3	AC022706.1	0.641054	7.22E-56	postive
CD40	AC022706.1	0.670175	1.03E-62	postive
MLKL	LINC02576	0.412276	9.42E-21	postive
ZBP1	LINC02576	0.743024	7.94E-84	postive
TNFRSF1B	LINC02576	0.485363	3.30E-29	postive
CYLD	LINC02576	0.541644	2.91E-37	postive
CFLAR	LINC02576	0.533849	4.70E-36	postive
FLT3	LINC02576	0.581692	5.43E-44	postive
CD40	LINC02576	0.587831	4.16E-45	postive
FASLG	AC025171.5	0.469252	3.65E-27	postive
MLKL	AC025171.5	0.450062	7.21E-25	postive

TNF	AC025171.5	0.414131	6.08E-21	postive
ZBP1	AC025171.5	0.583521	2.54E-44	postive
TNFSF10	AC025171.5	0.458264	7.84E-26	postive
TNFRSF1B	AC025171.5	0.589975	1.67E-45	postive
CYLD	AC025171.5	0.580008	1.09E-43	postive
CFLAR	AC025171.5	0.587095	5.67E-45	postive
FLT3	AC025171.5	0.623815	3.73E-52	postive
CD40	AC025171.5	0.530212	1.68E-35	postive
FASLG	LINC01943	0.831916	######	postive
MLKL	LINC01943	0.538945	7.69E-37	postive
RIPK3	LINC01943	0.456504	1.27E-25	postive
TNF	LINC01943	0.401796	1.06E-19	postive
ZBP1	LINC01943	0.622264	7.85E-52	postive
TNFSF10	LINC01943	0.567546	1.65E-41	postive
TNFRSF1B	LINC01943	0.722814	2.72E-77	postive
CYLD	LINC01943	0.459761	5.20E-26	postive
CFLAR	LINC01943	0.513115	5.43E-33	postive
FLT3	LINC01943	0.402499	9.05E-20	postive
CD40	LINC01943	0.480944	1.23E-28	postive
BACH2	AL645608.2	0.414704	5.31E-21	postive
GATA3	AL645608.2	0.555309	1.86E-39	postive
MYCN	AL645608.2	0.544638	9.83E-38	postive
FASLG	Z82188.2	0.444716	2.97E-24	postive
CYLD	U62631.1	0.420595	1.29E-21	postive
FLT3	U62631.1	0.45838	7.60E-26	postive
CD40	U62631.1	0.417001	3.07E-21	postive
FASLG	AC004585.1	0.52464	1.14E-34	postive
MLKL	AC004585.1	0.430098	1.25E-22	postive
RIPK3	AC004585.1	0.456257	1.36E-25	postive
TNF	AC004585.1	0.510663	1.21E-32	postive
ZBP1	AC004585.1	0.589129	2.40E-45	postive
TNFSF10	AC004585.1	0.502098	1.90E-31	postive
TNFRSF1B	AC004585.1	0.641325	6.29E-56	postive
CYLD	AC004585.1	0.641767	5.01E-56	postive
CFLAR	AC004585.1	0.565416	3.81E-41	postive
FLT3	AC004585.1	0.666649	7.65E-62	postive
CD40	AC004585.1	0.612097	9.26E-50	postive
TRIM11	AP003068.1	0.406694	3.46E-20	postive
SIRT3	AP003068.1	0.446932	1.66E-24	postive
TRAF2	AP003068.1	0.419197	1.81E-21	postive
ATRX	AP003068.1	-0.44248	5.32E-24	negative
FASLG	LINC01678	0.405273	4.80E-20	postive
RIPK3	LINC01678	0.436368	2.57E-23	postive
ZBP1	LINC01678	0.417513	2.72E-21	postive

TNFRSF1B	LINC01678	0.479385	1.95E-28	postive
TNFSF10	HSD11B1-AS1	0.41754	2.70E-21	postive
TNFRSF1B	HSD11B1-AS1	0.422263	8.63E-22	postive
TRIM11	AL034346.1	0.451332	5.14E-25	postive
FASLG	AC026369.3	0.807559	######	postive
MLKL	AC026369.3	0.437368	1.99E-23	postive
RIPK3	AC026369.3	0.402573	8.90E-20	postive
ZBP1	AC026369.3	0.557596	7.82E-40	postive
TNFSF10	AC026369.3	0.526526	6.00E-35	postive
TNFRSF1B	AC026369.3	0.646481	4.36E-57	postive
CYLD	AC026369.3	0.422469	8.21E-22	postive
CFLAR	AC026369.3	0.495543	1.48E-30	postive
CD40	AC026369.3	0.448359	1.13E-24	postive
CD40	RRN3P2	0.464785	1.29E-26	postive
TNFRSF1B	AL109741.1	0.498916	5.18E-31	postive
FLT3	AL109741.1	0.668496	2.69E-62	postive
CD40	AL109741.1	0.453616	2.78E-25	postive
TARDBP	AL354733.3	0.412991	7.96E-21	postive
FASLG	AL022067.1	0.496611	1.06E-30	postive
MLKL	AL022067.1	0.506294	4.98E-32	postive
ZBP1	AL022067.1	0.665698	1.31E-61	postive
TNFSF10	AL022067.1	0.443957	3.62E-24	postive
TNFRSF1B	AL022067.1	0.451222	5.29E-25	postive
CYLD	AL022067.1	0.438578	1.46E-23	postive
CFLAR	AL022067.1	0.4664	8.17E-27	postive
CD40	AL022067.1	0.41947	1.70E-21	postive
FASLG	AC023590.1	0.448586	1.07E-24	postive
MLKL	AC023590.1	0.431021	9.92E-23	postive
TNF	AC023590.1	0.401208	1.21E-19	postive
ZBP1	AC023590.1	0.553852	3.23E-39	postive
TNFSF10	AC023590.1	0.484141	4.76E-29	postive
TNFRSF1B	AC023590.1	0.63201	6.85E-54	postive
CYLD	AC023590.1	0.578389	2.11E-43	postive
CFLAR	AC023590.1	0.534711	3.47E-36	postive
FLT3	AC023590.1	0.625246	1.87E-52	postive
CD40	AC023590.1	0.634157	2.36E-54	postive
FASLG	LINC01806	0.41352	7.02E-21	postive
RIPK3	LINC01806	0.472818	1.32E-27	postive
ZBP1	LINC01806	0.464665	1.33E-26	postive
TNFSF10	LINC01806	0.566131	2.88E-41	postive
TNFRSF1B	LINC01806	0.482601	7.53E-29	postive
CYLD	LINC01806	0.474945	7.12E-28	postive
CFLAR	LINC01806	0.487122	1.95E-29	postive
FLT3	LINC01806	0.499329	4.55E-31	postive

BACH2	LINC01806	0.482566	7.61E-29	postive
GATA3	LINC01806	0.689577	1.01E-67	postive
CD40	LINC01806	0.428148	2.03E-22	postive
GATA3	MIR205HG	0.752032	6.10E-87	postive
MYCN	MIR205HG	0.450049	7.24E-25	postive
FASLG	LINC02416	0.84067	######	postive
MLKL	LINC02416	0.521228	3.64E-34	postive
ZBP1	LINC02416	0.641539	5.63E-56	postive
TNFSF10	LINC02416	0.617164	8.78E-51	postive
TNFRSF1B	LINC02416	0.705659	3.47E-72	postive
CYLD	LINC02416	0.571474	3.47E-42	postive
CFLAR	LINC02416	0.56539	3.85E-41	postive
FLT3	LINC02416	0.466769	7.37E-27	postive
CD40	LINC02416	0.494458	2.07E-30	postive
TARDBP	AL606534.1	0.401648	1.10E-19	postive
GATA3	AC006305.1	0.449643	8.06E-25	postive
MYCN	AC006305.1	0.541409	3.17E-37	postive
DDX58	AL445490.1	0.55484	2.22E-39	postive
