# Supplementary material for: Comprehensive Analysis of Necroptosis Landscape in Skin Cutaneous Melanoma for Appealing its Implications in Prognosis Estimation and Microenvironment Status
Source: J Pers Med. 2023 Jan 29;13(2):245. doi: 10.3390/jpm13020245 (PMC9962795; doi:10.3390/jpm13020245)
Supplement: Supplementary file 1 [file jpm-13-00245-s001.zip › Supplement_TableS3.pdf]

Table S3 Immune cell bubble chart between high- and low-risk group in multi-platform.

immune	cor	pvalue
B cell_TIMER	-0.13601	0.003541
T cell CD4+_TIMER	-0.38549	1.13E-17
T cell CD8+_TIMER	-0.51108	7.67E-32
Neutrophil_TIMER	-0.52025	4.00E-33
Macrophage_TIMER	-0.33825	1.01E-13
Myeloid dendritic cell_TIMER	-0.48152	5.85E-28
B cell naive_CIBERSORT	0.107534	0.021351
B cell memory_CIBERSORT	-0.12064	0.00976
T cell CD8+_CIBERSORT	-0.6037	8.09E-47
T cell CD4+ naive_CIBERSORT	0.111933	0.016554
T cell CD4+ memory resting_CIBERSORT	0.124396	0.007693
T cell CD4+ memory activated_CIBERSORT	-0.41592	1.38E-20
T cell gamma delta_CIBERSORT	-0.32425	1.13E-12
NK cell resting_CIBERSORT	0.364191	8.22E-16
NK cell activated_CIBERSORT	-0.40827	7.96E-20
Monocyte_CIBERSORT	-0.11677	0.012398
Macrophage M0_CIBERSORT	0.372327	1.66E-16
Macrophage M1_CIBERSORT	-0.41149	3.83E-20
Macrophage M2_CIBERSORT	0.246899	8.67E-08
Myeloid dendritic cell resting_CIBERSORT	-0.18057	0.000102
Myeloid dendritic cell activated_CIBERSORT	0.139246	0.002823
Mast cell activated_CIBERSORT	0.202014	1.32E-05
Eosinophil_CIBERSORT	0.117068	0.012171
B cell memory_CIBERSORT-ABS	-0.20907	6.42E-06
B cell plasma_CIBERSORT-ABS	-0.19219	3.47E-05
T cell CD8+_CIBERSORT-ABS	-0.67482	3.73E-62
T cell CD4+ naive_CIBERSORT-ABS	0.107258	0.021688
T cell CD4+ memory activated_CIBERSORT-ABS	-0.43285	2.45E-22
T cell follicular helper_CIBERSORT-ABS	-0.53349	4.78E-35
T cell regulatory (Tregs)_CIBERSORT-ABS	-0.28549	4.88E-10
T cell gamma delta_CIBERSORT-ABS	-0.35669	3.46E-15
NK cell resting_CIBERSORT-ABS	0.334231	2.05E-13
NK cell activated_CIBERSORT-ABS	-0.55919	4.93E-39
Monocyte_CIBERSORT-ABS	-0.31401	6.13E-12
Macrophage M1_CIBERSORT-ABS	-0.58414	3.00E-43
Macrophage M2_CIBERSORT-ABS	-0.40343	2.35E-19
Myeloid dendritic cell resting_CIBERSORT-ABS	-0.25143	4.92E-08
Myeloid dendritic cell activated_CIBERSORT-ABS	0.138206	0.003038
Mast cell activated_CIBERSORT-ABS	0.103777	0.026361
Mast cell resting_CIBERSORT-ABS	-0.12374	0.008024
B cell_QUANTISEQ	-0.33088	3.66E-13
Macrophage M1_QUANTISEQ	-0.42883	6.52E-22

Macrophage M2_QUANTISEQ	-0.37156	1.93E-16
Monocyte_QUANTISEQ	-0.10744	0.021467
T cell CD4+ (non-regulatory)_QUANTISEQ	0.375798	8.25E-17
T cell CD8+_QUANTISEQ	-0.70415	8.16E-70
T cell regulatory (Tregs)_QUANTISEQ	-0.29253	1.73E-10
Myeloid dendritic cell_QUANTISEQ	0.239558	2.12E-07
uncharacterized cell_QUANTISEQ	0.517655	9.30E-33
T cell_MCPCOUNTER	-0.40721	1.01E-19
T cell CD8+_MCPCOUNTER	-0.68062	1.34E-63
cytotoxicity score_MCPCOUNTER	-0.55254	5.72E-38
NK cell_MCPCOUNTER	-0.60008	3.86E-46
B cell_MCPCOUNTER	-0.4439	1.55E-23
Monocyte_MCPCOUNTER	-0.50614	3.63E-31
Macrophage/Monocyte_MCPCOUNTER	-0.50614	3.63E-31
Myeloid dendritic cell_MCPCOUNTER	-0.48347	3.33E-28
Neutrophil_MCPCOUNTER	0.189685	4.40E-05
Myeloid dendritic cell activated_XCELL	-0.5713	4.91E-41
B cell_XCELL	-0.5374	1.24E-35
T cell CD4+ memory_XCELL	-0.5188	6.41E-33
T cell CD4+ naive_XCELL	-0.49903	3.26E-30
T cell CD4+ (non-regulatory)_XCELL	-0.17202	0.000216
T cell CD4+ effector memory_XCELL	-0.18365	7.71E-05
T cell CD8+ naive_XCELL	-0.42162	3.65E-21
T cell CD8+_XCELL	-0.67661	1.35E-62
T cell CD8+ central memory_XCELL	-0.69494	2.61E-67
T cell CD8+ effector memory_XCELL	-0.33515	1.74E-13
Class-switched memory B cell_XCELL	-0.4881	8.60E-29
Myeloid dendritic cell_XCELL	-0.39075	3.71E-18
Macrophage_XCELL	-0.50585	3.98E-31
Macrophage M1_XCELL	-0.50226	1.21E-30
Macrophage M2_XCELL	-0.33765	1.12E-13
B cell memory_XCELL	-0.37863	4.64E-17
Monocyte_XCELL	-0.43135	3.53E-22
B cell naive_XCELL	-0.39444	1.69E-18
Plasmacytoid dendritic cell_XCELL	-0.58558	1.67E-43
B cell plasma_XCELL	-0.39416	1.79E-18
T cell gamma delta_XCELL	-0.12604	0.006917
T cell CD4+ Th2_XCELL	-0.13255	0.004489
T cell regulatory (Tregs)_XCELL	-0.15662	0.00077
immune score_XCELL	-0.61296	1.35E-48
microenvironment score_XCELL	-0.57952	1.92E-42
B cell_EPIC	-0.41459	1.88E-20
T cell CD8+_EPIC	-0.52465	9.38E-34
Macrophage_EPIC	-0.52937	1.93E-34

NK cell_EPIC	-0.27465	2.27E-09
uncharacterized cell_EPIC	0.371023	2.15E-16
