# Supplementary material for: Comprehensive Analysis of Necroptosis Landscape in Skin Cutaneous Melanoma for Appealing its Implications in Prognosis Estimation and Microenvironment Status
Source: J Pers Med. 2023 Jan 29;13(2):245. doi: 10.3390/jpm13020245 (PMC9962795; doi:10.3390/jpm13020245)
Supplement: Supplementary file 1 [file jpm-13-00245-s001.zip › Supplement_TableS4.pdf]

Table S4 Different Subtypes in SKCM based on expressions of 4 necroptosis-related genes.

ID	Cluster
TCGA-FS-A1Z0	C1
TCGA-EE-A2ME	C1
TCGA-EB-A6R0	C2
TCGA-EE-A2MT	C2
TCGA-WE-A8K5	C2
TCGA-D9-A1JX	C1
TCGA-FS-A1ZM	C2
TCGA-FS-A1YY	C2
TCGA-EE-A2A6	C2
TCGA-FS-A4F0	C2
TCGA-FS-A4F9	C1
TCGA-EB-A4IQ	C2
TCGA-EB-A24C	C2
TCGA-WE-A8ZR	C2
TCGA-EE-A2A2	C2
TCGA-ER-A196	C2
TCGA-DA-A1IB	C2
TCGA-D3-A8GV	C1
TCGA-XV-A9W2	C2
TCGA-W3-AA1O	C2
TCGA-FS-A4FD	C1
TCGA-EE-A2GL	C1
TCGA-EB-A1NK	C2
TCGA-BF-AAP6	C2
TCGA-EB-A85J	C2
TCGA-EE-A2MC	C2
TCGA-GN-A263	C2
TCGA-FS-A1Z3	C1
TCGA-IH-A3EA	C1
TCGA-EE-A180	C2
TCGA-EB-A550	C1
TCGA-W3-AA1Q	C1
TCGA-D3-A2J9	C1
TCGA-BF-A1Q0	C2
TCGA-EE-A2GI	C2
TCGA-EE-A29L	C2
TCGA-EE-A2ML	C1
TCGA-FS-A1ZK	C2
TCGA-GN-A4U5	C1
TCGA-EE-A2MJ	C1
TCGA-D3-A2JE	C2

TCGA-D9-A3Z1	C2
TCGA-EE-A3JI	C1
TCGA-D9-A4Z2	C1
TCGA-EB-A44Q	C2
TCGA-RP-A690	C2
TCGA-ER-A2NG	C2
TCGA-EE-A29S	C1
TCGA-ER-A19K	C2
TCGA-WE-AAA3	C1
TCGA-EB-A5UM	C1
TCGA-D3-A51T	C1
TCGA-EB-A3XD	C1
TCGA-D9-A4Z6	C1
TCGA-EE-A2MG	C1
TCGA-EB-A431	C2
TCGA-GN-A26D	C2
TCGA-FS-A1ZB	C2
TCGA-ER-A19P	C1
TCGA-D3-A3MU	C1
TCGA-D3-A8GN	C1
TCGA-EE-A2MI	C1
TCGA-ER-A2NB	C2
TCGA-EB-A5SH	C1
TCGA-D3-A2JG	C1
TCGA-ER-A19F	C2
TCGA-FW-A5DX	C2
TCGA-GF-A2C7	C1
TCGA-EE-A3AC	C2
TCGA-D3-A1QA	C2
TCGA-D3-A2JH	C1
TCGA-DA-A1I7	C1
TCGA-EE-A2GT	C2
TCGA-BF-A3DN	C1
TCGA-D3-A8GR	C2
TCGA-FS-A4F5	C2
TCGA-D9-A6E9	C1
TCGA-EE-A3JD	C2
TCGA-3N-A9WB	C2
TCGA-EB-A6L9	C2
TCGA-GF-A3OT	C2
TCGA-EE-A2MD	C2
TCGA-BF-AAP1	C1
TCGA-XV-AAZW	C1
TCGA-EB-A44N	C1

TCGA-D9-A6EC	C2
TCGA-QB-A6FS	C2
TCGA-W3-A828	C2
TCGA-XV-AAZY	C2
TCGA-DA-A1IC	C1
TCGA-EE-A17Y	C1
TCGA-EB-A553	C2
TCGA-BF-A5EQ	C2
TCGA-DA-A3F8	C1
TCGA-EE-A29B	C2
TCGA-D3-A3CF	C2
TCGA-EB-A4P0	C2
TCGA-RP-A693	C1
TCGA-DA-A95Y	C2
TCGA-QB-AA9O	C2
TCGA-EE-A2GS	C1
TCGA-FS-A4FB	C1
TCGA-D3-A8GJ	C1
TCGA-BF-A5EO	C1
TCGA-ER-A19T	C1
TCGA-BF-A9VF	C2
TCGA-GN-A4U3	C2
TCGA-FS-A1Z4	C2
TCGA-EE-A2MS	C1
TCGA-D3-A8GK	C2
TCGA-EE-A3J7	C1
TCGA-OD-A75X	C1
TCGA-DA-A95Z	C1
TCGA-FR-A7UA	C2
TCGA-EE-A184	C2
TCGA-WE-A8K1	C2
TCGA-D9-A4Z3	C2
TCGA-FS-A1ZT	C1
TCGA-ER-A19S	C1
TCGA-EE-A20C	C2
TCGA-EB-A3HV	C1
TCGA-EE-A182	C2
TCGA-GN-A26C	C2
TCGA-D3-A2JB	C2
TCGA-EB-A3XF	C1
TCGA-ER-A42K	C1
TCGA-Z2-AA3S	C2
TCGA-D3-A8GB	C2
TCGA-FR-A729	C1

TCGA-EE-A2A0	C1
TCGA-DA-A3F2	C2
TCGA-W3-A825	C1
TCGA-EE-A29W	C1
TCGA-GN-A267	C2
TCGA-EB-A42Y	C1
TCGA-Z2-A8RT	C1
TCGA-D3-A3MO	C1
TCGA-DA-A1I1	C1
TCGA-FS-A4F8	C1
TCGA-EE-A3JB	C1
TCGA-D9-A149	C2
TCGA-EB-A6QZ	C1
TCGA-ER-A198	C2
TCGA-EE-A29C	C2
TCGA-3N-A9WD	C2
TCGA-D3-A8GP	C1
TCGA-BF-AAP8	C1
TCGA-DA-A1I2	C1
TCGA-WE-A8ZY	C2
TCGA-D3-A1QB	C1
TCGA-ER-A2NC	C2
TCGA-EE-A2MP	C1
TCGA-DA-A1I0	C2
TCGA-EB-A4OY	C2
TCGA-W3-AA1V	C1
TCGA-ER-A19D	C2
TCGA-GF-A769	C1
TCGA-XV-AB01	C1
TCGA-EE-A2M7	C1
TCGA-DA-A1I4	C2
TCGA-EB-A5SE	C1
TCGA-D9-A148	C2
TCGA-EE-A2GN	C1
TCGA-EE-A2GR	C2
TCGA-FS-A1YX	C1
TCGA-EE-A29Q	C2
TCGA-FW-A3R5	C2
TCGA-EB-A6QY	C2
TCGA-BF-AAP7	C1
TCGA-EE-A2GK	C1
TCGA-EB-A3XE	C1
TCGA-EE-A20I	C2
TCGA-FS-A1ZW	C2

TCGA-FR-A8YD	C1
TCGA-W3-AA1R	C2
TCGA-YD-A89C	C2
TCGA-EB-A5SG	C1
TCGA-EE-A2MU	C2
TCGA-ER-A2NE	C2
TCGA-D3-A1Q4	C1
TCGA-DA-A95W	C1
TCGA-DA-A1HY	C2
TCGA-D3-A3CC	C1
TCGA-D3-A51K	C1
TCGA-ER-A19J	C1
TCGA-FW-A3TU	C2
TCGA-EE-A185	C2
TCGA-D3-A8GS	C1
TCGA-D3-A5GR	C1
TCGA-EE-A3J3	C1
TCGA-ER-A3PL	C1
TCGA-BF-AAOU	C1
TCGA-EE-A2GO	C1
TCGA-D3-A2JL	C2
TCGA-XV-AAZV	C1
TCGA-FR-A7U8	C1
TCGA-GN-A26A	C1
TCGA-EE-A2MK	C1
TCGA-DA-A1IA	C2
TCGA-GN-A4U7	C2
TCGA-EE-A2GH	C2
TCGA-FR-A3YN	C2
TCGA-WE-AAA4	C2
TCGA-FR-A2OS	C2
TCGA-WE-A8ZX	C2
TCGA-D3-A2JF	C1
TCGA-D3-A1Q6	C2
TCGA-EB-A299	C1
TCGA-EE-A2MH	C1
TCGA-GN-A268	C2
TCGA-D3-A2JP	C1
TCGA-FR-A8YE	C1
TCGA-D9-A3Z3	C2
TCGA-YD-A9TA	C2
TCGA-FS-A1ZD	C2
TCGA-D9-A1X3	C2
TCGA-EE-A29A	C2

TCGA-ER-A42H	C2
TCGA-EE-A2GB	C2
TCGA-BF-A5ES	C2
TCGA-EB-A5VV	C2
TCGA-BF-A3DJ	C1
TCGA-D3-A8GL	C2
TCGA-ER-A19E	C2
TCGA-GN-A4U4	C2
TCGA-GF-A4EO	C1
TCGA-EE-A29M	C2
TCGA-EE-A3JH	C1
TCGA-GF-A6C9	C2
TCGA-Z2-AA3V	C1
TCGA-D3-A1Q3	C2
TCGA-EE-A3J8	C1
TCGA-D9-A1JW	C2
TCGA-FS-A1ZY	C2
TCGA-GN-A266	C2
TCGA-EE-A181	C1
TCGA-FS-A4F2	C2
TCGA-FW-A3TV	C2
TCGA-EE-A29P	C1
TCGA-ER-A195	C1
TCGA-FS-A1ZS	C1
TCGA-BF-AAP0	C1
TCGA-ER-A2NF	C2
TCGA-FS-A4FC	C1
TCGA-FS-A1ZR	C1
TCGA-D3-A1Q1	C1
TCGA-DA-A95V	C1
TCGA-EE-A2GE	C1
TCGA-HR-A2OG	C2
TCGA-BF-A3DL	C2
TCGA-FS-A1ZJ	C1
TCGA-FW-A5DY	C1
TCGA-BF-AAP4	C2
TCGA-GN-A264	C1
TCGA-D3-A8GE	C1
TCGA-FS-A1ZG	C2
TCGA-D3-A2JD	C2
TCGA-EE-A3AB	C1
TCGA-FR-A7U9	C2
TCGA-EB-A5KH	C2
TCGA-D3-A51J	C1

TCGA-ER-A19Q	C2
TCGA-D3-A1Q9	C2
TCGA-ER-A19B	C1
TCGA-FS-A1ZH	C1
TCGA-ER-A19M	C1
TCGA-GF-A6C8	C2
TCGA-ER-A197	C1
TCGA-DA-A3F5	C2
TCGA-D3-A3C8	C1
TCGA-EE-A3JE	C1
TCGA-BF-A1PV	C2
TCGA-D3-A2J8	C2
TCGA-BF-A1PU	C1
TCGA-GN-A262	C2
TCGA-D3-A1Q5	C1
TCGA-EB-A4IS	C2
TCGA-EB-A41A	C2
TCGA-D3-A51R	C2
TCGA-EE-A17X	C2
TCGA-EE-A2MM	C1
TCGA-EE-A20H	C1
TCGA-EE-A2MN	C1
TCGA-WE-A8ZO	C1
TCGA-EB-A57M	C1
TCGA-WE-A8K6	C1
TCGA-ER-A1A1	C2
TCGA-BF-A5ER	C2
TCGA-EE-A20B	C1
TCGA-D3-A3MR	C1
TCGA-D9-A6EG	C2
TCGA-EB-A5SF	C2
TCGA-EE-A29D	C1
TCGA-EB-A5UL	C2
TCGA-EB-A97M	C2
TCGA-3N-A9WC	C1
TCGA-EE-A29X	C2
TCGA-XV-A9W5	C1
TCGA-EE-A2GD	C1
TCGA-D3-A51E	C1
TCGA-FS-A1ZN	C1
TCGA-EE-A29T	C1
TCGA-EE-A2M5	C2
TCGA-FR-A69P	C1
TCGA-BF-AAP2	C2

TCGA-HR-A2OH	C1
TCGA-EE-A2MQ	C2
TCGA-FR-A44A	C1
TCGA-DA-A3F3	C2
TCGA-D3-A51F	C1
TCGA-FS-A1ZA	C2
TCGA-D3-A2JA	C2
TCGA-EE-A29N	C1
TCGA-EB-A24D	C1
TCGA-EE-A3JA	C2
TCGA-D3-A3C7	C2
TCGA-RP-A694	C2
TCGA-EB-A4OZ	C1
TCGA-FS-A1ZP	C1
TCGA-GN-A4U9	C1
TCGA-WE-AA9Y	C1
TCGA-EE-A2A5	C1
TCGA-EE-A2M6	C1
TCGA-D3-A2JK	C1
TCGA-D3-A1Q7	C1
TCGA-ER-A19A	C1
TCGA-DA-A960	C1
TCGA-GN-A4U8	C1
TCGA-EE-A3AD	C1
TCGA-FR-A3R1	C2
TCGA-EE-A183	C2
TCGA-EE-A29E	C2
TCGA-EE-A29H	C2
TCGA-FS-A1ZZ	C2
TCGA-EE-A3AG	C1
TCGA-GN-A9SD	C1
TCGA-EE-A2GU	C1
TCGA-ER-A3ET	C2
TCGA-YG-AA3O	C1
TCGA-EE-A2MF	C2
TCGA-EB-A44O	C2
TCGA-FS-A1ZQ	C1
TCGA-EE-A3J4	C1
TCGA-FR-A726	C1
TCGA-FS-A1ZF	C2
TCGA-FS-A1ZC	C2
TCGA-WE-A8ZM	C1
TCGA-ER-A193	C1
TCGA-W3-AA21	C1

TCGA-D9-A3Z4	C1
TCGA-WE-A8ZN	C2
TCGA-WE-A8ZQ	C1
TCGA-BF-A1PZ	C2
TCGA-ER-A2ND	C2
TCGA-D3-A5GN	C1
TCGA-XV-A9VZ	C2
TCGA-EB-A5UN	C2
TCGA-EE-A3AA	C2
TCGA-FR-A728	C1
TCGA-HR-A5NC	C1
TCGA-DA-A1HV	C1
TCGA-GN-A8LL	C2
TCGA-ER-A42L	C2
TCGA-D3-A8GQ	C2
TCGA-W3-AA1W	C1
TCGA-EB-A41B	C2
TCGA-WE-A8JZ	C2
TCGA-D3-A1Q8	C1
TCGA-D9-A6EA	C1
TCGA-EB-A5FP	C2
TCGA-EB-A82B	C2
TCGA-EB-A3Y7	C2
TCGA-ER-A19H	C1
TCGA-D3-A3C6	C1
TCGA-D3-A3MV	C2
TCGA-ER-A19W	C1
TCGA-EE-A17Z	C1
TCGA-FS-A4F4	C2
TCGA-ER-A199	C1
TCGA-D3-A5GT	C2
TCGA-WE-A8K4	C2
TCGA-FS-A1Z7	C1
TCGA-DA-A1HW	C1
TCGA-D3-A5GU	C1
TCGA-GN-A8LK	C2
TCGA-EE-A2M8	C1
TCGA-GN-A8LN	C2
TCGA-EE-A3AF	C2
TCGA-EB-A42Z	C1
TCGA-D3-A3CE	C1
TCGA-EE-A20F	C2
TCGA-WE-A8ZT	C1
TCGA-EE-A2GC	C1

TCGA-BF-A5EP	C2
TCGA-D9-A4Z5	C2
TCGA-EB-A3XB	C1
TCGA-EB-A5VU	C1
TCGA-D3-A51H	C2
TCGA-D3-A3BZ	C1
TCGA-EE-A29R	C1
TCGA-BF-AAOX	C2
TCGA-ER-A3ES	C2
TCGA-ER-A2NH	C1
TCGA-EB-A551	C2
TCGA-FR-A8YC	C1
TCGA-GN-A265	C2
TCGA-D3-A5GS	C1
TCGA-D3-A8GC	C2
TCGA-EE-A2MR	C2
TCGA-EB-A51B	C2
TCGA-D3-A5GO	C2
TCGA-ER-A19C	C2
TCGA-ER-A19G	C2
TCGA-EE-A29V	C2
TCGA-FS-A1ZE	C2
TCGA-EB-A85I	C1
TCGA-D3-A2J7	C2
TCGA-D3-A8GM	C2
TCGA-ER-A3EV	C1
TCGA-EE-A2GM	C2
TCGA-EB-A44P	C1
TCGA-D3-A2JO	C2
TCGA-EE-A3AH	C1
TCGA-DA-A1I8	C1
TCGA-EB-A82C	C1
TCGA-WE-AAA0	C2
TCGA-FW-A3I3	C1
TCGA-EE-A2GP	C1
TCGA-BF-A3DM	C1
TCGA-ER-A19L	C1
TCGA-LH-A9QB	C2
TCGA-DA-A95X	C1
TCGA-FS-A1ZU	C1
TCGA-D3-A2JC	C1
TCGA-D3-A8GD	C2
TCGA-D3-A2J6	C2
TCGA-ER-A194	C2

TCGA-EE-A2GJ	C1
TCGA-BF-A1PX	C1
TCGA-EB-A4XL	C1
TCGA-D3-A3CB	C1
TCGA-EE-A2A1	C1
TCGA-FS-A1YW	C1
TCGA-D3-A51N	C2
TCGA-YG-AA3N	C2
TCGA-EE-A3J5	C1
TCGA-DA-A1I5	C1
TCGA-EB-A44R	C1
TCGA-YG-AA3P	C2
TCGA-W3-A824	C1
TCGA-D3-A8GI	C1
TCGA-D3-A5GL	C2
TCGA-EE-A29G	C2
TCGA-EB-A3XC	C2
TCGA-D3-A2JN	C1
TCGA-D3-A3ML	C1
TCGA-ER-A19N	C2
TCGA-EB-A3Y6	C2
