# Supplementary material for: Comprehensive Analysis of Necroptosis Landscape in Skin Cutaneous Melanoma for Appealing its Implications in Prognosis Estimation and Microenvironment Status
Source: J Pers Med. 2023 Jan 29;13(2):245. doi: 10.3390/jpm13020245 (PMC9962795; doi:10.3390/jpm13020245)
Supplement: Supplementary file 1 [file jpm-13-00245-s001.zip › Supplement_TableS5.pdf]

Table S5 The heat map of immune cells in clusters.

immune	pvalue
T cell CD4+_TIMER	0.006243
Neutrophil_TIMER	0.00254
Macrophage_TIMER	5.75E-06
B cell naive_CIBERSORT	0.00264
T cell CD8+_CIBERSORT	0.041608
T cell CD4+ memory activated_CIBERSORT	0.030733
Monocyte_CIBERSORT	0.00053
Macrophage M0_CIBERSORT	0.006627
Myeloid dendritic cell resting_CIBERSORT	0.009105
B cell naive_CIBERSORT-ABS	0.035257
T cell CD8+_CIBERSORT-ABS	0.001351
T cell CD4+ memory resting_CIBERSORT-ABS	0.034098
T cell CD4+ memory activated_CIBERSORT-ABS	0.018583
T cell follicular helper_CIBERSORT-ABS	0.016648
NK cell activated_CIBERSORT-ABS	0.010351
Monocyte_CIBERSORT-ABS	2.12E-06
Macrophage M1_CIBERSORT-ABS	0.000374
Macrophage M2_CIBERSORT-ABS	2.99E-05
Myeloid dendritic cell resting_CIBERSORT-ABS	0.001248
Macrophage M1_QUANTISEQ	0.000234
Monocyte_QUANTISEQ	8.84E-11
Neutrophil_QUANTISEQ	0.000353
NK cell_QUANTISEQ	5.43E-09
T cell CD4+ (non-regulatory)_QUANTISEQ	3.46E-10
T cell CD8+_QUANTISEQ	0.006207
T cell regulatory (Tregs)_QUANTISEQ	0.000222
Myeloid dendritic cell_QUANTISEQ	5.51E-07
uncharacterized cell_QUANTISEQ	3.56E-05
T cell CD8+_MCPCOUNTER	0.001758
cytotoxicity score_MCPCOUNTER	1.29E-08
NK cell_MCPCOUNTER	0.000105
Monocyte_MCPCOUNTER	0.0004
Macrophage/Monocyte_MCPCOUNTER	0.0004
Myeloid dendritic cell_MCPCOUNTER	6.27E-06
Endothelial cell_MCPCOUNTER	2.07E-08
Cancer associated fibroblast_MCPCOUNTER	0.016205
T cell CD4+ memory_XCELL	0.025517
T cell CD4+ effector memory_XCELL	0.01492
T cell CD8+_XCELL	0.005311
T cell CD8+ central memory_XCELL	0.023667
Myeloid dendritic cell_XCELL	0.017834
Endothelial cell_XCELL	0.000337

Hematopoietic stem cell_XCELL	4.25E-05
Monocyte_XCELL	0.002852
T cell NK_XCELL	0.006399
T cell CD4+ Th1_XCELL	0.003256
T cell regulatory (Tregs)_XCELL	0.001741
stroma score_XCELL	0.000496
T cell CD4+_EPIC	0.039428
T cell CD8+_EPIC	8.43E-07
Endothelial cell_EPIC	3.04E-05
Macrophage_EPIC	6.37E-05
uncharacterized cell_EPIC	7.17E-06
